# Ion pair-induced conformational motion in calix[4]arene-strapped calix[4]pyrroles†
†Electronic supplementary information (ESI) available: Synthetic details, NMR spectroscopic data, calculation data for modeling, and X-ray crystal data for **1**·(ethyl acetate)_3_, **1**·CHCl_3_·(EtOH)_2_, **1**·TEAF·(CH_2_Cl_2_)_3_, **1**·CsF·(CHCl_3_)_2_·(CH_3_OH)_2_, **1**_2_·(CsCl)_3_·(C_2_H_5_OH)_2_·CHCl_3_, **2**·(CH_3_CN)_3_, **2**_2_·(TEAF)_2_·(CH_2_Cl_2_)_3_·(H_2_O)_3_, **2**·CsF·CH_3_OH·CHCl_3_, and **2**·CsCl·CH_3_OH·CHCl_3_. CCDC 1025273–1025281. For ESI and crystallographic data in CIF or other electronic format see DOI: 10.1039/c4sc03272a



**DOI:** 10.1039/c4sc03272a

**Published:** 2014-12-03

**Authors:** Sung Kuk Kim, Vincent M. Lynch, Benjamin P. Hay, Jong Seung Kim, Jonathan L. Sessler

**Affiliations:** a Department of Chemistry , The University of Texas at Austin , 105 E. 24th, Street-Stop A5300 , Austin , Texas 78712-1224 , USA; b Supramolecular Design Institute , Oak Ridge , TN 37830-7185 , USA; c Department of Chemistry , Korea University , Seoul 136-701 , Korea

## Abstract

In order to understand the still-poorly understood interplay between calix[4]arene conformations and cation and anion recognition in multicomponent systems, the ion pair receptors **1** and **2** were synthesized. In solution and in the solid state, the calix[4]arene subunit of receptor **1** adopts a cone conformation, while that of **2** interconverts between the cone and the partial cone conformation. These geometric features differ from previous systems where the calix[4]arene moiety was locked in the 1,3-alternate conformation. A combination of ^1^H NMR spectroscopic analyses and single crystal X-ray diffraction studies reveal that receptor **1** binds the fluoride and the chloride anion *via* significantly different binding modes, displaying, for instance, 1 : 1 and 2 : 3 binding stoichiometries with CsF and CsCl, respectively. In the case of **2**, the conformation of the calix[4]arene constituent of **2** is highly dependent on the size and quantity of anions present. For example, upon treatment of **2** with the fluoride anion (as both the TBA^+^ and Cs^+^ salts), the calix[4]arene unit coexists as cone and partial cone conformers that are inter-convertible. In the presence of excess CsF, the aromatic rings of the calix[4]arene subunit becomes locked in the pinched cone conformation with the result that an ion pair-mediated coordination polymer is formed. In the presence of excess CsCl, the calix[4]arene unit of **2** adopts only the partial cone conformation stabilized by aryl CH–anion hydrogen bonding interactions. The present systems constitute a rare set of related receptors wherein the effects of conformational changes are so tightly coupled with ion recognition.

## Abstract 2

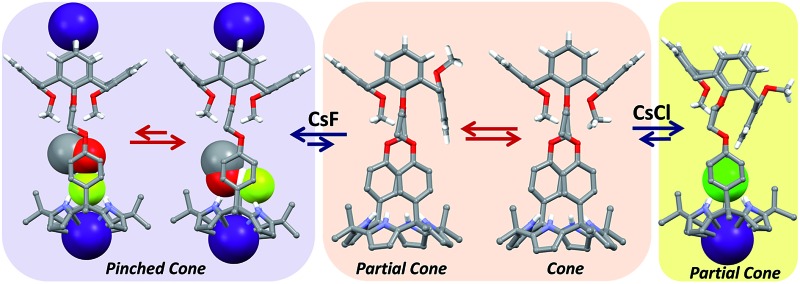
Cone- and conformationally mobile calix[4]arene-strapped calix[4]pyrroles bind cesium salts *via* various different binding modes.

## Introduction

Since the early days of supramolecular chemistry, effort has been devoted to understanding, modulating, and exploiting the conformational motion of calix[4]arenes in the context of cation recognition.^1–3^ As a result, the basic relationships between receptor conformation and the affinities and selectivities for simple cationic guests are now well established.^1–3^ For example, 1,3-dimethoxy calix[4]arene-crown-5 and crown-6 are conformationally flexible systems that adopt all three possible limiting conformations, the so-called cone, partial cone, and 1,3-alternate conformation, in the absence of a cationic guest.^2^ These conformations are inter-convertible with the cone conformation being most favorable in most organic media.^2^ However, in the presence of K^+^ and Cs^+^, these calix[4]arenes become fixed in the 1,3-alternate conformation, a geometry that provides the best fit for these cations.^2^ Less clear, however, is how the conformational features of calix[4]arenes will be manifest when they are incorporated as individual components within more elaborate receptor systems. Recently, we reported a series of calix[4]arene–calix[4]pyrrole hybrids that act as ion pair receptors.^4,5^ In these systems, the pyrrolic NH protons typically serve as the anion binding site, with both the electron rich “bowl” of the calix[4]pyrrole in its anion-bound cone conformation and the calix[4]arene moiety acting as the cation recognition sites.^4,5^ In the calix[4]arene–calix[4]pyrrole ion pair recognition systems reported to date, the calix[4]arene moiety has been locked into the 1,3-alternate conformation through steric means (*e.g.*, use of tethering crown ethers or *O*-propoxy substitution).^4,5^ It is thus an open question as to how, if at all, the conformational motion of a less-hindered calix[4]arene within a calix[4]arene–calix[4]pyrrole hybrid would be affected by ion pair recognition. Of particular interest is whether the putative calix[4]arene conformation effects, to the extent they are observed, would be regulated predominantly by cation recognition, anion binding, or a combination thereof. In a related vein, we were curious to explore whether conformational effects could be used to fine-tune the ion pair binding selectivities of ostensibly similar calix[4]arene–calix[4]pyrrole hybrid receptor systems. To address these questions, we have now prepared the cone-locked and conformationally mobile calix[4]arene-strapped calix[4]pyrroles **1** and **2**. As detailed below, these two systems are characterized by ion binding properties that are not only very different from one another, but also from our previously reported 1,3-alternate calix[4]arene-containing hybrid system **3**.^4^ In the new calix[4]arene–calix[4]pyrrole systems, and in contrast to simple conformationally flexible calix[4]arenes, both anion and cation binding play a key, structure-determining role. The present results serve to highlight how the interplay between conformational effects and substrate recognition may be used to modulate the inherent selectivities of a venerable cation recognition system, namely calix[4]arene.
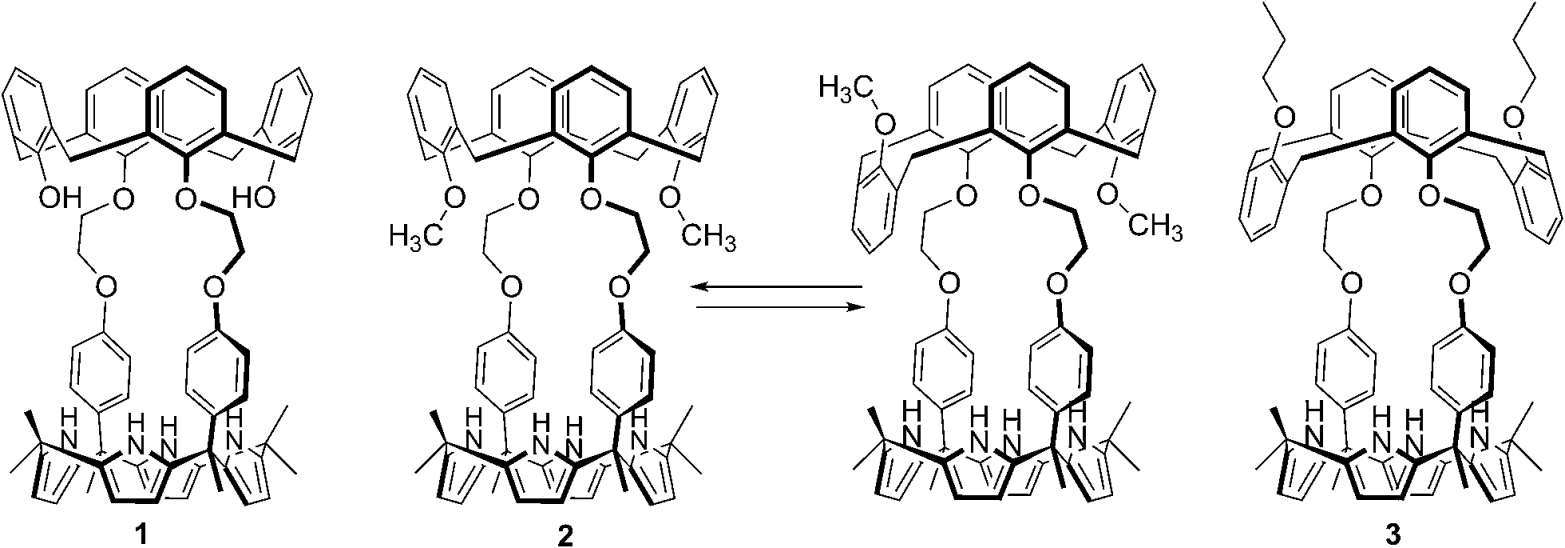


## Results and discussion

**Fig. 1:**
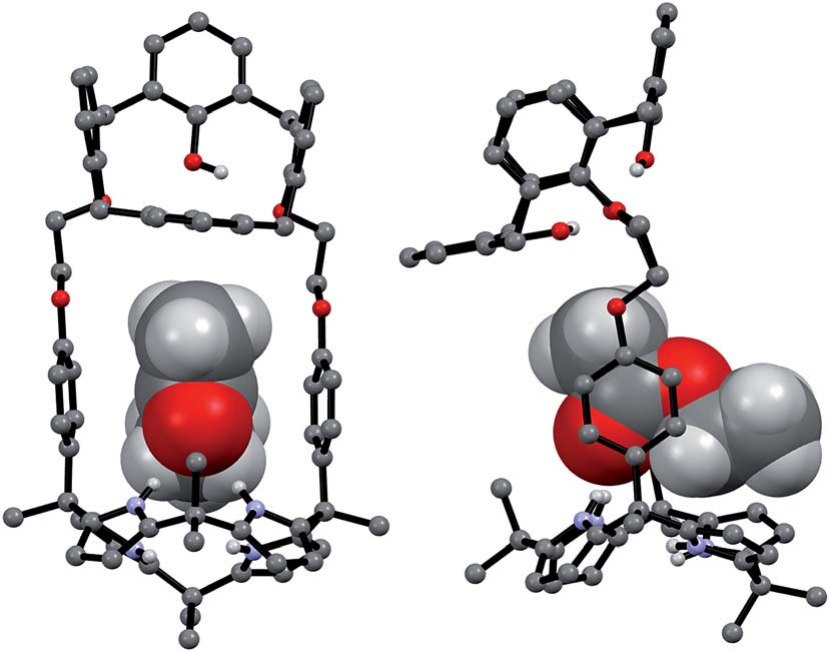
Front and side views of the single crystal X-ray diffraction structure of **1**·ethyl acetate. Most hydrogen atoms have been omitted for clarity.

**Fig. 2:**
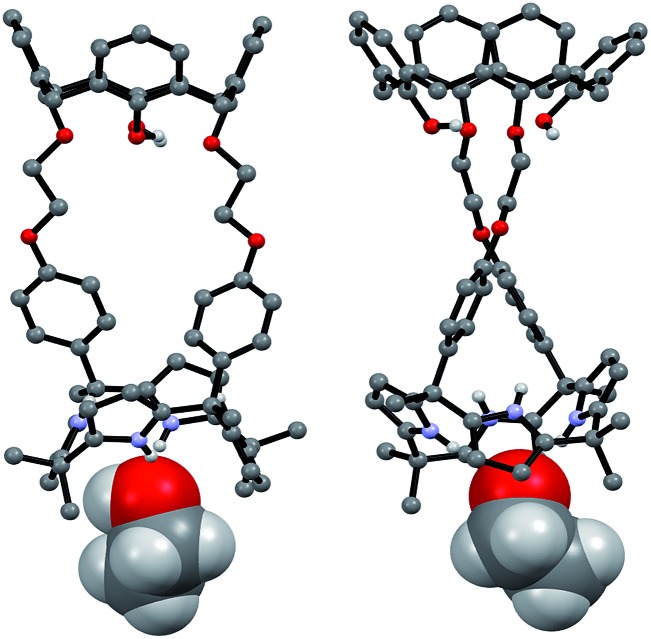
Front and side views of the single crystal X-ray diffraction structure of **1**·CH_3_CH_2_OH. Most hydrogen atoms have been omitted for clarity.

The synthetic route for receptors **1** and **2** is shown in Scheme S1.† Briefly, calix[4]arene **4** was reacted with bromoethyl dipyrromethane **5** in the presence of 1.2 equiv of K_2_CO_3_ and a catalytic amount of NaI to afford **6** in *ca.* 50% yield (Scheme S1†). Subsequently, the dipyrromethane intermediate (**6**) was condensed with acetone in the presence of 1.0 equiv of BF_3_·OEt_2_ to produce 1,3-dihydroxycalix[4]arene-strapped calix[4]pyrrole **1** in 20% yield.^4^ Reaction of **1** with an excess of iodomethane in the presence of Cs_2_CO_3_ gave the 1,3-dimethoxycalix[4]arene-strapped calix[4]pyrrole **2** in 65% yield. Compounds **1** and **2** were fully characterized by standard spectroscopic methods, as well as by single crystal X-ray structural analysis. The ^1^H NMR spectrum of **1** recorded in CDCl_3_ is well-defined and characterized by two doublet peaks for the methylene bridge proton resonances of the calix[4]arene at 3.42 and 4.35 ppm in an AB pattern (see the ESI†). This finding supports the proposition that the calix[4]arene unit adopts a cone conformation as the result of hydrogen bonding interactions between the hydroxy groups and the oxygen atoms of the neighboring aryl rings. This presumption was further supported by two single crystal X-ray diffraction analyses that revealed the calix[4]arene exists in the cone conformation with the calix[4]pyrrole adopting a flattened 1,2-alternate or a 1,3-alternate conformation depending on the bound solvent (Fig. 1 and 2).

**Fig. 3:**
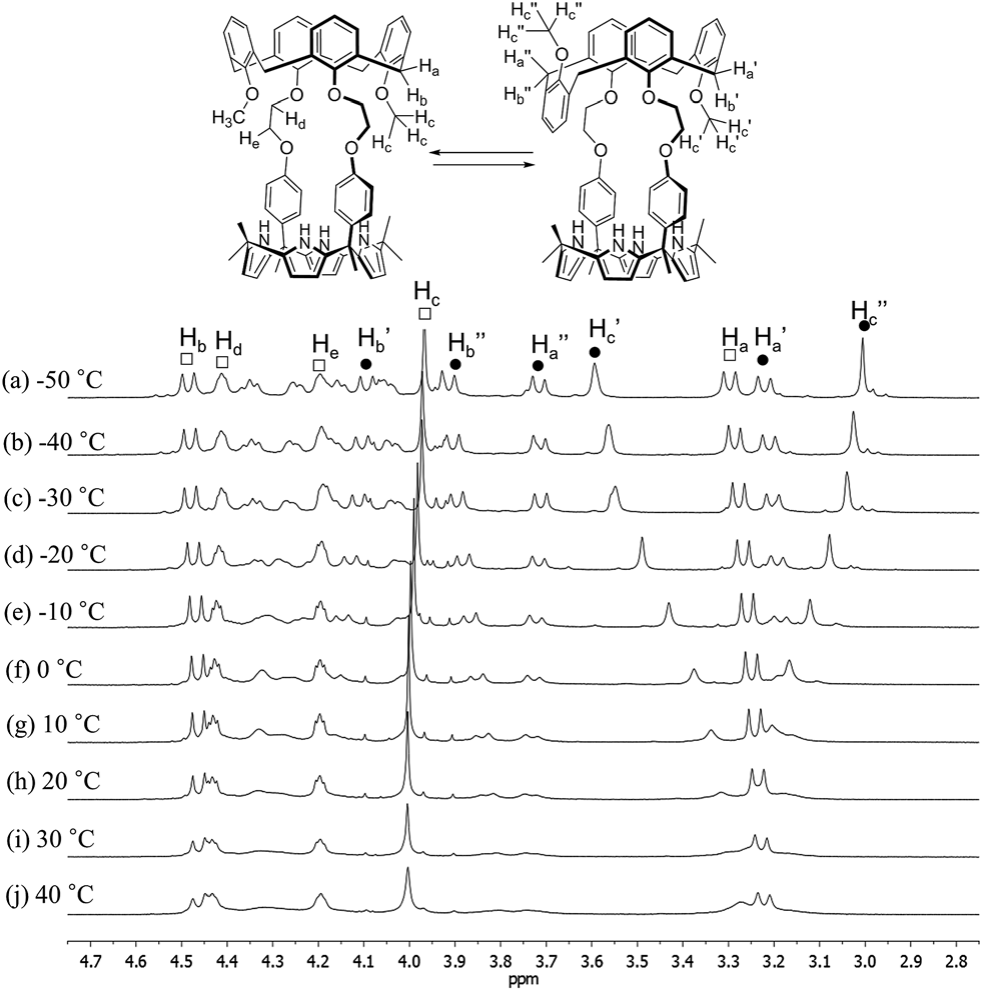
Partial ^1^H NMR spectra of **2** recorded in CDCl_3_ at various temperatures (peak designations: □ = cone; = partial cone).

In contrast to what proved true for **1**, the ^1^H NMR spectrum of receptor **2** recorded at room temperature in CDCl_3_ revealed the presence of both sharp and broad peaks over the full chemical shift range; the same proved true when DMSO-*d*_6_ was used as the solvent (Fig. 3, S1 and S2†). This combination of features was taken as an indication that two or more conformers are present in the solutions. Detailed temperature dependent ^1^H NMR spectroscopic analyses carried out separately in these two solvents (*cf.* Fig. 3, S1 and S2†) revealed that the calix[4]arene unit of **2** adopts only two conformations (cone and partial cone). That is, only two sets of signals are observed within the error range of the ^1^H NMR analysis. Moreover, no proton signal characteristic of the 1,3-alternate conformer was observed.^4^ This finding, which stands in contrast to what is true for simple calix[4]arene derivatives, is attributable to the rigid linkages between the calix[4]arene and the calix[4]pyrrole that make **2** structurally more rigid than the corresponding, unstrapped calix[4]arene. A more global analysis of the ^1^H NMR spectra provided further evidence for this conclusion. For instance, the proton signals of the calix[4]arene aromatic rings connected with the phenoxy spacers appear considerably upfield-shifted as compared with those of the methoxy benzene rings or other simple (and conformationally flexible) calix[4]arene derivatives. This upfield shift is ascribed to the fact that in **2** the aromatic rings linked to the spacers are brought relatively close together, such that the relevant protons experience a ring current effect (Fig. S1†). This increased proximity serves to lock the calix[4]arene moiety present in **2** such that there is a higher energetic barrier for conformational inter-conversion between the different conformers than in the case of analogous unstrapped calix[4]arene derivatives.

At temperatures lower than –10 °C, the proton signals of the two conformers are clearly distinguishable (Fig. 3 and S1†). On the contrary, at room temperature, the proton signals of the cone conformer remain sharp while those of the partial cone conformer appear broadened (Fig. 3 and S1†). This leads us to suggest that at room temperature the cone conformer is kinetically stable on the NMR time scale, while the partial cone conformer is structurally mobile. The observation of two distinct sets of peaks is consistent with these two limiting species undergoing relatively slow conformational inter-conversion under the conditions of the experiment (and time scale associated with the measurement). With increasing temperature, the broadened proton signals become gradually sharper. By 100 °C a well-defined spectrum is observed that is characteristic of the cone conformation (Fig. S2†). This finding supports the conclusion that at high temperatures interconversion between the conformations is fast on the NMR time scale (Fig. S2†).

**Fig. 4:**
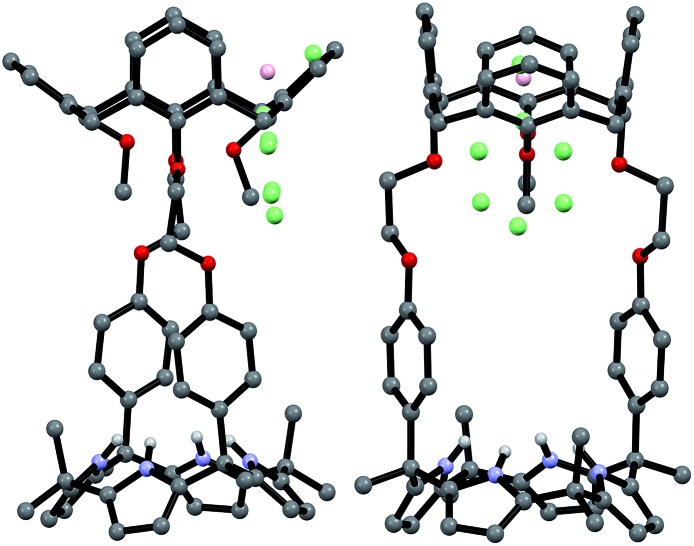
Front and side views of the single crystal X-ray structure of receptor **2** (ion-free form). One of the two methoxyphenyl groups was disordered resulting in a mixture of the cone and partial cone conformations being present in a 83 : 17 ratio (cone : partial cone). The atoms of the disordered methoxyphenyl group are shown as spheres (light green: carbon atom; pink: oxygen atom). Most hydrogen atoms and solvent molecules have been removed for clarity.

Evidence for the presence of both the partial cone and cone conformers in the solid state came from a single crystal X-ray structural analysis. Suitable single crystals were obtained by subjecting a methylene chloride/DMSO/acetonitrile solution of **2** to slow evaporation. The resulting structure revealed that the ratio of cone/partial cone conformers for the calix[4]arene subunit in **2** was approximately 83 : 17 in the solid state (Fig. 4). The calix[4]pyrrole unit adopts the cone conformation with an acetonitrile molecule forming hydrogen bonds with the pyrrolic NH protons (Fig. 4).

We then investigated the fluoride and chloride ion pair binding properties of **1** and **2** *via* ^1^H NMR spectroscopy. This was done using the corresponding tetrabutylammonium and cesium salts. In analogy to what was seen for ion pair receptor **3** and most other strapped calix[4]pyrroles bearing rigid phenyl spacers on the *meso*-positions, both **1** and **2** were found to bind the fluoride anion with high selectivity and affinity in CDCl_3_ when the relatively non-coordinating tetrabutylammonium cation (TBA^+^) was used. For instance, significant chemical shift changes in the ^1^H NMR spectra of **1** and **2** were observed when the receptors were exposed to tetrabutylammonium fluoride, TBAF (Fig. S3 and S4†). However, other analogous halide anion or the nitrate anion salts caused no appreciable chemical shift changes in the ^1^H NMR spectra of **1** and **2** under identical conditions of analysis (Fig. S5 and S6†). Such findings lead us to conclude that receptors **1** and **2** are highly selective for the fluoride anion, at least when studied as the TBA^+^ salts in CDCl_3_.

Upon subjecting compounds **1** and **2** to titration with TBAF in CDCl_3_, two sets of distinguishable signals were observed for all proton resonances, especially those associated with the calix[4]pyrrole subunits, before saturation was reached upon the addition of 1.16 and 1.06 equiv of TBAF to solutions of **1** and **2**, respectively (Fig. S3 and S4†). The observation of two sets of signals is thought to reflect the presence of both the ion-free and fluoride-bound forms of **1** and **2**. Thus, these findings support the conclusion that the kinetics of fluoride anion binding and release are slow on the NMR time scale. Presumably, this slow exchange reflects the strong binding interactions between the calix[4]pyrrole receptors **1** and **2** and the fluoride anion. This proposition was further supported by the finding that the NH peak, a singlet before the addition, becomes split into a doublet upon the addition of TBAF, as would be expected for a system wherein the bound fluoride anion is magnetically coupled to the pyrrolic NH protons (Fig. S3 and S4†).^6^ The stoichiometry (1 : 1) and association constant (*K*_a_) for the interaction of **1** with the fluoride anion was estimated to be ≈2600 M^–1^ from the ^1^H NMR spectroscopic titration data.^7^

**Fig. 5:**
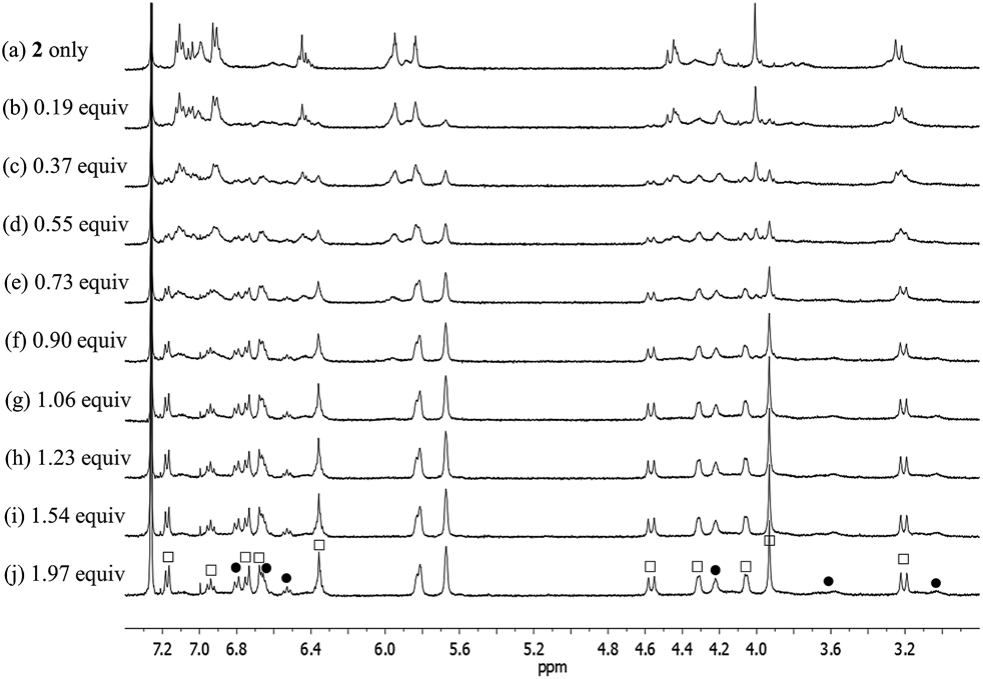
Partial ^1^H NMR spectra for the titration of receptor **2** with TBAF (tetrabutylammonium fluoride) in CDCl_3_ (peak designations: □ = cone; = partial cone).

The ^1^H NMR spectra data for the fluoride complexes obtained following the above titrations are consistent with the conformations of both the calix[4]arene and the calix[4]pyrrole of **1** being fixed in the cone conformations, whereas the calix[4]arene subunit of **2** remains conformationally mobile with evidence of interconversion between the cone and partial cone conformations being observed (Fig. S3, S4 and S7†). Specifically, the ^1^H NMR spectrum of **2**, recorded after saturation with the fluoride anion, is characterized by two sets of proton resonances corresponding to the cone and the partial cone conformer of the calix[4]arene unit, respectively (Fig. 5). In analogy to what was seen for the ion-free form of **2**, the proton signals of the cone conformer appear sharp, while those of the partial cone conformer are very broad. This is as expected for a species that is subject to some conformational mobility at room temperature (Fig. 5 and S4†). A 2D NMR (COSY) spectral analysis was also carried out and provided further evidence for the presence of two chemical species, namely the cone and partial cone forms (*cf.* Fig. S8†). The proton signals of the cone conformer were fully assigned based on a 2D NMR spectral analysis (*cf.* ESI†).

**Fig. 6:**
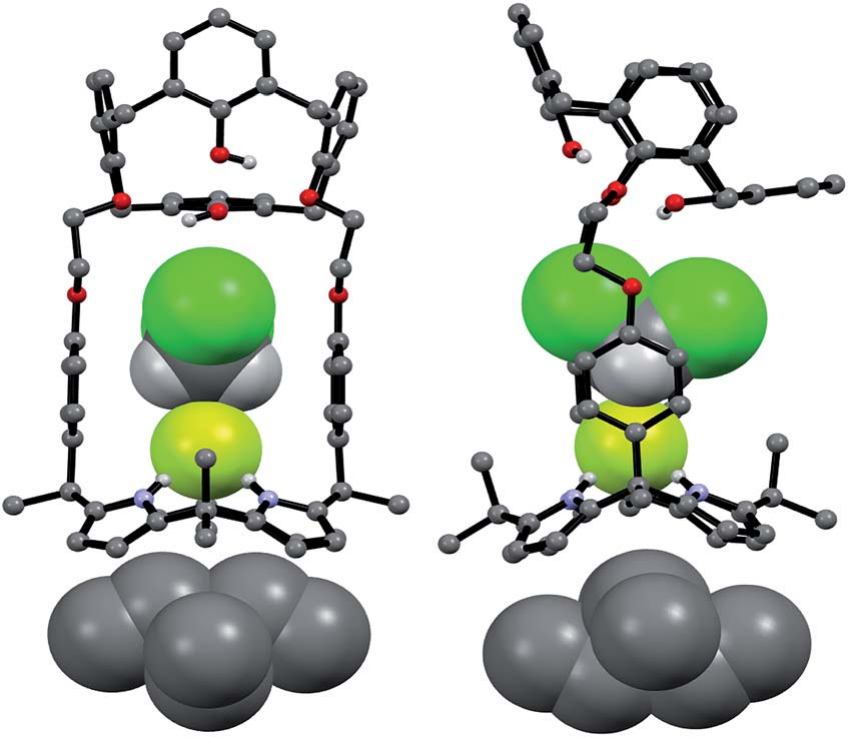
Front and side views of the single crystal X-ray diffraction structure of **1**·TEAF·CH_2_Cl_2_. Most hydrogen atoms and solvent molecules not involved in ion pair complexation have been removed for clarity.

**Fig. 7:**
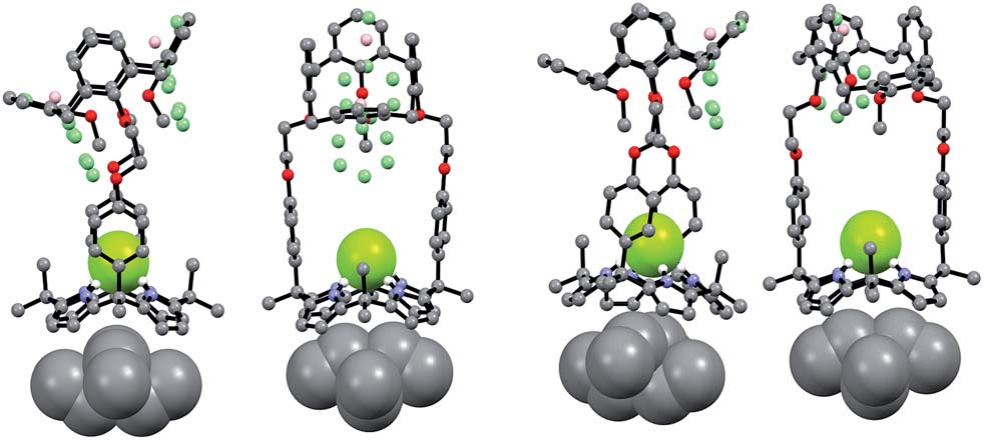
Front and side views of the X-ray structures resulting from the two different TEAF complexes of receptor **2** found in the same unit cell. Three of the four methoxyphenyl groups were disordered resulting in an approximately 3 : 2 mixture of the cone and partial cone conformations. The atoms of the disordered methoxyphenyl group are shown as spheres (light green: carbon atom; pink: oxygen atom). Most hydrogen atoms and solvent molecules have been omitted for clarity.

Support for the differing conformational behavior of **1** and **2** observed in the presence of the fluoride anion came from single crystal X-ray diffraction analyses. In both cases, diffraction grade crystals of the tetraethylammonium fluoride (TEAF) complexes were analyzed. Consistent with what was seen in solution, the resulting crystal structures revealed that both the calix[4]arene and the calix[4]pyrrole subunit of **1** adopt the cone conformation in the solid state with the fluoride anion being bound to the pyrrolic NH protons (Fig. 6). One methylene chloride molecule is also involved in the fluoride anion binding (Fig. 6). In contrast, in the case of receptor **2**, two different TEAF complexes having different torsional angles for the ethylene linkers (–O–C–C–O–) were found in the asymmetric unit of the crystals (Fig. 7). These latter crystal structures also revealed that the calix[4]arene unit of receptor **2** can adopt either the cone or the partial cone conformation after complexation with the fluoride anion and that the two resulting conformers coexist in an approximately 2 : 3 ratio in favor of the partial cone conformation in the solid state (Fig. 7).

**Fig. 8:**
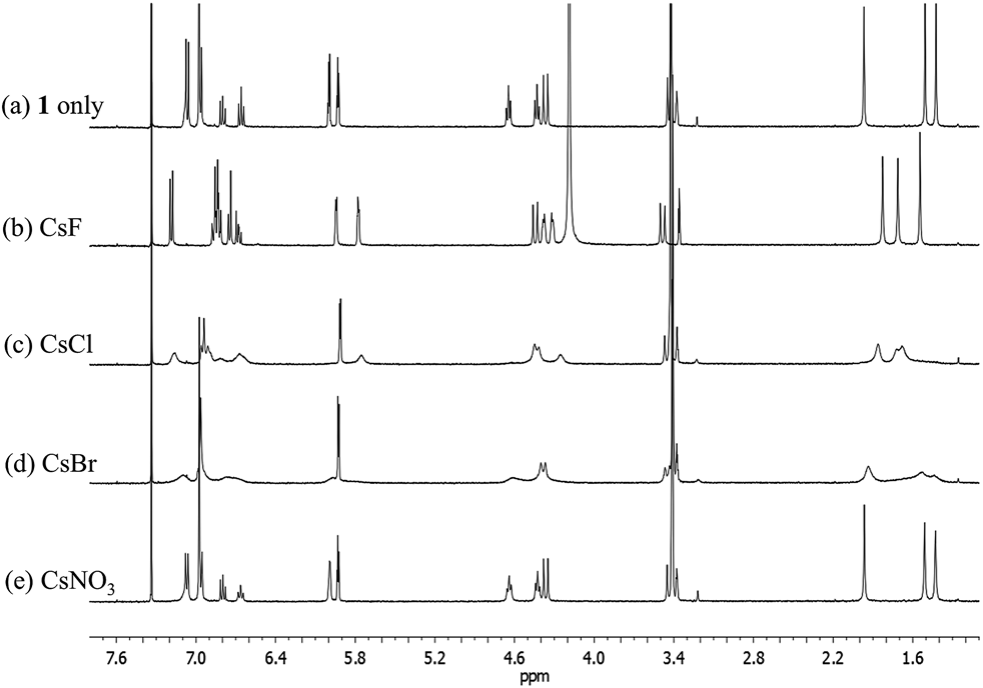
^1^H NMR spectra of (a) **1** only, (b) **1** with excess CsF, (c) **1** with excess CsCl, (d) **1** with excess CsBr, and (e) **1** with excess CsNO_3_ in CD_3_OD–CDCl_3_ (1 : 9, v/v).

Significantly different binding behavior was observed when the cesium cation, a species known to form a complex with calix[4]pyrrole analogues,^4^ was used as the counter cation for the halide and nitrate anions. For example, addition of CsF and CsCl to a solution of **1** in CD_3_OD–CDCl_3_ (1/9, v/v) the same solvent mixture previously used to study the interaction of these salts with receptor **3** (ref. 4) induces remarkable chemical shift changes in the proton resonances of both the calix[4]pyrrole and calix[4]arene moieties in the associated ^1^H NMR spectra (Fig. 8). For instance, in both cases, two sets of distinguishable peaks were seen for all proton signals of receptor **1** in the ^1^H NMR spectra measured before saturation was reached (Fig. S9 and S10†). Such findings, which stand in contrast to what was seen with the corresponding TBA^+^ salts (*vide supra*), are considered reflective of the fact that receptor **1** binds both the fluoride and chloride anions well under these solution phase conditions. This is presumed to reflect the synergistic effect associated with cesium cation-based ion pair complexation.

Based on the upfield shifts seen for the ethylene likers of **1**, complexation of the Cs^+^ cation occurs within the calix[4]pyrrole bowl, rather than being mediated by the ether linkages, as seen in the case of control system **3**.^4^ From the ^1^H NMR titration data, a binding constant of >10^4^ M^–1^ and a 1 : 1 binding stoichiometry was determined for the association of **1** with CsF in 10% CD_3_OD in CDCl_3_.^7^

During the ^1^H NMR spectral titration of **1** with CsCl, peak broadening was observed for all proton resonances of both the ion-free form and the CsCl complex of **1** (Fig. S10†). This observation leads us to suggest that the CsCl binding mode differs from that seen in the case of CsF. Based on the specifics of the salt-induced changes in the chemical shifts, a second interaction between the CsCl complexes of **1** and its ion-free form is inferred.

No appreciable chemical shift changes associated with ion binding are seen upon adding CsBr or CsNO_3_ to receptor **1** in 10% CD_3_OD in CDCl_3_. This stands in marked contrast to what was observed in the case of the control system **3**, which binds these salts well under these solution phase conditions.^4^ The peak broadening seen upon treatment with CsBr is ascribed to the changes in the solvent polarity that occur when excess CsBr is added to the solvent mixture, rather than to the complexation of CsBr by **1** (Fig. 8). Similar broadening of the ^1^H NMR spectral features was also seen when **1** was exposed to excess LiCl under the same solution phase conditions (Fig. S11†). In particular, no change in the ^1^H NMR spectral features (other than signal broadening) is observed during the titration of **1** with up to about 5 equiv of LiCl. In our experience (involving a wide range of systems), the absence of spectral shifts rules out any particular binding interaction. We thus ascribe the observed broadening to secondary effects, such as salt-induced changes in solvent polarity.

Evidence for a cooperative binding effect involving the Cs^+^ cation and the F^–^ or Cl^–^ anions and receptor **1** was obtained from ^1^H NMR spectroscopic analysis. When receptor **1** was exposed to Cs^+^ as the ClO_4_^–^ salt or to F^–^ and Cl^–^ as the corresponding TBA^+^ salts in CD_3_OD–CDCl_3_ (1/9, v/v), no appreciable chemical shift changes ascribable to ion binding were observed (Fig. S13†). By contrast, but in analogy to what was seen when the ion pair salts CsF and CsCl were used, changes in the proton signals of receptor **1** ascribable to complex formation were seen when exposed to a stoichiometric mixture of CsClO_4_ and TBAF (Fig. S13†). On this basis, we conclude that cooperative effects play a critical role in regulating the ion binding features of **1**. In the case of the Cs^+^, F^–^, and Cl^–^, the ion pairs are complexed effectively, whereas in the absence of a co-bound counterion the individual ions are not appreciably bound, at least in CD_3_OD–CDCl_3_ (1/9, v/v) at the concentrations used for NMR spectroscopic analysis.

**Fig. 9:**
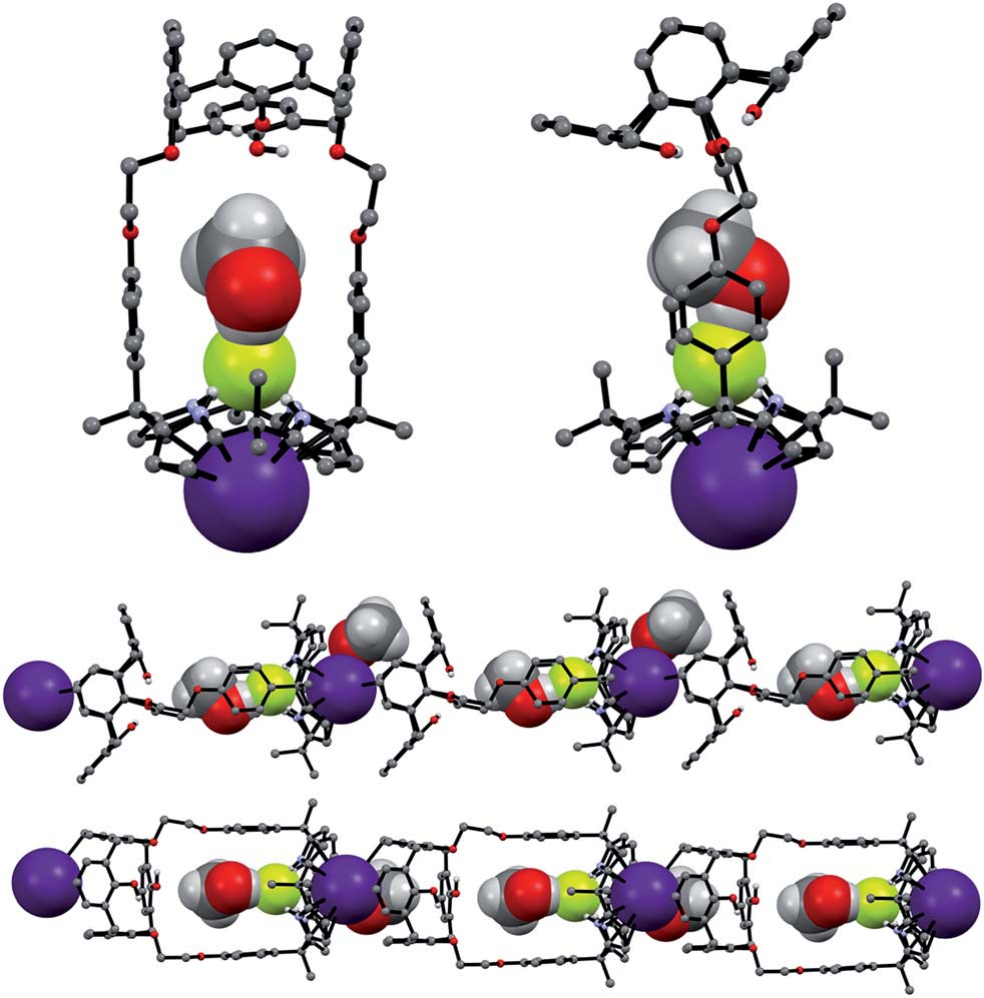
Top: front and side views of the single crystal structure of **1**·CsF·CH_3_OH. Bottom: truncated views of the extended structure seen in the crystal lattice. Most hydrogen atoms and solvent molecules not involved in ion pair complexation have been removed for clarity.

Single crystal X-ray diffraction analyses of the CsF and CsCl complexes of receptor **1** were also carried out. Appropriate single crystals of the CsF complex were obtained by slow evaporation of a mixture of chloroform and methanol containing **1** in the presence of excess cesium fluoride (≈10 equiv). The resulting crystal structure revealed that **1** forms a 1 : 1 cesium fluoride complex (**1**·CsF) in the solid state. In this complex, the Cs^+^ cation is bound to the cone-shaped calix[4]pyrrole cavity formed as the result of fluoride anion binding to the NH protons of the calix[4]pyrrole (Fig. 9). The bound F^–^ anion is hydrogen bonded to a methanol molecule. This latter residual solvation is thought to prevent the Cs^+^ cation from being encapsulated effectively by the Lewis-basic calix[4]arene moiety.

In the solid state, the Cs^+^ cation bound within the calix[4]pyrrole cavity also forms a coordination complex with a different CsF complex of **1** *via* π–metal interactions involving two aromatic rings of the calix[4]arene subunit (Fig. 9). These latter interactions appear to be enhanced by encapsulation of the F^–^ anion between the rigid phenyl spacers. This encapsulation brings the two diagonal aromatic rings of the calix[4]arene closer to one another, thus increasing their ability to interact with the Cs^+^ cation.

**Fig. 10:**
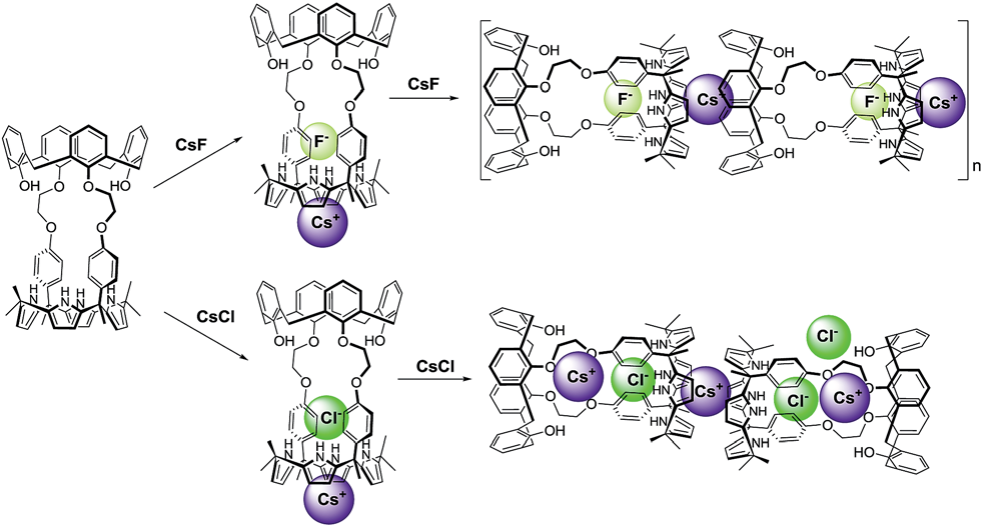
Proposed binding modes associated with the interaction of **1** with CsF and CsCl as inferred from ^1^H NMR spectroscopic studies performed in 10% CD_3_OD–CDCl_3_.

Additional evidence for the π–metal interaction proposed in the case of **1**·CsF came from ^1^H NMR spectroscopic analyses carried out in 10% CD_3_OD–CDCl_3_. Adding additional molar equivalents of CsF to the 1 : 1 CsF complex of **1** (**1**·CsF) causes the aromatic proton resonances of the calix[4]arene to undergo a downfield shift beyond what is observed in the formation of the initial complex (Fig. S14†). This shift is consistent with the Cs^+^ cation bound within the calix[4]pyrrole cavity interacting with the calix[4]arene aromatic rings of a different CsF complex of **1**. This intermolecular interaction is expected to compete with and weaken the π–cation interaction between the Cs^+^ cation and π-electrons of the calix[4]pyrrole cavity, resulting in a downfield shift in the pyrrolic CH proton resonances, as seen by experiment (Fig. S14†).^8^ The net result of this inter-complex interaction is the formation of a coordination polymer stabilized by CsF complexation in solution as well as in the solid state (Fig. 9 and 10).^8^

**Fig. 11:**
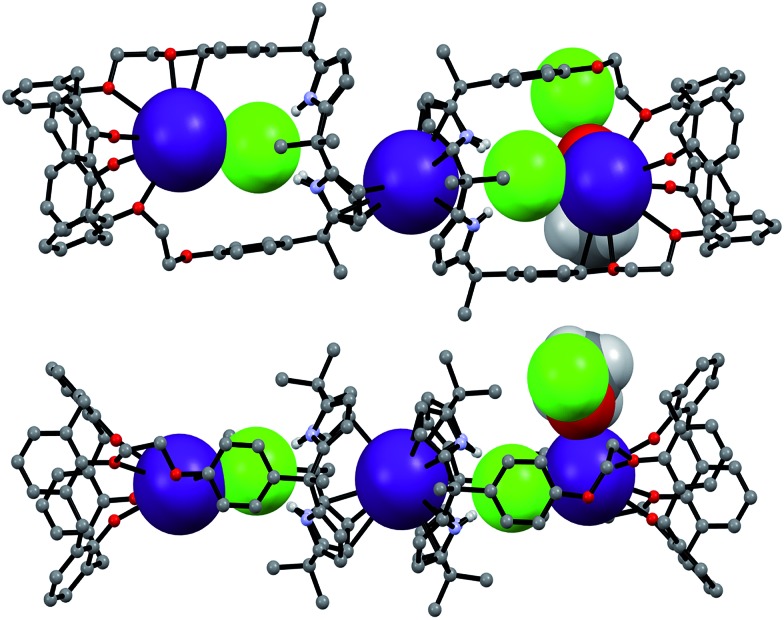
Front and side views of the single crystal structure of **1**_2_·(CsCl)_3_·CH_3_CH_2_OH. Most hydrogen atoms and solvent molecules not involved in ion pair complexation have been removed for clarity.

In marked contrast to what is observed with CsF, the addition of further molar equivalents of CsCl to **1**·CsCl induces no appreciable chemical shift change in the ^1^H NMR spectrum (Fig. S15†). However, it was found that receptor **1** forms an unprecedented 2 : 3 complex with CsCl (*i.e.*, **1**_2_·(CsCl)_3_) in the solid state (Fig. 10 and 11). This conclusion is based on a structural analysis of single crystals obtained by subjecting a chloroform/ethanol solution of **1** to slow evaporation in the presence of excess CsCl. In the resulting multicomponent complex (**1**_2_·(CsCl)_3_), the cesium cations are coordinated by the oxygen atoms of the calix[4]arene, as well as those of the ether linkages. The Cs^+^···O distances range between 3.16 and 3.66 Å (Fig. 11). The Cs^+^ cation interacts with all four oxygen atoms of the cone-shaped calix[4]arene. This is believed to enhance the separation between the aromatic rings of the calix[4]arene subunit, preventing effective π–metal complexation with the Cs^+^ cation as observed in the CsF complex of **1** (*vide supra*). The other cesium cation is sandwiched between two cone-shaped calix[4]pyrrole cavities (Fig. 10 and 11). Two chloride anions are hydrogen-bonded with the NH protons of the calix[4]pyrrole units forming contact ion pairs with the cesium cations bound to the calix[4]arene units. The latter subunits are present in their cone conformations (Fig. 10 and 11). The other chloride anion is located outside receptor **1** and separated from the bound cesium cation by an ethanol molecule (Fig. 11).

Further insights into the binding of CsF and CsCl to receptor **1** under various conditions came from diffusion ordered NMR spectroscopy (DOSY) analyses carried out in 10% CD_3_OD in CDCl_3_. For example, the weight-average diffusion coefficient of the 1 : 1 complex of **1** with CsF (formed by adding 2 molar equiv to a 5.07 mM solution of **1**) was ≈10% higher than the value of the corresponding CsCl complex (Fig. S16 and S17†). This finding is consistent with the proposition that **1** binds CsCl to form a complex with a higher stoichiometry (*e.g.*, 2 : 3, as seen in the solid state; *cf.* Fig. 11) than the 1 : 1 binding stoichiometry characteristic of the CsF complex. When an excess of CsF is added to the initial 1 : 1 complex, the diffusion coefficient of **1** decreases by >60% as compared to the 1 : 1 complex (Fig. S18†). This finding is consistent with what is seen in the solid state, namely formation of a supramolecular polymer that contains the 1 : 1 CsF complex of **1** as a repeat unit.

**Fig. 12:**
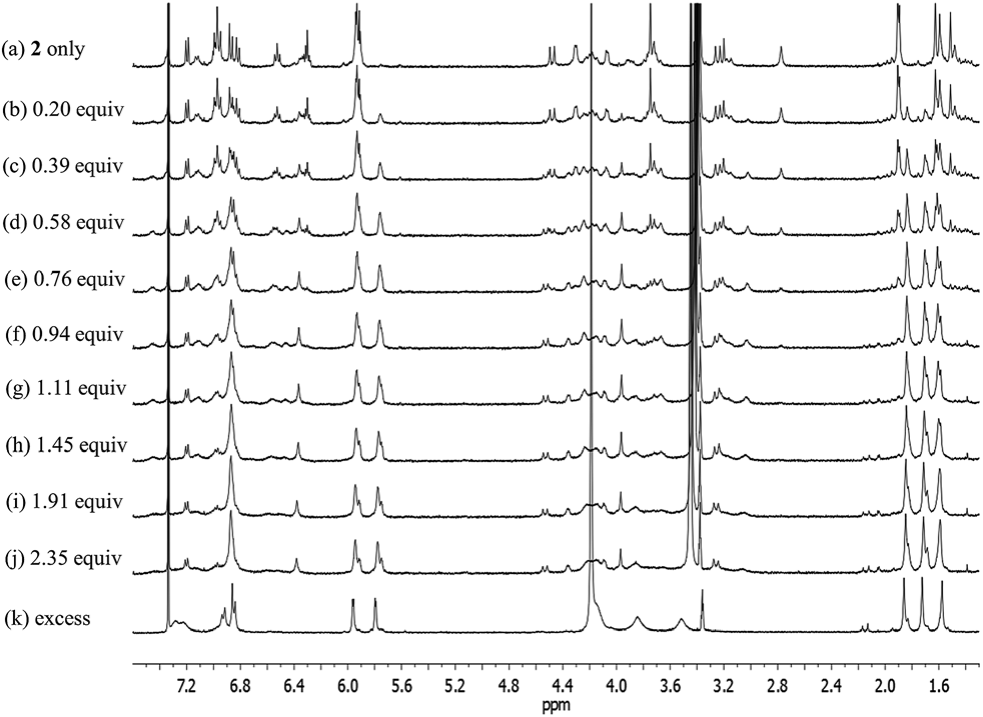
^1^H NMR spectra measured during the titration of receptor **2** with CsF in CD_3_OD–CDCl_3_ (1 : 9, v/v).

Receptor **2** bearing a conformationally flexible calix[4]arene unit was also found to bind CsF and CsCl, but not CsBr or CsNO_3_, under the same solution phase conditions as those described above (Fig. S19†). This binding selectivity mirrors what was seen for **1** (see above), but again stands in contrast to what was observed for **3**, an ion pair receptor that was previously found to interact with all four test salts.^4^ Moreover, the manner whereby **2** interacts with CsF and CsCl differs substantially from what is seen in the case of both **1** and **3**, receptors that contain the calix[4]arene subunits fixed in their cone and 1,3-alternate conformations, respectively.^4^ The ^1^H NMR spectrum of receptor **2** in its ion-free form recorded in 10% CD_3_OD in CDCl_3_ is similar to that recorded in pure CDCl_3_. In both cases, spectral features are seen that are consistent with the presence of two conformers containing the calix[4]arene units in the cone or the partial cone conformation, respectively (Fig. 12a and S19a†).

Upon subjecting **2** to titration with CsF in 10% CD_3_OD–CDCl_3_, two sets of proton signals corresponding to the ion-free form (mixture of conformers; see above) and the CsF complex of **2**, respectively, were seen in the ^1^H NMR spectra recorded before saturation was achieved (Fig. 12). Essentially complete conversion to the complex form was seen upon the addition of 1.11 equiv of CsF. On the basis of these spectral observations, we conclude that receptor **2** forms a relatively strong complex with CsF and that the kinetics of CsF binding and release are slow on the NMR time scale.

During the ^1^H NMR spectroscopic titration of **2** with CsF, the aromatic CH protons associated with both the calix[4]arene and the calix[4]pyrrole constituents shift to higher field (Fig. 12). Such findings are consistent with a binding mode in which the cesium cation is bound to the cone-shaped calix[4]pyrrole cavity with the fluoride anion being hydrogen-bonded to the NH protons of the calix[4]pyrrole. In analogy to what was seen in the ion-free form and in the TBAF complex (see above), the ^1^H NMR spectra of **2** recorded in the presence of 1.11–4.03 equiv of CsF in 10% CD_3_OD–CDCl_3_ are characterized by two sets of proton signals that are presumably derived from two different conformers of the CsF complex, **2**·CsF (Fig. 12). In this case, the sharp set of peaks includes two double peaks appearing at 3.26 and 4.53 ppm in an AB pattern. These spectral features are considered diagnostic of a calix[4]arene in its cone conformation. The broad set of proton signals that are also seen in these ^1^H NMR spectra are attributed to the portion of the CsF complex of **2** that exists with the calix[4]arene subunit in its partial cone conformation. As above, the presence of two distinct sets of signals is consistent with two conformers being present in the presence of ≥1.11 equiv of CsF, whereas the observation of multiple, distinct proton signals prior to saturation is taken as evidence that the binding of CsF by **2** takes place under the conditions of slow equilibrium on the NMR time scale.

**Fig. 13:**
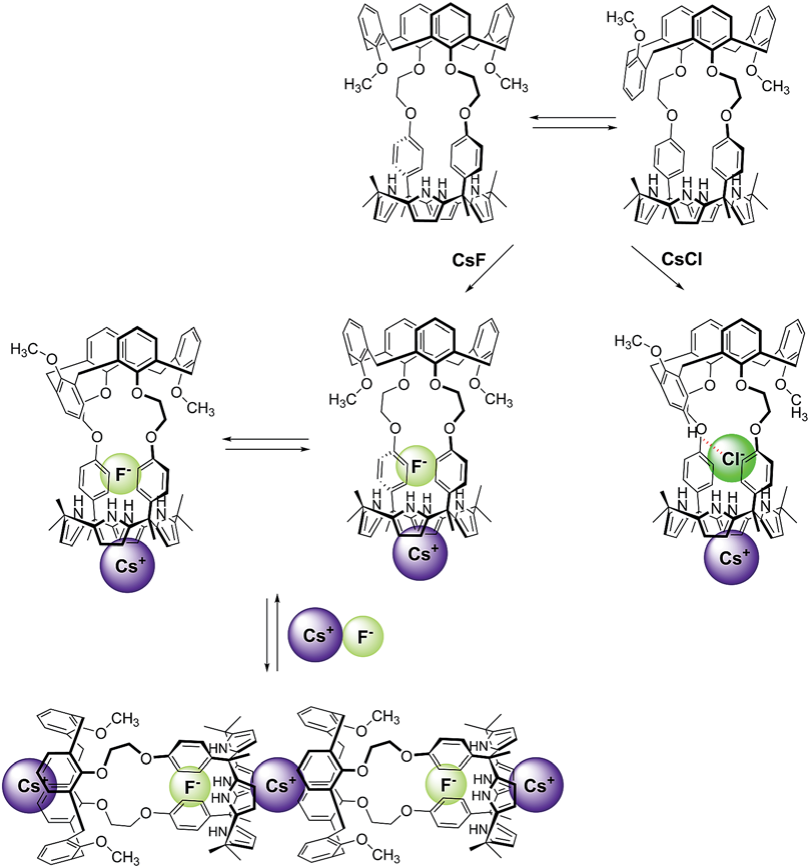
Proposed binding modes of **2** with CsF and CsCl as inferred from ^1^H NMR spectroscopic studies performed in 10% CD_3_OD–CDCl_3_. See main text for discussion.

The further addition of CsF to the 1 : 1 **2**·CsF complex gives rise ultimately to a more simplified ^1^H NMR spectrum (Fig. 12k and S19b†). In this final spectrum, the aromatic protons of the calix[4]arene unit are shifted to lower field and appear as two broad singlet peaks between 7.2 and 7.3 ppm. These features are consistent with π–metal interactions between the aromatic ring of the calix[4]arene and the cesium cation. However, since the formation of the 1 : 1 complex is complete prior to the addition of ≥1.11 equiv of CsF, the observed spectral changes are attributed to inter-complex interactions. Consistent with this suggestion, downfield shifts in the pyrrolic CH proton signals of the calix[4]pyrrole moiety are also seen. Such shifts are expected for a sandwich-like complex wherein, as above, the bound cesium cation interacts in an intermolecular sense with the aromatic rings of a calix[4]arene within a different molecule of **2** (Fig. 12 and 13). This interaction results in formation of a supramolecular ensemble (Fig. 13), as has been recently been observed in the case of naphthocrown-strapped calix[4]pyrroles in the presence of certain ion pair salts.^8^ The simplified nature of the spectrum is consistent with the CsF-mediated inter-complex aggregation and disaggregation process occurring rapidly on the NMR time scale.

In the presence of excess CsF, the aliphatic proton signals of **2** appear as only three peaks in the 3.5–4.2 ppm spectral region. Such a finding is taken as evidence that the calix[4]arene exists in a highly symmetric conformation (Fig. 12k and S19b†). On the other hand, the absence of a clear AB pattern for the calix[4]arene methylene bridge signals leads us to suggest that the calix[4]arene unit adopts a pinched cone conformation, rather than a pure cone conformation (Fig. 12k and S19b†) for which such a diagnostic signal would be expected. Under this condition, two separate sets of peaks appear for the proton signals associated with the calix[4]pyrrole unit in ^1^H NMR spectra of **2** (Fig. 12). In this case, small shoulders present in the proton signals corresponding to the β-pyrrolic CH and the methyl groups of the calix[4]pyrrole unit are presumed to result from the formation of an aggregated species wherein not all pyrrole NH protons interact with the fluoride anion. By contrast, the major peaks, experiencing relatively small upfield shifts, are ascribed to a classic anion bound calix[4]pyrrole species wherein the macrocycle exists in the cone conformation and all four pyrrolic NHs interact with the fluoride anion. Although reflecting measurements carried out under very different experimental conditions, the results of the X-ray structural studies (*vide infra*) are completely consistent with what was inferred on the basis of the ^1^H NMR spectroscopic analyses.

**Fig. 14:**
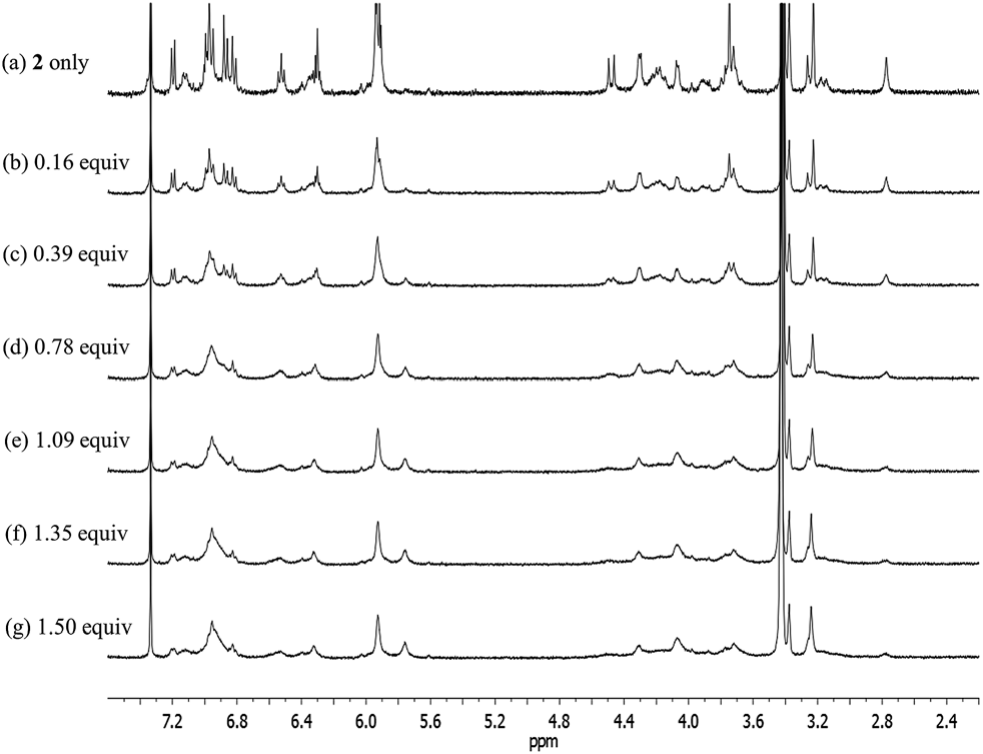
^1^H NMR spectra for the titration of receptor **2** with CsCl in CD_3_OD–CDCl_3_ (1 : 9, v/v).

Significantly different changes in the ^1^H NMR spectra (CD_3_OD–CDCl_3_; 1/9, v/v) were seen when receptor **2** was exposed to CsCl, rather than CsF (Fig. 14). For instance, when **2** was subjected to titration with CsCl, all of the proton signals in the ^1^H NMR spectrum associated with the cone conformation of the calix[4]arene constituent, including the two doublets for the methylene protons of the calix[4]arene, gradually disappear before saturation is reached at 1.35 equiv of CsCl (Fig. 14). After saturation, the ^1^H NMR spectra were consistent with receptor **2** being in a form wherein the calix[4]arene unit exists only the partial cone conformation. This leads us to propose that the binding of CsCl to receptor **2** induces a conversion to a single conformer and that the binding of CsCl locks the calix[4]arene unit into the partial cone conformation (Fig. 13). As will be discussed below, this locking is attributed to a CH–Cl hydrogen bond interaction with a phenoxy group that stabilizes the partial cone configuration.

The structural locking of the calix[4]arene unit of **2** into a partial cone conformation as the result of CsCl binding is presumed to preclude intermolecular π–metal interactions between the calix[4]pyrrole-bound cesium cation and the aromatic rings of an adjacent calix[4]arene. Indeed, no evidence of supramolecular polymer is found in the ^1^H NMR spectrum. This stands in contrast to what is seen in the case of the CsF complexes of both **1** and **2** (Fig. 12 and S20†).

**Fig. 15:**
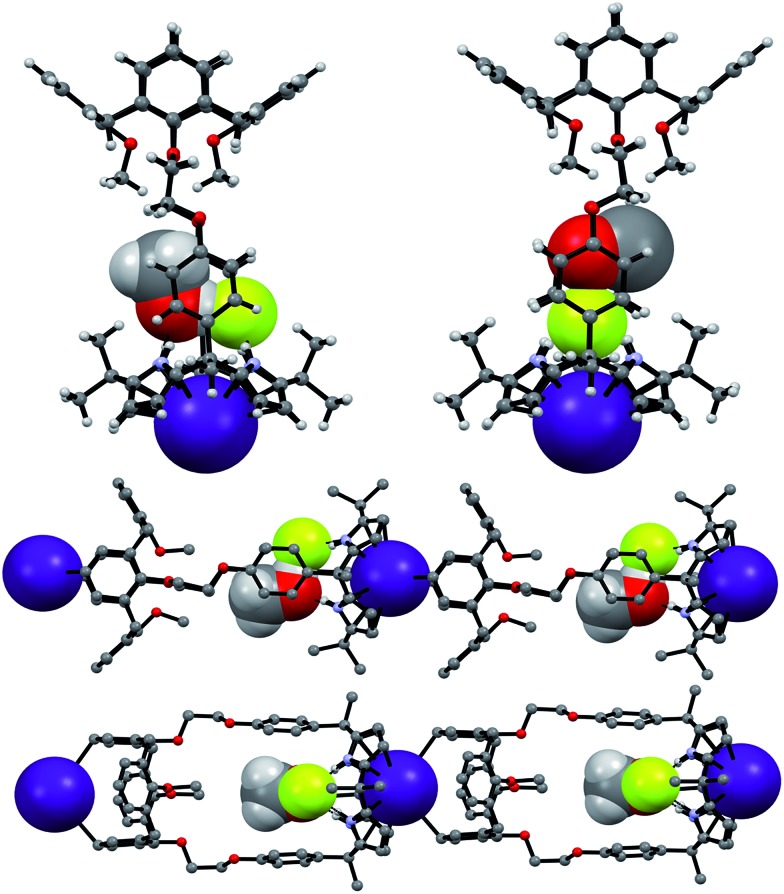
Top: views of the two different binding modes seen in single crystals of **2**·CsF·CH_3_OH. Bottom: partial view of the extended structure found in the crystal lattice. Most hydrogen atoms and solvent molecules not involved in ion pair complexation have been omitted for clarity.

The CsF and CsCl complexes of **2** were analyzed in the solid state *via* X-ray diffraction analysis. Suitable single crystals were obtained by slow evaporation of a chloroform/methanol solution of **2** in the presence of excess CsF and CsCl, respectively. The crystal structure of the CsF complex revealed that the calix[4]arene subunit in **2** exists in the pinched cone conformation (Fig. 15). The fluoride anion is bound to the NH protons of calix[4]pyrrole present in its cone conformation, while the cesium cation is complexed with the calix[4]pyrrole cavity *via* presumed π–cation interactions. The cesium cation also interacts with the two phenoxy groups of the calix[4]arene of a different molecule of **2** (Fig. 15).

This solid state analysis provides support for the inference drawn from the ^1^H NMR spectroscopic analyses carried out in 10% CD_3_OD–CDCl_3_ solution, namely that two different kinds of binding interactions involving receptor **2** and the F^–^ anion are stabilized in the presence of excess CsF (*cf.* Fig. 12 and S19b†). In one mode, the fluoride anion is hydrogen bonded to two pyrrolic NH protons of the calix[4]pyrrole and solvated by a methanol molecule that interacts with the remaining two pyrrolic NH protons *via* hydrogen bonding (Fig. 15). In the other binding mode, the fluoride anion is symmetrically bound to all four NH protons of the calix[4]pyrrole and also hydrogen bonded to a methanol molecule positioned at its top (Fig. 15). Based on the single crystal X-ray diffraction analysis, these two binding modes coexist in the ratio of 78 to 22% in the solid state with the first binding mode being dominant.

**Fig. 16:**
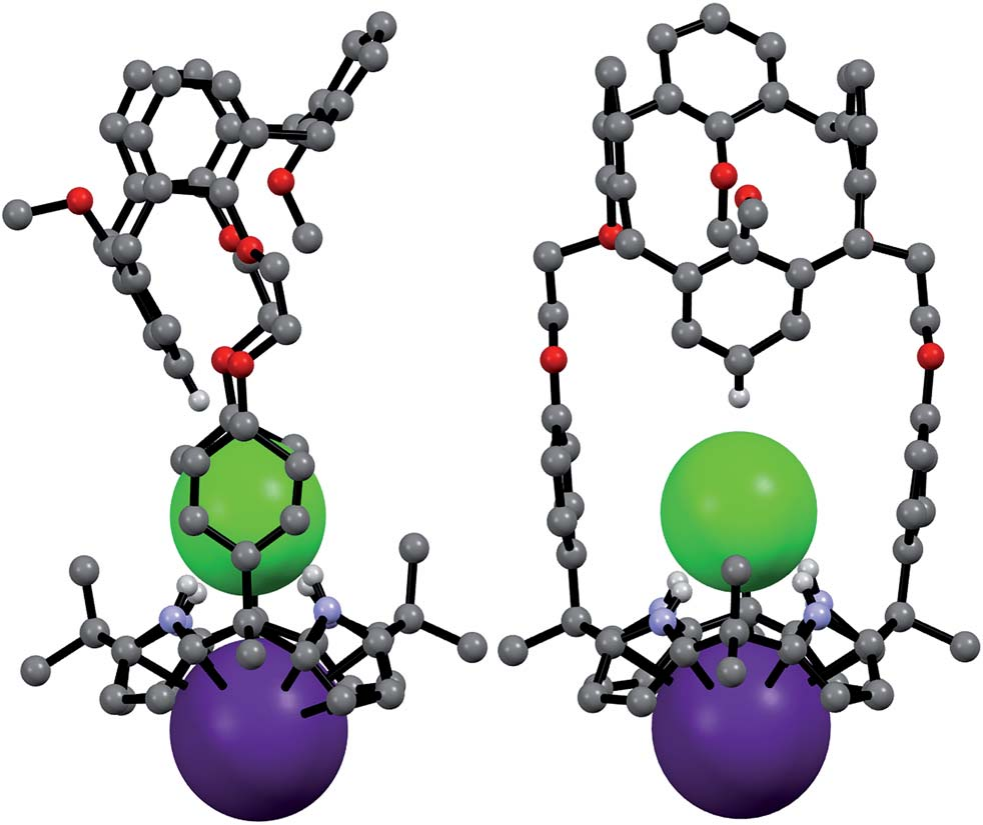
Front and side views of the single crystal structure of **2**·CsCl. Most hydrogen atoms and solvent molecules not involved in ion pair complexation have been removed for clarity.

Structural analysis of the CsCl complex reveals that in the solid state the calix[4]arene unit in **2** adopts a partial cone conformation, while the calix[4]pyrrole unit is fixed in the cone conformation. The chloride anion is bound to the pyrrolic NH protons *via* hydrogen bonding, while the cesium cation is encapsulated by the cone-shape calix[4]pyrrole cavity (Fig. 16). In contrast to what is true for the corresponding CsF complex (*vide supra*), no solvation of the chloride anion by methanol was observed. Such findings are in accord with what was inferred in solution based on the ^1^H NMR spectroscopic analyses discussed above (Fig. 13 and 14).

A more detailed analysis of the structure of **2**·CsCl revealed that the proton *para* to the methoxy group of the inverted aromatic ring of the calix[4]arene interacts with the chloride anion *via* a presumed aromatic CH–anion hydrogen bond (Fig. 16). The distance for this interaction, 3.64 Å (C···Cl^–^), is consistent with the presence of a CH–Cl hydrogen bond in that it lies in the range of C···Cl^–^ distances obtained from electronic structure calculations, namely 3.38–3.50 Å.^9^ This experimental distance is also close to the mean value observed for aryl CH–Cl hydrogen bonding interactions in other crystal structures, *i.e.*, 3.81 ± 0.15 Å.^10^ In addition, the C–H···Cl angle, 171°, is nearly linear, as would be expected for a strong CH–anion interaction.

Based on prior calculations, hydrogen bonds between aryl CH groups and monovalent anions are characterized by interaction energies ranging from –6 to –27 kcal mol^–1^ depending on the nature of the arene.^9,10^ For comparison, the pyrrole NH group binds Cl^–^ with an interaction energy of –22.5 kcal mol^–1^.^11^ In the case of **2**·CsCl, RI-MP2/aug-cc-pVDZ calculations on the model arene, 1,3-methyl-2-methoxybenzene, lead to the prediction that the CH–Cl interaction should provide –8.3 kcal mol^–1^ of stabilization energy. Thus, by adopting the partial cone configuration, **2** positions a moderate hydrogen bond donor group (a calix[4]arene-derived aryl CH group) for interaction with the bound Cl^–^ anion. This interaction stabilizes the calix[4]arene partial cone conformation to an extent that the cone conformation is no longer significantly populated. Such stabilization does not occur with F^–^. Likely this reflects the fact that, due to its smaller size, the latter anion lies significantly closer to the calix[4]pyrrole core and thus at a greater distance from the inverted phenoxy group. A molecular mechanics optimized geometry of the analogous conformation of **2** with CsF yields a C···F distance of 4.46 Å. This value is consistent with the absence of any significant CH–F interaction.

Taken together, the binding behavior of **2** towards the two test cesium salts of this study differs significantly from that of **3** whose calix[4]arene unit is fixed in the 1,3-alternate conformation. For example, **3** is able to form ion pair complexes with CsF, CsCl, CsBr, and CsNO_3_, where the cesium cations are bound to the ether linkages with the aid of π–cation interaction with the inverted aromatic rings of the calix[4]arene. In contrast, under the same conditions of analysis, receptor **2** was found to bind only CsF and CsCl. In the resulting ion pair complexes the cesium cations are coordinated within the cone-shape calix[4]pyrrole cavity with the anions being hydrogen bonded to the pyrrolic NH protons. Such findings are consistent with the suggestion that the energy for conformational conversion of the calix[4]arene subunit of **2** into the corresponding 1,3-alternate conformation is too high to be offset by the stabilization energy associated with binding the cesium cation to either the calix[4]arene moiety or the tethering ether linkages.

## Conclusions

1,3-Dihydroxycalix[4]arene- and 1,3-dimethoxycalix[4]arene-strapped calix[4]pyrroles (**1** and **2**) have been synthesized and their complexation chemistry with anions and ion pairs studied. ^1^H NMR spectroscopic studies and single crystal X-ray diffraction analyses revealed that the calix[4]arene unit of **1** is locked in the cone conformation as a result of intra-molecular hydrogen bonding, while that of **2** is conformationally mobile and interconverts between the cone and the partial cone conformations in solution. Both **1** and **2** were found to bind the fluoride and the chloride anions with the nature of the interactions depending on the choice of counter cation. For example, these receptors bind only the fluoride anion when studied in the form of the less coordinating tetrabutylammonium cation salts. However, both the fluoride and chloride anions are bound by **1** and **2** when studied as their cesium cation salts. In contrast to the control system **3**, neither receptor **1** nor receptor **2** interacts appreciably with CsBr or CsNO_3_ in 10% CD_3_OD–CDCl_3_.

The nature of the CsF and CsCl complex varies with the receptor and the conditions. Receptor **1** binds CsF and CsCl *via* two different binding modes both in solution and in the solid state. In the presence of *ca.* 1 molar equiv of CsF, it forms a 1 : 1 complex wherein the cesium cation is contained within the cone-shape calix[4]pyrrole cavity and the anion is hydrogen bonded to the NHs of the calix[4]pyrrole. The addition of further CsF to this 1 : 1 complex results in formation of a supramolecular polymer. In contrast, CsCl is complexed by **1** with a 2 : 3 (**1** : CsCl) binding stoichiometry in the solid state. No evidence of further aggregation is seen in the presence of excess CsCl.

In the case of receptor **2**, the calix[4]arene unit remains conformationally mobile and interconverts between the cone and the partial cone conformation on the NMR time scale after forming a 1 : 1 CsF complex. In the presence of excess CsF, the calix[4]arene constituent of **2** exists in the form of a single pinched cone conformer. Under these latter conditions, the complexed cesium cation is bound to the aromatic rings of a separate calix[4]arene *via* π–cation interactions. The net result is formation of a supramolecular polymer in the solid state. In the presence of CsCl, the calix[4]arene unit of **2** exists only in the partial cone conformation. This conformation is stabilized in part by an aryl CH–chloride anion hydrogen bond involving the aromatic proton of a single calix[4]arene phenoxy group.

The binding behavior of **1** and **2**, both in solution and in the solid state, stands in sharp contrast to what was seen with **3**, a control system wherein the calix[4]arene subunit remains fixed in a 1,3-alternate conformation. Receptor **3** displays considerably reduced selectivity in terms of ion pair recognition than either **1** or **2**. For instance, when studied under identical solution phase conditions, receptor **3** binds a range of cesium anion salts, whereas **1** and **2** only interact appreciably with CsF and CsCl.

On the basis of the findings presented here, we suggest that control of calix[4]arene conformation represents a new approach to modulating the inherent cation and anion recognition properties of ostensibly similar ion pair receptors. It thus provides an attractive design consideration that could be exploited to create even more selective receptor systems tuned to interact with specific anions, cations, or their corresponding ion pairs.
