# Supplementary material for: Ion pair-induced conformational motion in calix[4]arene-strapped calix[4]pyrroles
Source: Chem Sci. 2014 Dec 3;6(2):1404–13. doi: 10.1039/c4sc03272a (PMC5811093; doi:10.1039/c4sc03272a)
Supplement: Supplementary file 1 [file SC-006-C4SC03272A-s001.pdf]

Electronic Supplementary Material (ESI) for Chemical Science.
This journal is © The Royal Society of Chemistry 2014

Supporting Information

For

Ion Pair-Induced Conformational Motion in Calix[4]arene-Strapped Calix[4]pyrroles

Sung Kuk Kim,[a] Vincent M. Lynch,[a] Benjamin P. Hay,[b] Jong Seung Kim,[*c] and Jonathan L. Sessler[*a]

[a]*Department of Chemistry, The University of Texas at Austin, 105 E. 24th, Street-Stop A5300, Austin, Texas 78712-1224,* [b]*Supramolecular Design Institute, Oak Ridge, TN 37830-7185, and* [c]*Department of Chemistry, Korea University, Seoul 136-701, Korea*

∗ Corresponding Author: sessler@cm.utexas.edu

Scheme S1. Synthesis of receptors **1** and **2**

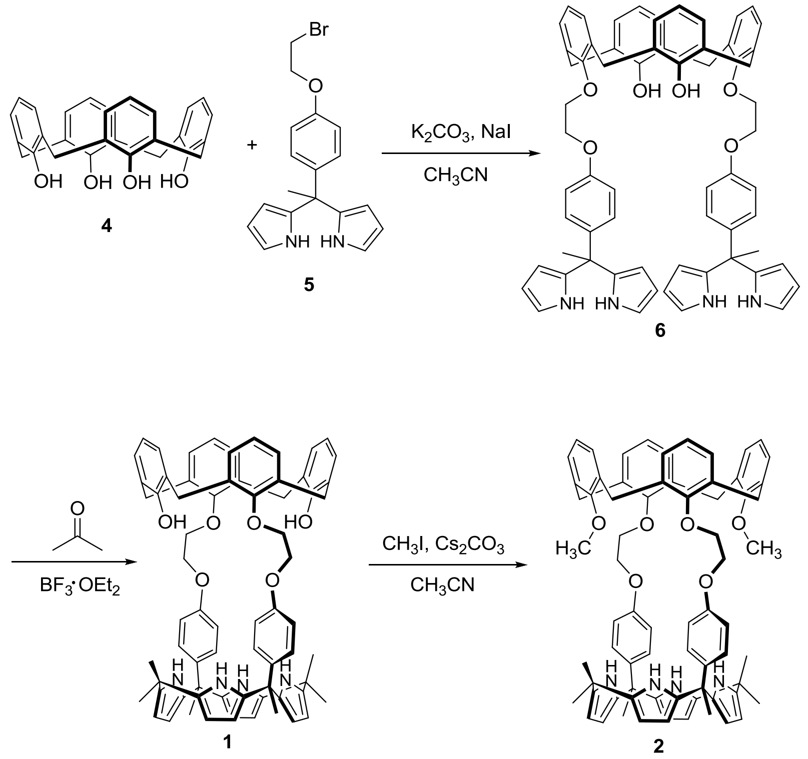

All solvents and chemicals used were purchased from Aldrich, TCI, and Acros and used without further purification. Compound **5** was prepared as reported previously.[1] NMR spectra were recorded on a Varian Mercury 400 instrument. The NMR spectra were referenced to solvent and the spectroscopic solvents were purchased from either Cambridge Isotope Laboratories or Aldrich. Chemical ionization (CI) and electrospray ionization (ESI) mass spectra were recorded on a VG ZAB-2E instrument and a VG AutoSpec apparatus, respectively. TLC analyses were carried out using Sorbent Technologies silica gel (200 mm) sheets. Column chromatography was performed on Sorbent silica gel 60 (40–63 mm).

Compound 6: Calix[4]arene **4** (1.50 g, 3.53 mmol), dipyromethane **5**[1] (2.67 g, 7.43 mmol), sodium iodide (0.10 g, 0.67 mmol) and K_2CO_3 (0.59 g, 4.26 mmol) were dissolved in acetonitrile (100 mL) under a nitrogen atmosphere. After the resulting reaction mixture was heated at reflux for 8 days and allowed to cool to RT, the acetonitrile was removed *in vacuo*. To the resulting sticky solid, CH_2Cl_2 (100 mL) and water (100 mL) were added. The organic layer was separated off and washed three times with 100 mL of water. The organic layer was dried over anhydrous $MgSO_4$ and the solvent was evaporated *in vacuo* to give a yellowish sticky solid. Column chromatography over silica gel (eluent: ethyl acetate/hexanes (1/4)) gave 1.70 g (49% yield) of **6** as a white solid. ^{1}H NMR (400 MHz, $CDCl_3$): δ 7.90 (s, 2H, ArO*H*), 7.72 (broad s, 4H, N*H*), 7.04 (d, 4H, Ar*H*, *J* = 7.61 Hz), 7.01 (d, 4H, Ar*H*, *J* = 8.81 Hz), 6.92 (d, 4H, Ar*H*, *J* = 7.61 Hz), 6.83 (d, 4H, Ar*H*, *J* = 8.81 Hz), 6.76 (t, 2H, Ar*H*, *J* = 7.61 Hz), 6.64 (t, 2H, Ar*H*, *J* = 7.61 Hz), 6.61 (m, 4H, pyrrole-*H*), 6.15 (m, 4H, pyrrole-*H*), 5.94 (m, 4H, pyrrole-*H*), 4.38 (d, 4H, ArCH_2Ar, *J* = 13.2 Hz), 4.29 (broad s, 4H, OCH_2CH$_2$O), 4.26 (broad s, 4H, OCH_2CH$_2$O), 3.36 (d, 4H, ArCH_2Ar, *J* = 12.8 Hz), 2.00 (s, 6H, ArC(pyrrole)$_2$CH_3) ppm. ^{13}C NMR (100 MHz, $CDCl_3$): δ 157.4, 153.4, 152.0, 140.2, 138.0, 133.8, 129.3, 128.8, 128.7, 128.4, 125.8, 119.3,

117.1, 114.8, 108.4, 106.4, 74.4, 67.0, 44.4, 31.5, 29.3 ppm. HRMS (ESI) *m/z* 1003.44051 $[M + Na]^+$ calcd for $C_{64}H_{60}N_4NaO_6$, found 1003.44003.

1,3-Dihydroxycalix[4]arene-strapped calix[4]pyrrole 1: To compound **5** (3.00 g, 3.06 mmol) in acetone (600 mL) was added BF_3•OEt_2 (2 ml, 18.0 mmol). The resulting solution was stirred for 1 hour at room temperature and then quenched via the addition of triethylamine (5 ml). Evaporation of the volatile components *in vacuo* afforded a yellowish sticky solid. To this crude product, dichloromethane (200 ml), water (200 ml), and triethylamine (5 ml) were added and the organic phase was separated off and washed three times with water (200 ml). The organic layer was dried over anhydrous $MgSO_4$ and evaporated *in vacuo* to give a yellowish solid. Column chromatography over silica gel (eluent: ethyl acetate/hexanes (1/3)), followed by recrystallization from a mixture of dichloromethane and methanol (1/19), gave 0.65 g (20% yield) of **1** as a white solid. ^{1}H NMR (400 MHz, $CDCl_3$): δ 8.15 (s, 2H, ArO*H*), 7.05 (d, 4H, Ar*H*, *J* = 7.61 Hz), 6.99 (d, 4H, Ar*H*, *J* = 7.61 Hz), 6.98 (s, 8H, Ar*H*), 6.81 (t, 2H, Ar*H*, *J* = 7.61 Hz), 6.79 (broad s, 4H, N*H*), 6.64 (t, 2H, Ar*H*, *J* = 7.61 Hz), 6.01 (t, 4H, pyrrole-*H*, *J* = 2.80 Hz), 5.93 (t, 4H, pyrrole-*H*, *J* = 2.80 Hz), 4.68 (t, 4H, OCH_2CH_2O, *J* = 6.00 & 7.20 Hz), 4.44 (t, 4H, OCH_2CH_2O, *J* = 7.20 & 6.00 Hz), 4.35 (d, 4H, $ArCH_2Ar$, *J* = 13.2 Hz), 3.42 (d, 4H, $ArCH_2Ar$, *J* = 12.4 Hz), 1.97 (s, 6H, $ArC(pyrrole)_2CH_3$), 1.49 (s, 6H, $C(CH_3)_2$), 1.41 (s, 6H, $C(CH_3)_2$) ppm. ^{13}C NMR (100 MHz, $CDCl_3$): δ 157.3, 153.2, 151.8, 139.7, 136.9, 133.8, 129.4, 128.7, 128.3, 126.1, 120.0, 114.5, 105.2, 105.2, 73.9, 66.1, 44.6, 35.9, 31.7, 30.3, 30.1, 29.6 ppm. HRMS (ESI) *m/z* 1083.50310 $[M + Na]^+$ calcd for $C_{70}H_{68}N_4NaO_6$, found 1083.50400. This compound was further characterized via a single crystal X-ray diffraction analysis.

1,3-Dimethoxycalix[4]arene-strapped calix[4]pyrrole 2: 1,3-Dihydroxycalix[4]arene-strapped calix[4]pyrrole **1** (0.15 g, 0.14 mmol), iodomethane (excess), and Cs_2CO_3 (0.14 g, 0.43 mmol)

were mixed in acetonitrile (50 mL) under a nitrogen atmosphere. After the resulting reaction mixture was heated at reflux for 12 h and allowed to cool to RT, the acetonitrile was removed *in vacuo*. To the resulting white solid, CH_2Cl_2 (100 mL) and water (100 mL) were added. The organic layer was separated off and washed three times with 100 mL of water. The organic layer was dried over anhydrous $MgSO_4$ and the solvent was evaporated *in vacuo* to give a white solid. Recrystallization from a mixture of dichloromethane and methanol (1/19), following column chromatography over silica gel (eluent: ethyl acetate/hexanes (1/6)), afforded 0.10 g (65% yield) of **2** as a white solid. ^{1}H NMR (400 MHz, DMSO-d_6 at 100 °C): δ 9.26 (broad s, 4H, N*H*), 7.22 (d, 4H, Ar*H*, *J* = 6.40 Hz), 7.01 (d, 4H, Ar*H*, *J* = 7.20 Hz), 6.82 (d, 4H, Ar*H*, *J* = 6.80 Hz), 6.76 (t, 2H, Ar*H*, *J* = 5.60 Hz), 6.61 (broad d, 4H, Ar*H*, *J* = 5.60 Hz), 6.47 (t, 2H, Ar*H*, *J* = 6.00 Hz), 5.86 (broad t, 4H, pyrrole-*H*), 5.70 (broad t, 4H, pyrrole-*H*), 4.29 (broad t, 4H, OCH_2CH_2O), 4.00 (broad s, 4H, OCH_2CH_2O; 4H, $ArCH_2Ar$), 3.43 (d, 4H, $ArCH_2Ar$, *J* = 10.4 Hz), 3.26 (broad s, 6H, $ArOCH_3$), 1.77 (s, 6H, ArC(pyrrole)$_2$CH_3), 1.69 (s, 6H, $C(CH_3)_2$), 1.47 (s, 6H, $C(CH_3)_2$) ppm. ^{13}C NMR (100 MHz, $CDCl_3$): δ 159.0, 157.9, 155.7, 139.6, 137.5, 137.1, 136.9, 133.6, 129.2, 128.9, 128.6, 128.6, 127.9, 122.9, 115.3, 105.1, 104.9, 104.6, 75.9, 73.1, 69.3, 44.3, 35.7, 31.2, 30.1, 29.0 ppm. HRMS (ESI) *m/z* 1111.53440 $[M + Na]^+$ calcd for $C_{72}H_{72}N_4NaO_6$, found 1111.53480. This compound was further characterized via a single crystal X-ray diffraction analysis.

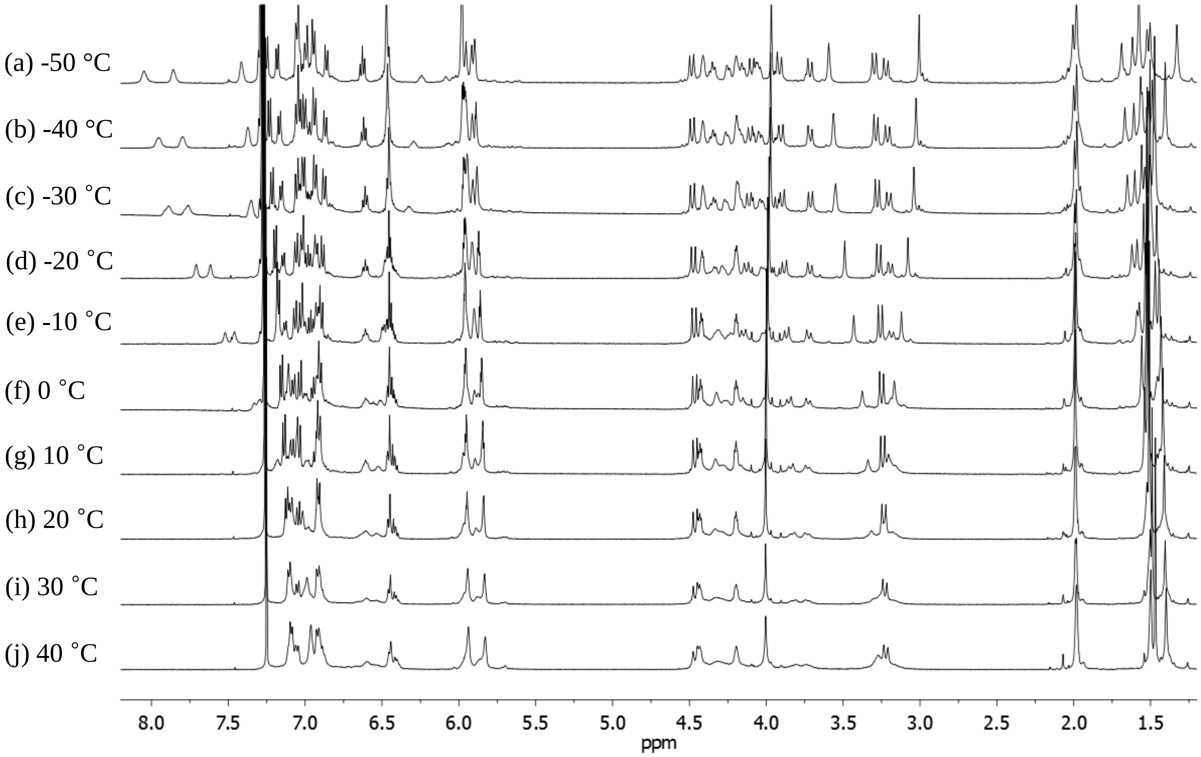

Figure S1. Temperature-dependent ^{1}H NMR spectra of **2** recorded in $CDCl_3$.

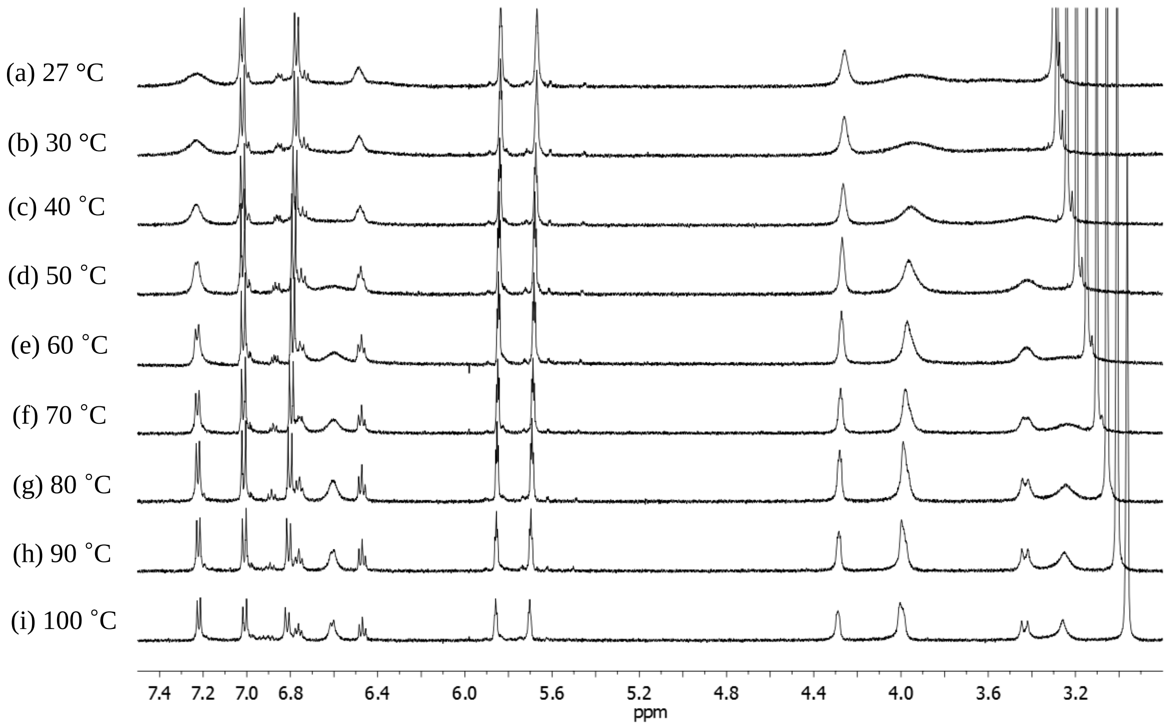

Figure S2. Temperature-dependent ^{1}H NMR spectra of **2** recorded in DMSO.

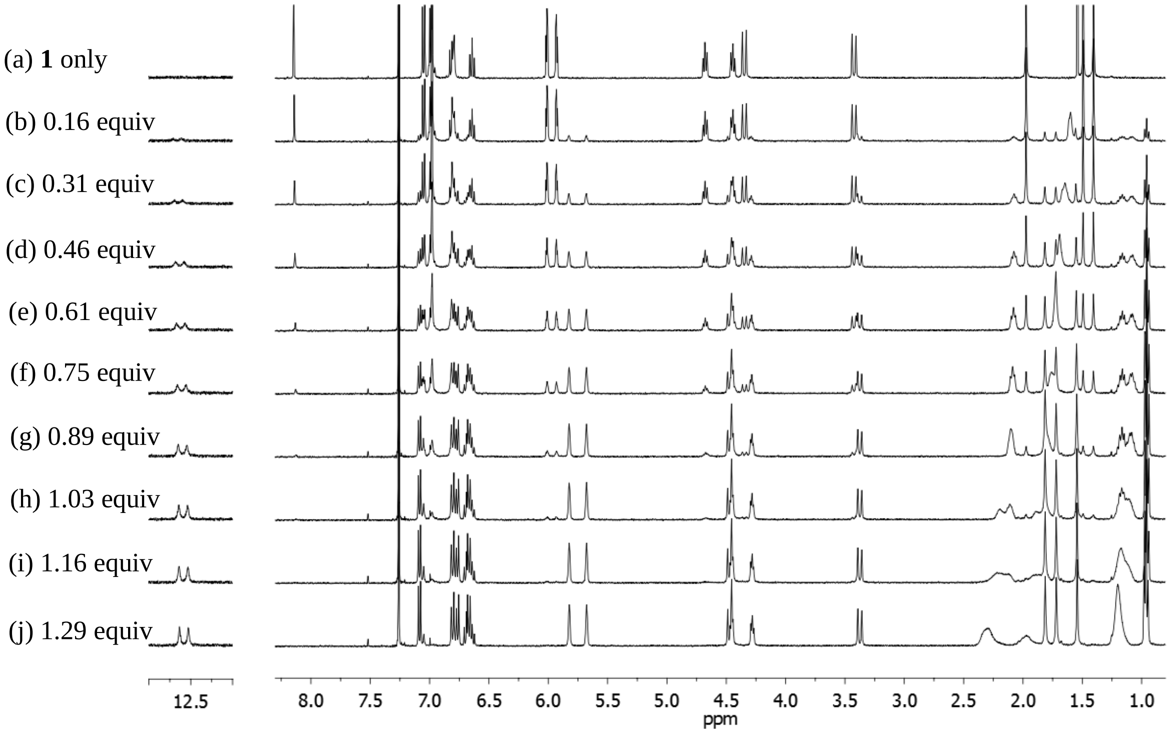

Figure S3. ^{1}H NMR spectra recorded during the titration of receptor **1** with TBAF (tetrabutylammonium fluoride) in $CDCl_3$.

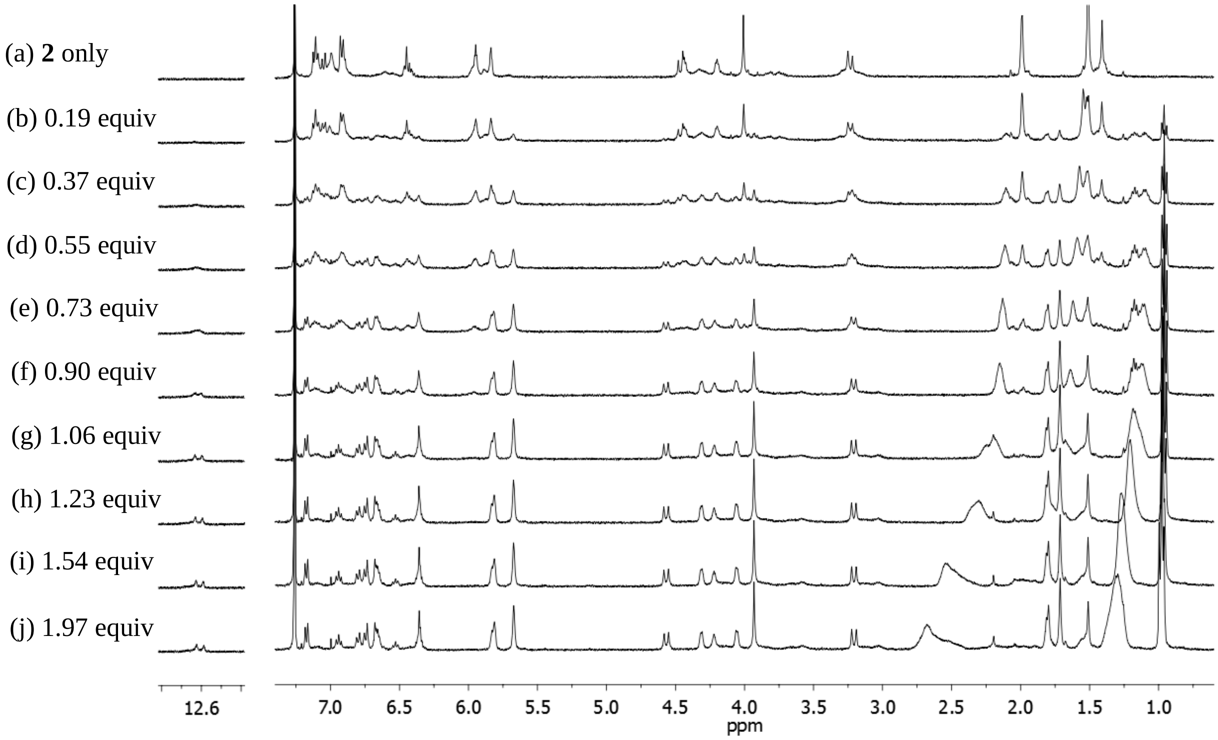

Figure S4. ^{1}H NMR spectra recorded during the titration of receptor **2** with TBAF (tetrabutylammonium fluoride) in $CDCl_3$.

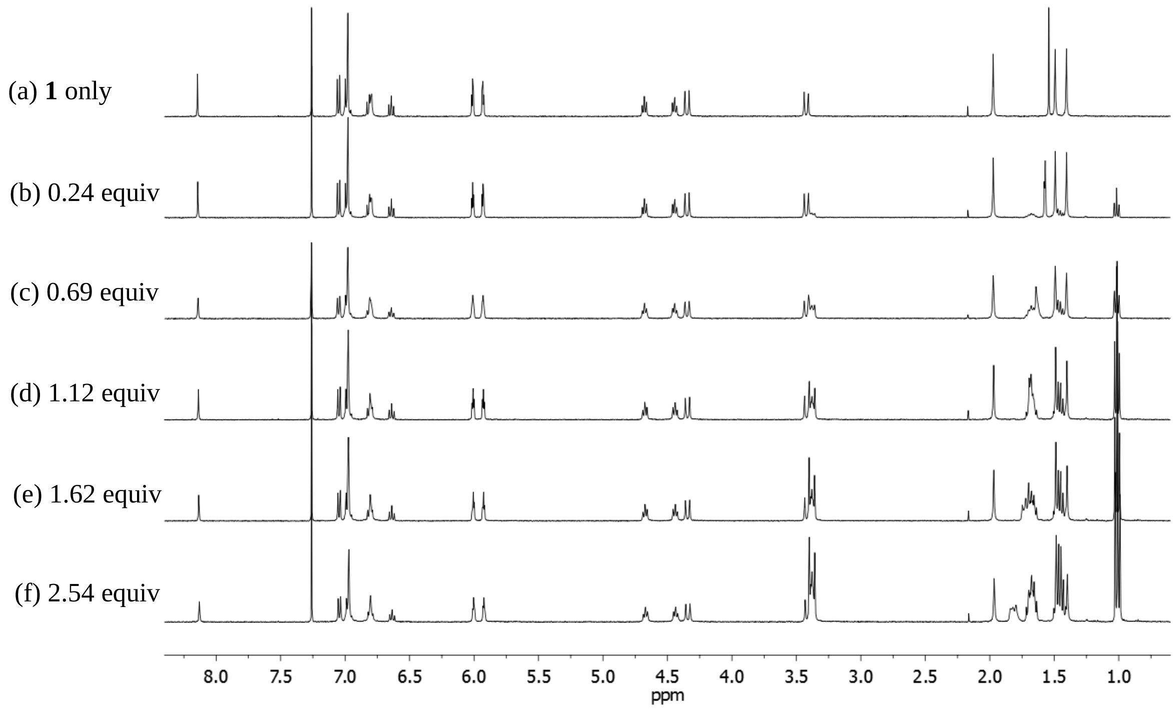

Figure S5. ^{1}H NMR spectra recorded during the titration of receptor **1** with TBACl (tetrabutylammonium fluoride) in $CDCl_3$.

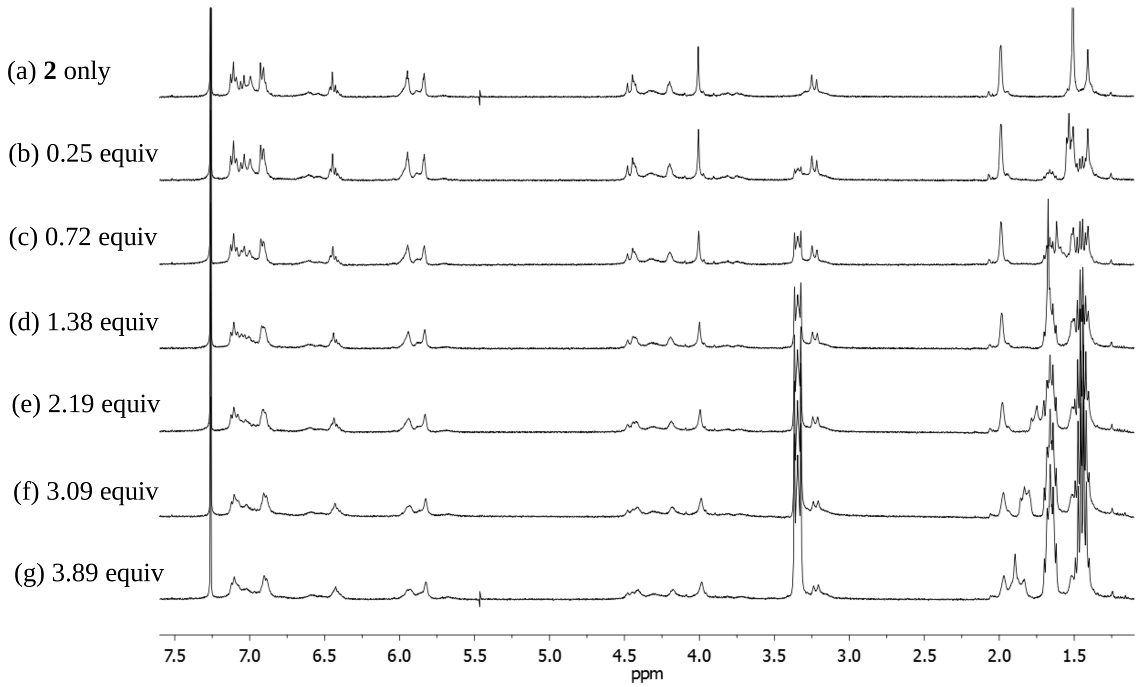

Figure S6. ^{1}H NMR spectra recorded during the titration of receptor **2** with TBACl in $CDCl_3$.

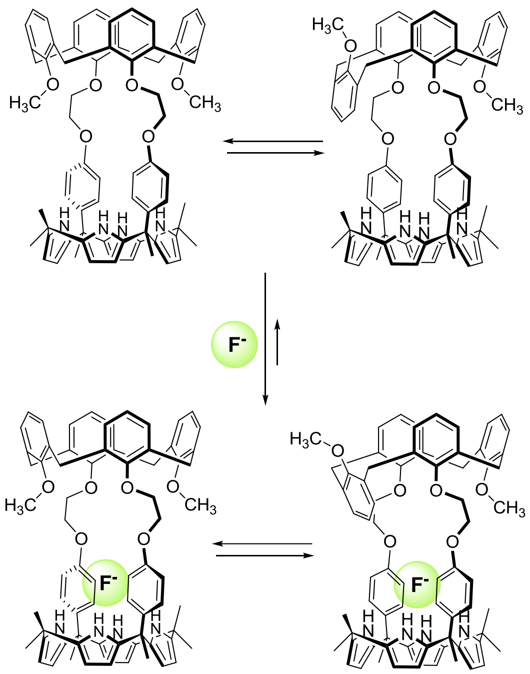

Figure S7. Putative binding modes of **2** with the F^- anion as inferred from ^1H NMR spectroscopic analyses carried out in 10% CD_3OD-$CDCl_3$.

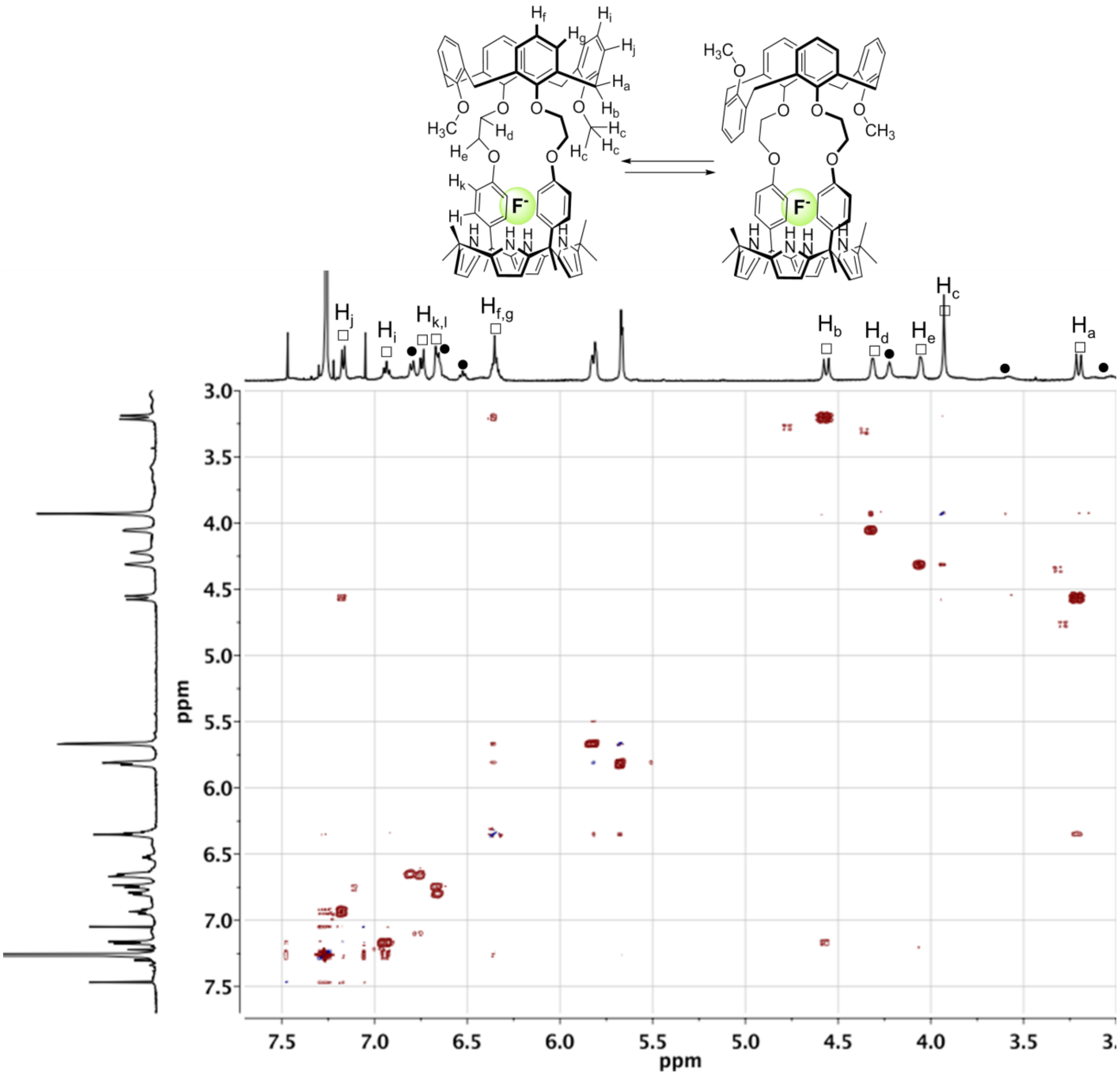

Figure S8. COSY NMR spectrum of compound **2** recorded in the presence of 1.97 equiv of TBAF in $CDCl_3$ (peak designations: □ = cone; ● = partial cone).

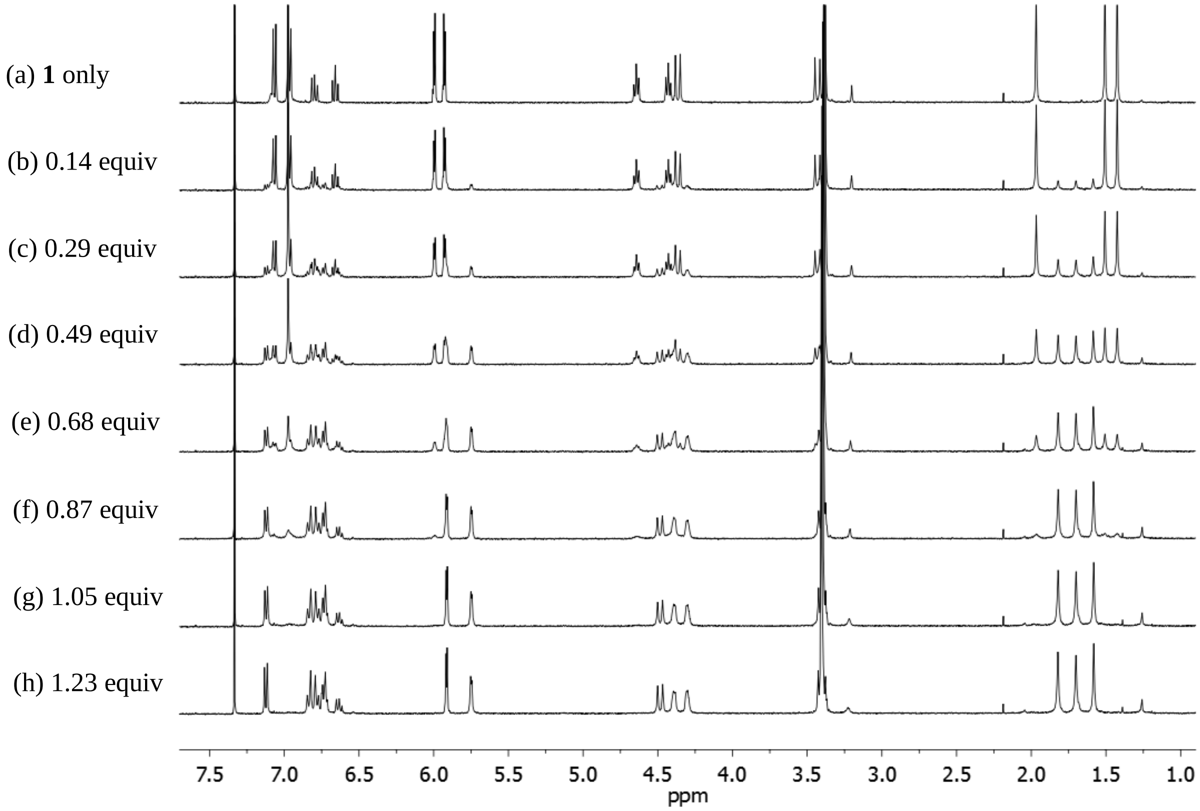

Figure S9. ^{1}H NMR spectra recorded during the titration of receptor **1** with CsF in CD_3OD-$CDCl_3$ (1:9, v/v).

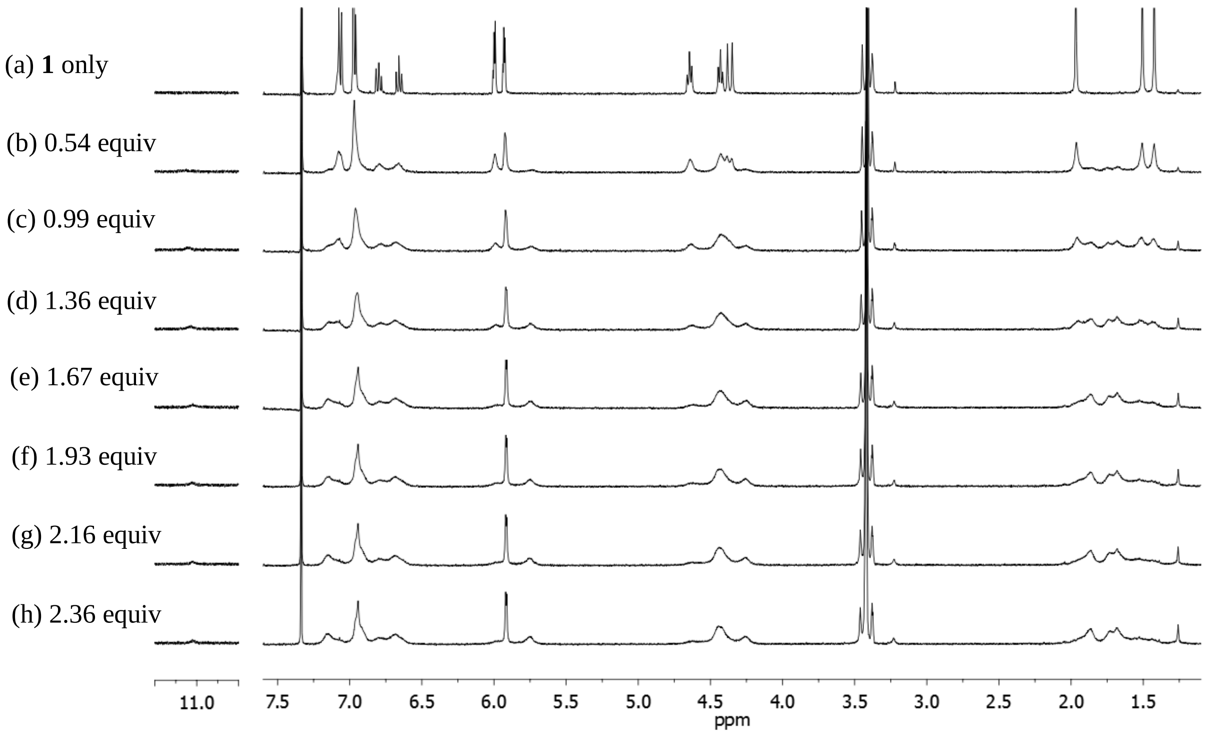

Figure S10. ^1H NMR spectra recorded during the titration of receptor **1** with CsCl in CD_3OD-$CDCl_3$ (1:9, v/v).

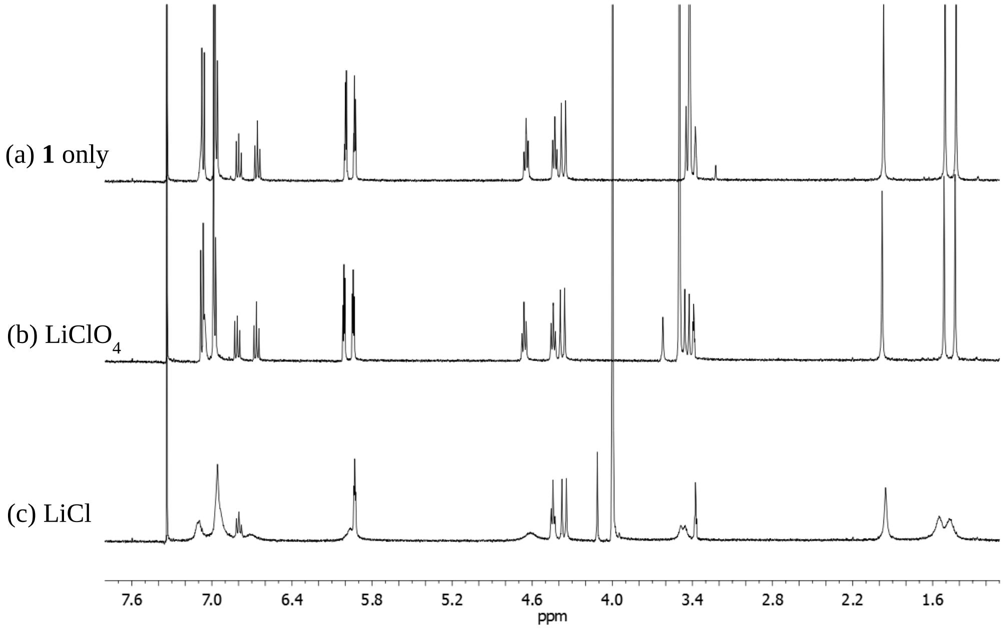

Figure S11. ^{1}H NMR spectra of (a) **1** only, (b) **1** with excess LiClO4, and (c) **1** with excess LiCl in CD_3OD-$CDCl_3$ (1:9, v/v).

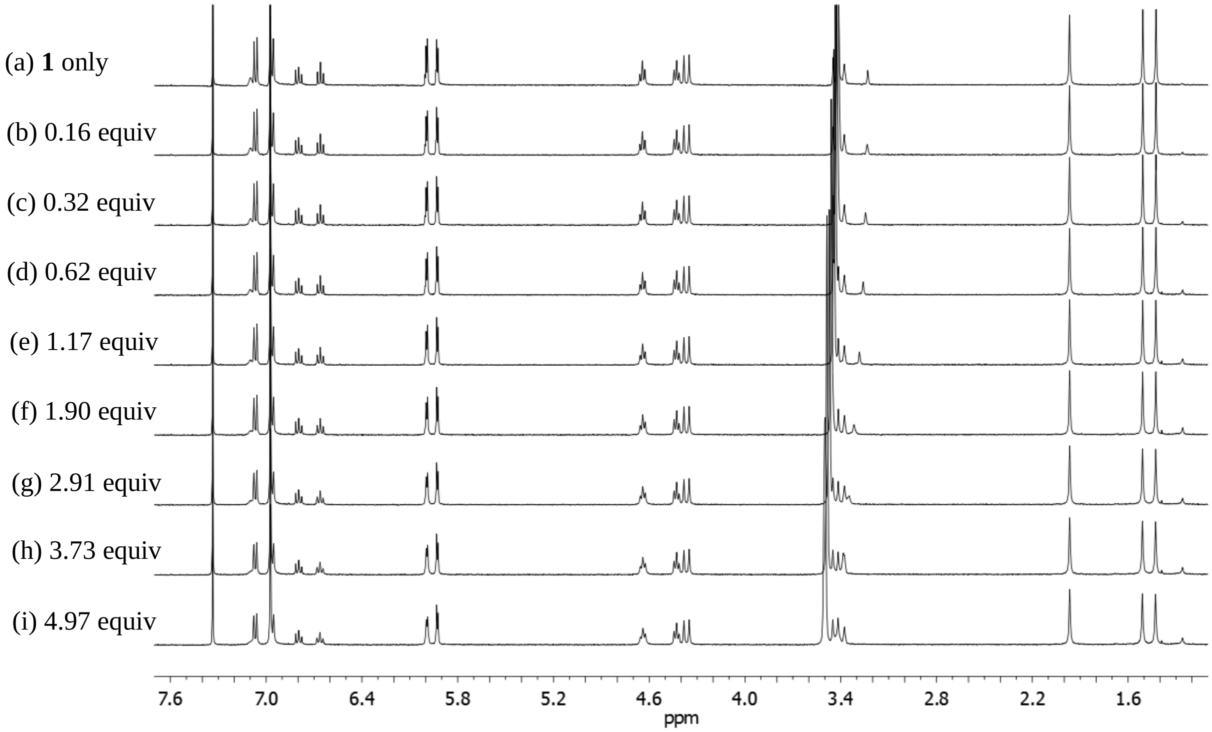

Figure S12. ^{1}H NMR spectra recorded during the titration of receptor **1** with LiCl in CD_3OD-$CDCl_3$ (1:9, v/v).

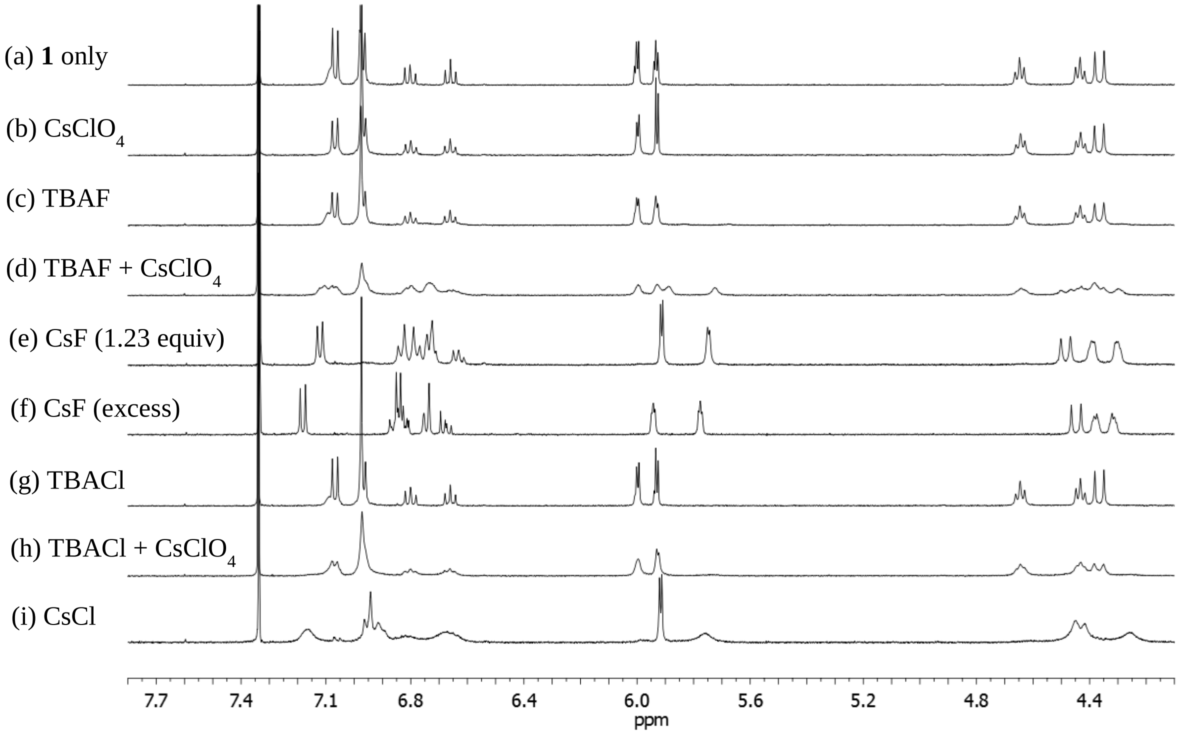

Figure S13. ^{1}H NMR spectra of (a) **1** only, (b) **1** with excess $CsClO_4$, (c) **1** with excess TBAF, (d) **1** with excess $CsClO_4$ and excess TBAF, (e) **1** with 1.23 equiv of CsF, (f) **1** with excess CsF, (g) **1** with excess TBACl, (h) **1** with excess $CsClO_4$ and excess TBACl, and (i) **1** with excess CsCl in CD_3OD-$CDCl_3$ (1:9,v/v).

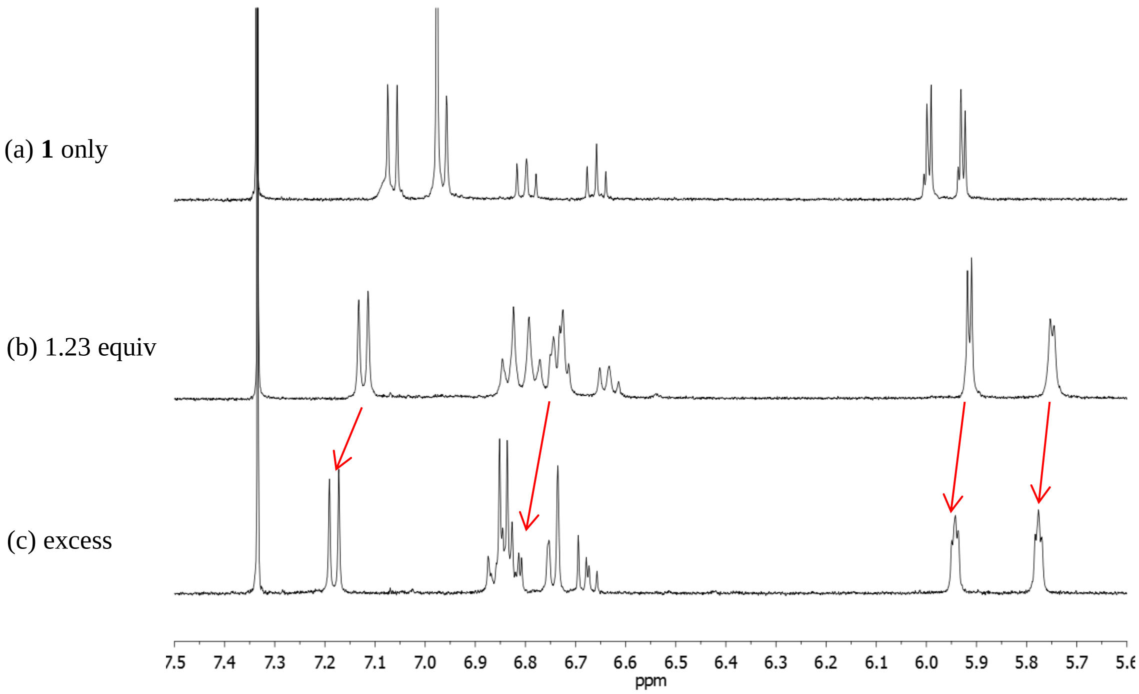

Figure S14. Partial ^{1}H NMR spectra of (a) **1** only, (b) **1** with 1.23 equiv of CsF, and (c) **1** with excess CsF in CD_3OD-$CDCl_3$ (1:9,v/v).

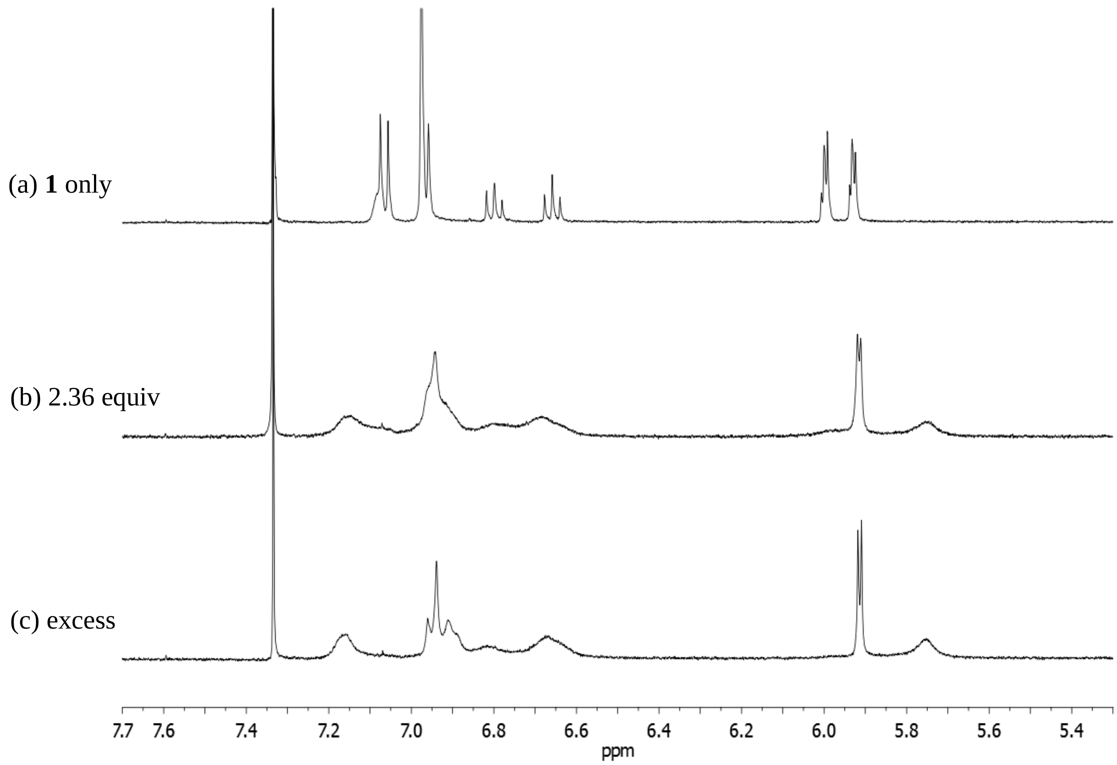

Figure S15. Partial ^{1}H NMR spectra of (a) **1** only, (b) **1** with 2.36 equiv of CsCl, and (c) **1** with excess CsCl in CD_3OD-$CDCl_3$ (1:9,v/v).

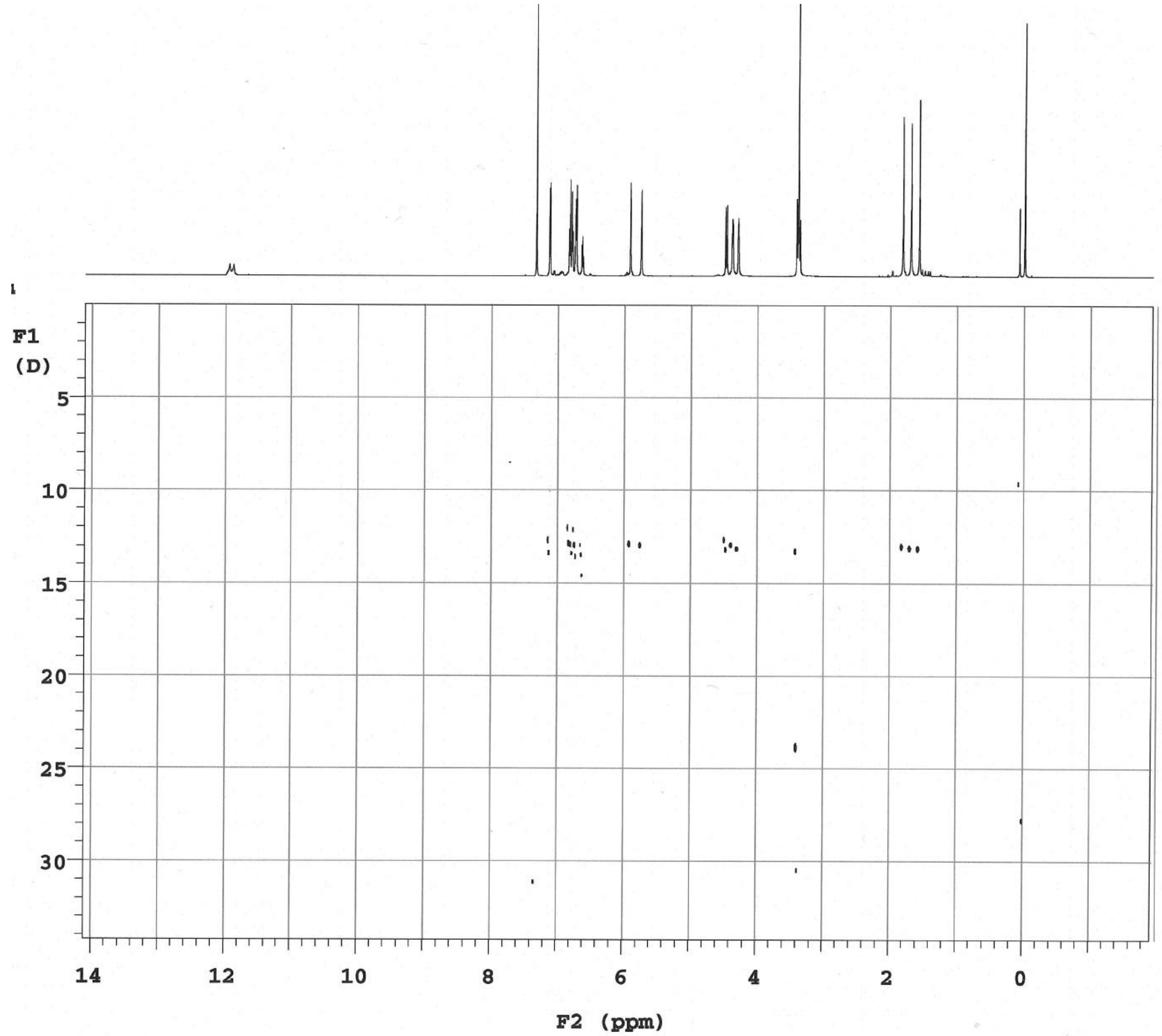

Figure S16. DOSY spectrum of compound **1** (5.07 mM) recorded in the presence of 10.1 mM of CsF in $CD_3OD/CDCl_3$ (1:9, v/v).

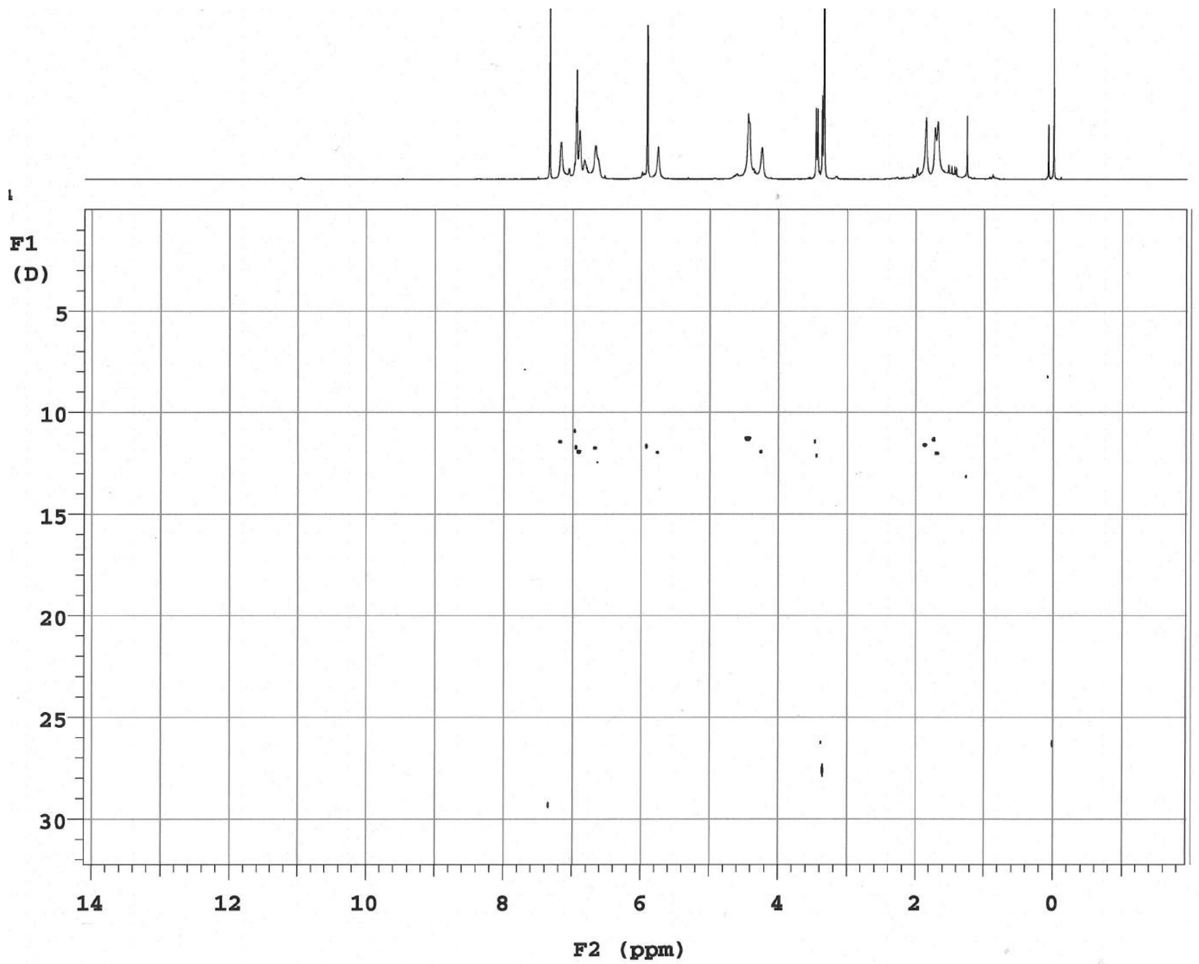

Figure S17. DOSY spectrum of compound **1** (5.07 mM) recorded in the presence of excess CsCl in $CD_3OD/CDCl_3$ (1:9, v/v).

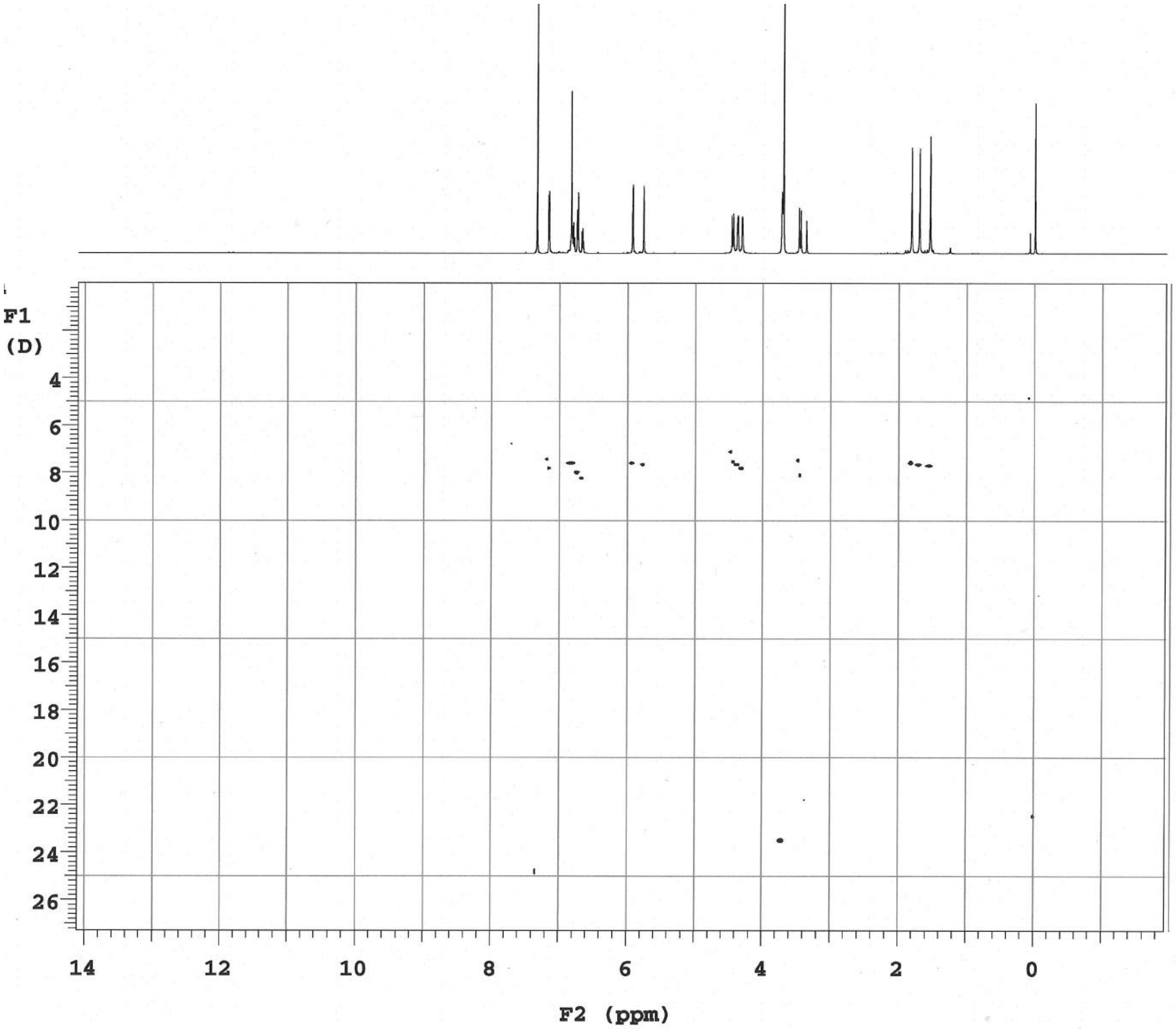

Figure S18. DOSY spectrum of compound **1** (5.07 mM) recorded in the presence of excess CsF in $CD_3OD/CDCl_3$ (1:9, v/v).

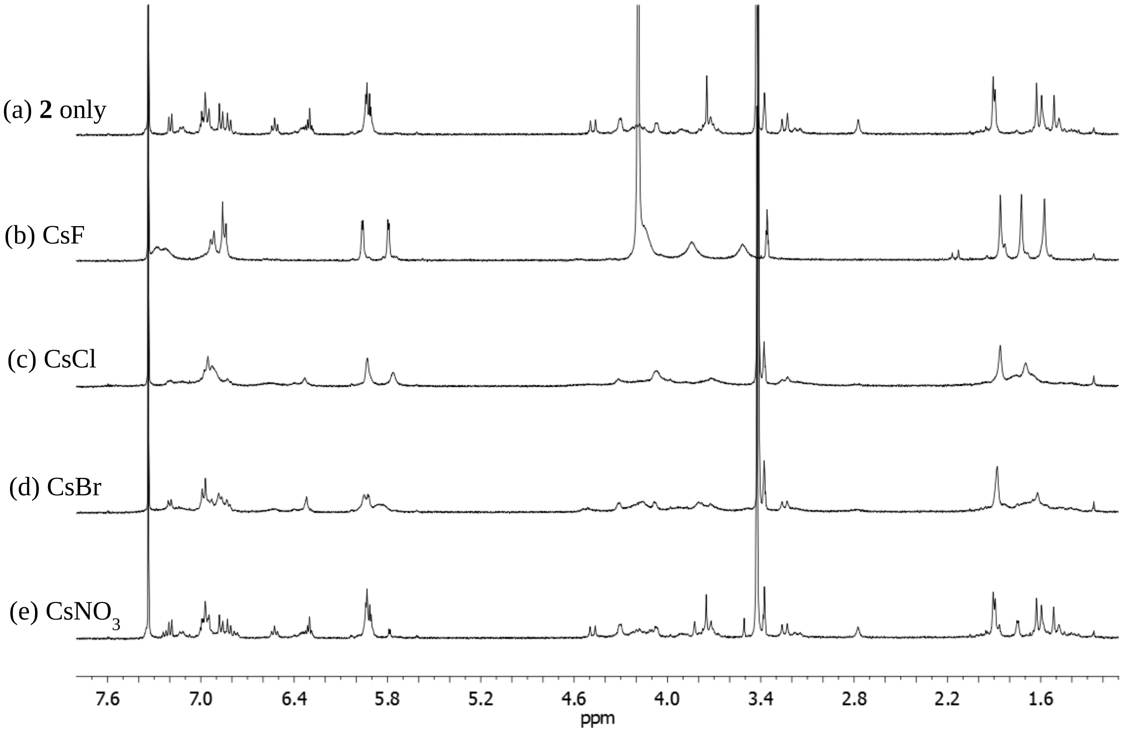

Figure S19. ^{1}H NMR spectra of (a) **2** only, (b) **2** with excess CsF, (c) **2** with excess CsCl, and (d) **2** with excess CsBr in CD_3OD-$CDCl_3$ (1:9,v/v).

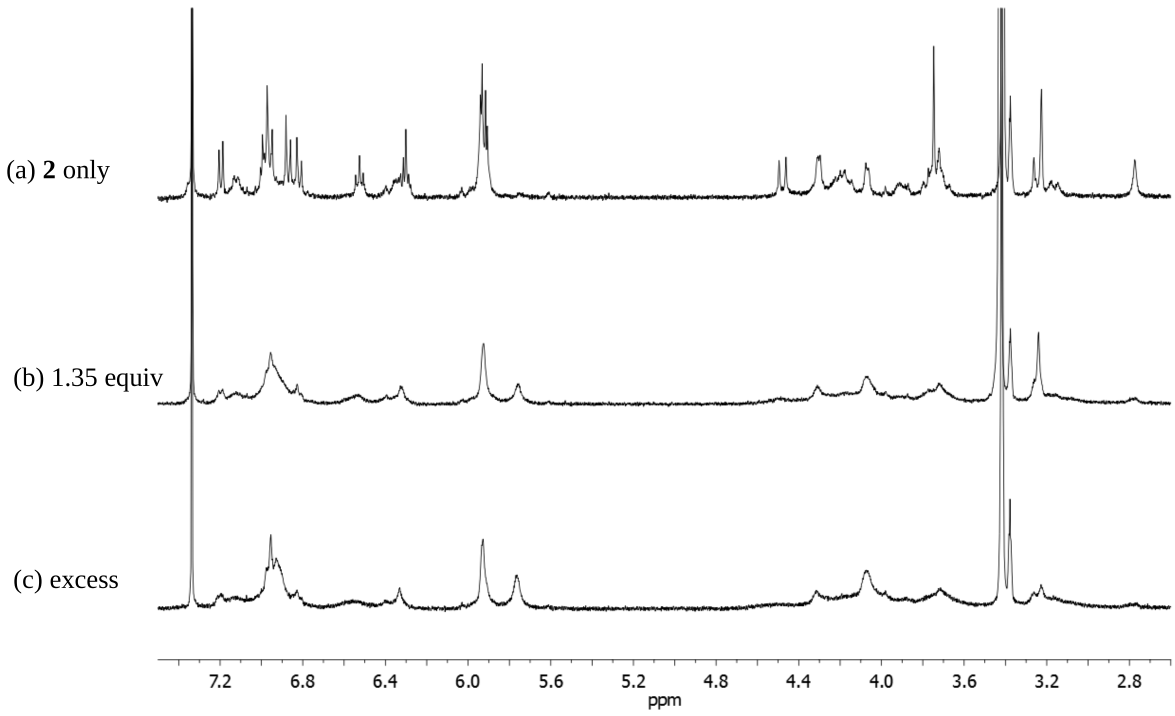

Figure S20. Partial ^{1}H NMR spectra of (a) **2** only, (b) **2** with 1.35 equiv of CsCl, and (c) **2** with excess CsCl in CD_3OD-$CDCl_3$ (1:9,v/v).

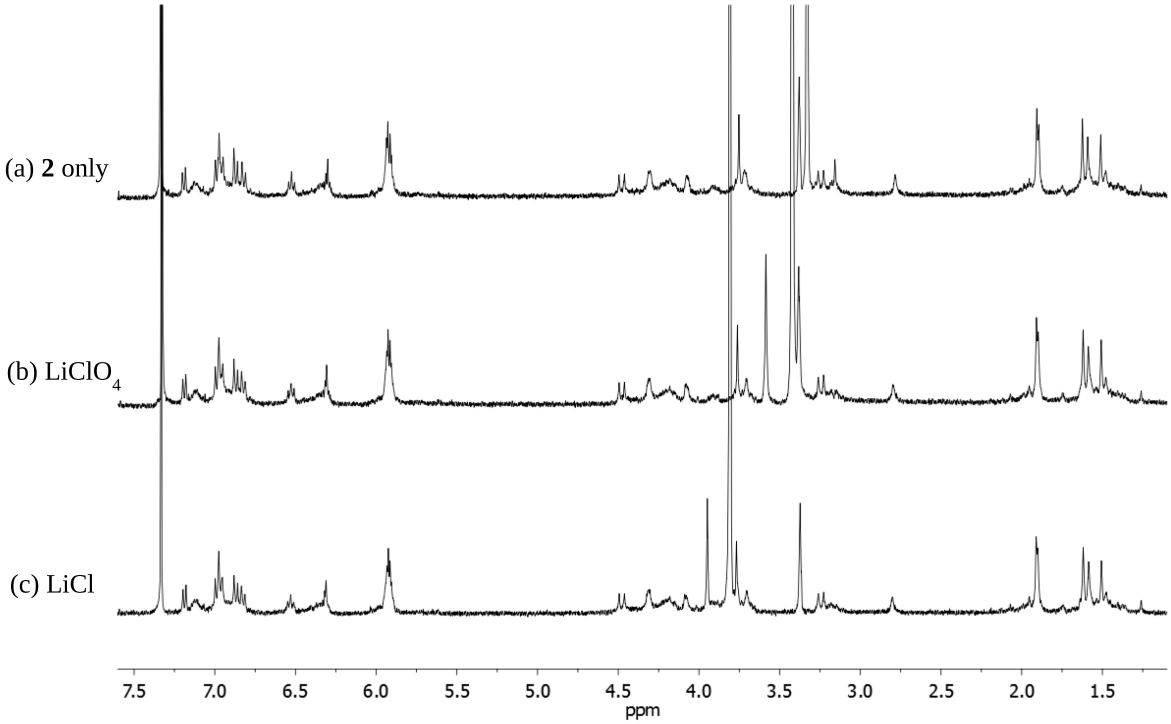

Figure S21. ^{1}H NMR spectra of (a) **2** only, (b) **2** with excess LiClO4, and (c) **2** with excess LiCl in CD_3OD-$CDCl_3$ (1:9, v/v).

X-ray Experimental for 1_2•(ethyl acetate)$_6$

Crystals grew as large, colorless prisms by slow evaporation from ethyl acetate. The data crystal was cut from a larger crystal and had approximate dimensions; 0.58 × 0.24 × 0.17 mm. The data were collected on a Rigaku SCX-Mini diffractometer with a Mercury CCD using a graphite monochromator with MoKα radiation (λ = 0.71075 Å). A total of 1440 frames of data were collected using ω-scans with a scan range of 0.5° and a counting time of 22 seconds per frame. The data were collected at 153 K using a Rigaku XStream low temperature device. Details of crystal data, data collection and structure refinement are listed in Table S1. Data reduction were performed using the Rigaku Americas Corporation's Crystal Clear version 1.40.[2] The structure was solved by direct methods using SIR97[3] and refined by full-matrix least-squares on F^2 with anisotropic displacement parameters for the non-H atoms using SHELXL-97.[4] A molecule of ethyl acetate was disordered near a crystallographic inversion center at 0, ½, 1. This molecule could not be modeled satisfactorily. The contribution to the scattering factors for this molecule was removed by the use of the utility, SQUEEZE[5] in PLATON98.[6] Structure analysis was aided by use of the programs PLATON98 and WinGX.[7] The hydrogen atoms were calculated in ideal positions with isotropic displacement parameters set to 1.2 × Ueq of the attached atom (1.5 × Ueq for methyl hydrogen atoms). The function, $\Sigma w(|F_o|^2 - |F_c|^2)^2$, was minimized, where $w = 1/[(\sigma(F_o))^2 + (0.107*P)^2 + (10.446*P)]$ and $P = (|F_o|^2 + 2|F_c|^2)/3$. $R_w(F^2)$ refined to 0.261, with R(F) equal to 0.0919 and a goodness of fit, S, = 1.18. Definitions used for calculating R(F), $R_w(F^2)$ and the goodness of fit, S, are given below.[8] The data were checked for secondary extinction effects but no correction was necessary. Neutral atom scattering factors and values used to calculate the linear absorption coefficient are from the International Tables for X-ray Crystallography (1992).[9] All figures were generated using SHELXTL/PC.[4] Tables of positional and thermal parameters, bond lengths and angles, torsion angles and figures may be found from the Cambridge Crystallographic Centre by referencing CCDC number 1025275.

Table S1. Crystal data and structure refinement for **1**•(ethyl acetate)$_3$

Empirical formula	C82 H92 N4 O12	
Formula weight	1325.60	
Temperature	153(2) K	
Wavelength	0.71075 Å	
Crystal system	Triclinic	
Space group	P-1	
Unit cell dimensions	a = 16.284(3) Å	α = 65.823(4)°.
	b = 21.681(4) Å	β = 87.933(5)°.
	c = 24.254(5) Å	γ = 77.172(5)°.
Volume	7602(3) Å^3	
Z	4	
Density (calculated)	1.158 Mg/m^3	
Absorption coefficient	0.077 mm^{-1}	
F(000)	2832	
Crystal size	0.58 × 0.24 × 0.17 mm^3	
Theta range for data collection	2.96 to 25.00°.	
Index ranges	-19<=h<=19, -23<=k<=25, 0<=l<=28	
Reflections collected	26731	
Independent reflections	26731	
Completeness to theta = 25.00°	99.7 %	
Absorption correction	Semi-empirical from equivalents	
Max. and min. transmission	1.00 and 0.726	
Refinement method	Full-matrix-block least-squares on F^2	
Data / restraints / parameters	26731 / 816 / 1968	
Goodness-of-fit on F^2	1.189	
Final R indices [I>2sigma(I)]	R1 = 0.0919, wR2 = 0.2481	
R indices (all data)	R1 = 0.1204, wR2 = 0.2613	
Largest diff. peak and hole	0.701 and -0.498 e.Å^{-3}	

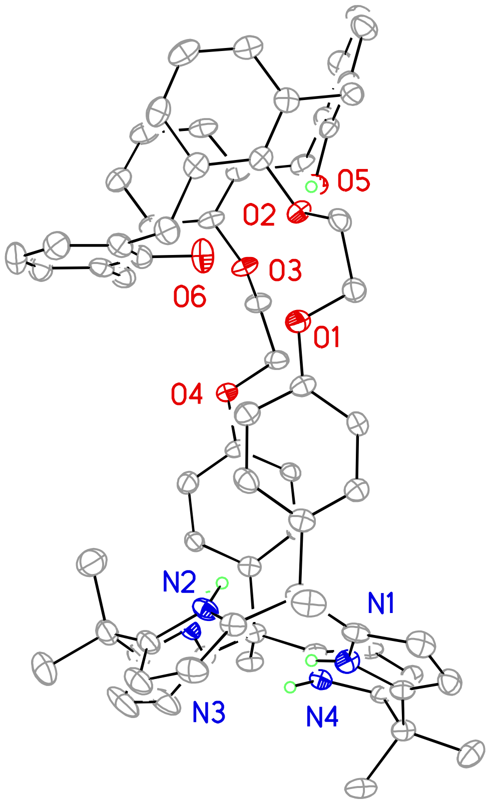

Figure S22. View of molecule 1 of **1** showing a partial atom labeling scheme. Displacement ellipsoids are scaled to the 50% probability level. Most hydrogen atoms have been omitted for clarity.

X-ray Experimental for 1•$CHCl_3$• $(EtOH)_2$

Crystals grew as large, colorless prisms by slow evaporation from a mixture of chloroform and ethanol. The data crystal was cut from a larger crystal and had approximate dimensions; 0.33 × 0.31 × 0.10 mm. The data were collected on a Rigaku SCX-Mini diffractometer with a Mercury CCD using a graphite monochromator with MoKα radiation (λ = 0.71075 Å). A total of 720 frames of data were collected using ω-scans with a scan range of 1° and a counting time of 35 seconds per frame. The data were collected at 153 K using a Rigaku XStream low temperature device. Details of crystal data, data collection and structure refinement are listed in Table S2. Data reduction were performed using the Rigaku Americas Corporation's Crystal Clear version 1.40.[2] The structure was solved by direct methods using SIR97[3] and refined by full-matrix least-squares on F^2 with anisotropic displacement parameters for the non-H atoms using SHELXL-97.[10] Structure analysis was aided by use of the programs PLATON98[7] and WinGX.[6] The hydrogen atoms on carbon were calculated in ideal positions with isotropic displacement parameters set to 1.2 × Ueq of the attached atom (1.5 × Ueq for methyl hydrogen atoms).

A molecule of chloroform was disordered near the calixpyrrole calixarene molecule. The disorder was coupled with the disorder of a molecule of ethanol. The disorder of the chloroform molecule was modeled first by assigning the variable x to the site occupancy factors for atoms Cl1a, Cl2a, Cl3a, C1a and (1-x) to the site occupancy factors for the atoms of the alternate orientation composed of atoms, Cl1b, Cl2b, Cl3b and C1aa. The geometry of the molecules was restrained while refining a common isotropic displacement factor. In this way, the site occupancy for the major component of the disorder consisting of atoms, Cl1a, Cl2a, Cl3a and C1a, was 67(2)%. Because of the proximity of the ethanol molecule to the minor component of the chloroform molecule, the site occupancy factor for this ethanol molecule could not exceed 0.67. The site occupancy for the ethanol molecule was set to 0.67. The methyl carbon on this molecule was also disordered. This disorder was modeled as above. The atoms of these disordered molecules were refined anisotropically while restraining their displacement parameters to be approximately isotropic. No hydrogen atoms attached to these atoms were included in the final refinement model.

The function, $\Sigma w(|F_o|^2 - |F_c|^2)^2$, was minimized, where $w = 1/[(\sigma(F_o))^2 + (0.1153*P)^2 + (5.9795*P)]$ and $P = (|F_o|^2 + 2|F_c|^2)/3$. $R_w(F^2)$ refined to 0.218, with R(F) equal to 0.0702 and a goodness of fit, S, = 1.02. Definitions used for calculating R(F), $R_w(F^2)$ and the goodness of fit, S, are given below.[8] The data were checked for secondary extinction effects but no correction was necessary. Neutral atom scattering factors and values used to calculate the linear absorption coefficient are from the International Tables for X-ray Crystallography (1992).[9] All figures were generated using SHELXTL/PC.[4] Tables of positional and thermal parameters, bond lengths and angles, torsion angles and figures may be found from the Cambridge Crystallographic Centre by referencing CCDC number 1025276.

Table S2. Crystal data and structure refinement for **1**•$CHCl_3$• $(EtOH)_2$

Empirical formula	C75 H71 Cl3 N4 O12	
Formula weight	1326.71	
Temperature	153(2) K	
Wavelength	0.71075 Å	
Crystal system	Monoclinic	
Space group	P21/c	
Unit cell dimensions	a = 18.865(3) Å	α = 90°.
	b = 17.828(3) Å	β = 107.064(3)°.
	c = 20.315(4) Å	γ = 90°.
Volume	6532(2) $Å^3$	
Z	4	
Density (calculated)	1.349 Mg/m^3	
Absorption coefficient	0.209 mm^{-1}	
F(000)	2784	
Crystal size	0.33 × 0.31 × 0.10 mm	
Theta range for data collection	1.55 to 27.50°.	
Index ranges	-24<=h<=24, -23<=k<=23, -26<=l<=26	
Reflections collected	84909	
Independent reflections	15005 [R(int) = 0.0448]	
Completeness to theta = 27.50°	100.0 %	
Absorption correction	Semi-empirical from equivalents	
Max. and min. transmission	1.00 and 0.701	
Refinement method	Full-matrix least-squares on F^2	
Data / restraints / parameters	15005 / 107 / 858	
Goodness-of-fit on F^2	1.004	
Final R indices [I>2sigma(I)]	R1 = 0.0702, wR2 = 0.1924	
R indices (all data)	R1 = 0.0909, wR2 = 0.2184	
Largest diff. peak and hole	0.556 and -0.934 e.$Å^{-3}$	

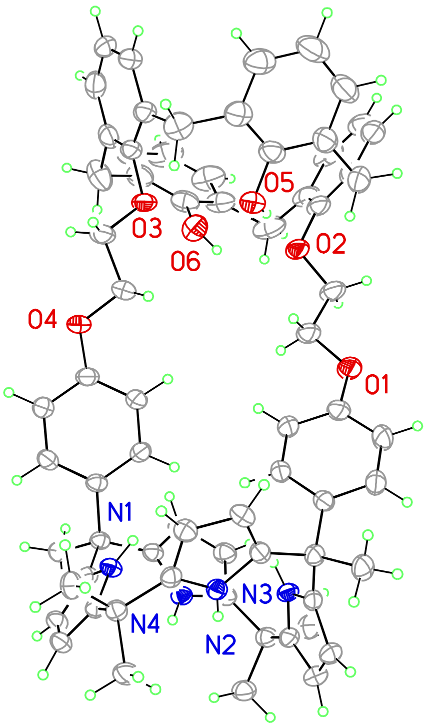

Figure S23. View of **1** showing a partial atom labeling scheme of the calix[4]arene and calix[4]pyrrole subunits. Displacement ellipsoids are scaled to the 50% probability level.

X-ray Experimental for 1•TEAF• $(CH_2Cl_2)_3$

Crystals grew as colorless prisms by slow evaporation from dichloromethane. The data crystal was cut from a larger crystal and had approximate dimensions; 0.36 × 0.34 × 0.24 mm. The data were collected on a Rigaku SCX-Mini diffractometer with a Mercury CCD using a graphite monochromator with MoKα radiation (λ = 0.71075 Å). A total of 720 frames of data were collected using ω-scans with a scan range of 1° and a counting time of 25 seconds per frame. The data were collected at 153 K using a Rigaku XStream low temperature device. Details of crystal data, data collection and structure refinement are listed in Table S3. Data reduction were performed using the Rigaku Americas Corporation's Crystal Clear version 1.40.[11] The structure was solved by direct methods using SIR97[3] and refined by full-matrix least-squares on F^2 with anisotropic displacement parameters for the non-H atoms using SHELXL-97.[4] Two large regions appeared to be occupied with dichloromethane molecules that were disordered around crystallographic inversion centers. These molecules were intricately disordered. Attempts to model the disorder were unsatisfactory. The contributions to the scattering factors due to these solvent molecules were removed by use of the utility SQUEEZE[5] in PLATON98.[7] Structure analysis was aided by use of the programs PLATON98 as incorporated into WinGX.[6] The hydrogen atoms were calculated in ideal positions with isotropic displacement parameters set to 1.2 × Ueq of the attached atom (1.5 × Ueq for methyl hydrogen atoms). The function, $\Sigma w(|F_o|^2 - |F_c|^2)^2$, was minimized, where $w = 1/[(\sigma(F_o))^2 + (0.0528*P)^2 + (2.9759*P)]$ and $P = (|F_o|^2 + 2|F_c|^2)/3$. $R_w(F^2)$ refined to 0.179, with R(F) equal to 0.0738 and a goodness of fit, S, = 1.17. Definitions used for calculating R(F), $R_w(F^2)$ and the goodness of fit, S, are given below.[8] The data were checked for secondary extinction effects but no correction was necessary. Neutral atom scattering factors and values used to calculate the linear absorption coefficient are from the International Tables for X-ray Crystallography (1992).[9] All figures were generated using SHELXTL/PC.[10] Tables of positional and thermal parameters, bond lengths and angles, torsion angles and figures may be found from the Cambridge Crystallographic Centre by referencing CCDC number 1025277.

Table S3. Crystal data and structure refinement for **1**•TEAF• $(CH_2Cl_2)_3$

Empirical formula	C81 H94 Cl6 F N5 O6	
Formula weight	1465.31	
Temperature	153(2) K	
Wavelength	0.71075 Å	
Crystal system	Monoclinic	
Space group	P21/n	
Unit cell dimensions	a = 18.1082(15) Å	α = 90°.
	b = 20.262(2) Å	β = 105.284(3)°.
	c = 21.408(3) Å	γ = 90°.
Volume	7577.0(14) $Å^3$	
Z	4	
Density (calculated)	1.285 Mg/m^3	
Absorption coefficient	0.285 mm^{-1}	
F(000)	3096	
Crystal size	0.36 × 0.34 × 0.24 mm	
Theta range for data collection	3.04 to 27.49°.	
Index ranges	-23<=h<=22, 0<=k<=26, 0<=l<=27	
Reflections collected	17344	
Independent reflections	17344 [R(int) = 0.0000]	
Completeness to theta = 27.49°	99.7 %	
Absorption correction	Semi-empirical from equivalents	
Max. and min. transmission	1.00 and 0.786	
Refinement method	Full-matrix least-squares on F^2	
Data / restraints / parameters	17344 / 59 / 897	
Goodness-of-fit on F^2	1.390	
Final R indices [I>2sigma(I)]	R1 = 0.0738, wR2 = 0.1694	
R indices (all data)	R1 = 0.1056, wR2 = 0.1789	
Largest diff. peak and hole	0.663 and -0.994 e.$Å^{-3}$	

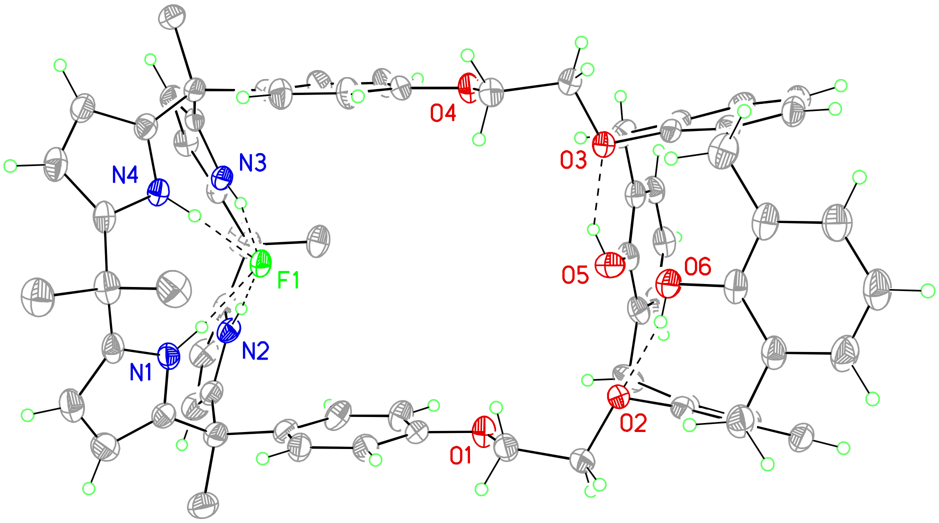

Figure S24. View of **1**•F$^-$ showing a partial atom labeling scheme. Displacement ellipsoids are scaled to the 50% probability level. The counter cation has been omitted for clarity.

X-ray Experimental for 1•CsF•$(CHCl_3)_2$•$(CH_3OH)_2$

Crystals grew as clusters of large colorless prisms by slow evaporation from chloroform and methanol. The data crystal was cut from a larger crystal and had approximate dimensions; 0.14 × 0.14 × 0.08 mm. The data were collected on a Rigaku AFC12 diffractometer with a Saturn 724+ CCD using a graphite monochromator with MoKα radiation (λ = 0.71075 Å). A total of 1608 frames of data were collected using ω-scans with a scan range of 0.5° and a counting time of 27 seconds per frame. The data were collected at 153 K using an Oxford Cryostream low temperature device. Details of crystal data, data collection and structure refinement are listed in Table S4. Data reduction were performed using the Rigaku Americas Corporation's Crystal Clear version 1.40.[11] The structure was solved by direct methods using SIR97[3] and refined by full-matrix least-squares on F^2 with anisotropic displacement parameters for the non-H atoms using SHELXL-97.[10] Structure analysis was aided by use of the programs PLATON98[7] and WinGX.[6] The hydrogen atoms were calculated in ideal positions with isotropic displacement parameters set to 1.2 × Ueq of the attached atom (1.5 × Ueq for methyl hydrogen atoms).

The cesium ion was disordered about two positions. The disorder was modeled by assigning the site occupancy factor for Cs1 to the variable x and (1-x) to the site occupancy factor for Cs1a. A common isotropic displacement parameter was refined for both atoms while refining x. In this way, the site occupancy factor for Cs1 refined to 68(2)%. The attached methanol molecule appeared to be disordered as well due to the very high and oddly shaped displacement parameters. A disorder model was applied to this solvent molecule where the site occupancy factors were assigned to those found for the cesium ions. The hydroxyl H atoms were not included in the final refinement model.

The function, $\Sigma w(|F_o|^2 - |F_c|^2)^2$, was minimized, where $w = 1/[(\sigma(F_o))^2 + (0.0366*P)^2 + (3.565*P)]$ and $P = (|F_o|^2 + 2|F_c|^2)/3$. $R^w(F^2)$ refined to 0.139, with R(F) equal to 0.0620 and a goodness of fit, S, = 1.28. Definitions used for calculating R(F), $R_w(F^2)$ and the goodness of fit, S, are given below.[8] The data were checked for secondary extinction effects but no correction was necessary. Neutral atom scattering factors and values used to calculate the linear absorption coefficient are from the International Tables for X-ray Crystallography (1992).[9] All figures were generated using SHELXTL/PC.[4] Tables of positional and thermal parameters, bond lengths and angles,

torsion angles and figures may be found from the Cambridge Crystallographic Centre by referencing CCDC number 1025274.

Table S4. Crystal data and structure refinement for **1**•CsF•$(CHCl_3)_2$•$(CH_3OH)_2$

Empirical formula	C74 H78 Cl6 Cs F N4 O8	
Formula weight	1516.01	
Temperature	100(2) K	
Wavelength	0.71073 Å	
Crystal system	Triclinic	
Space group	P-1	
Unit cell dimensions	a = 12.5824(10) Å	α = 89.080(3)°.
	b = 16.3849(12) Å	β = 89.123(3)°.
	c = 18.0859(11) Å	γ = 70.854(2)°.
Volume	3521.7(4) $Å^3$	
Z	2	
Density (calculated)	1.430 Mg/m^3	
Absorption coefficient	0.814 mm^{-1}	
F(000)	1560	
Crystal size	0.14 × 0.14 × 0.08 mm	
Theta range for data collection	3.24 to 27.48°.	
Index ranges	-16<=h<=16, -21<=k<=21, -23<=l<=23	
Reflections collected	65409	
Independent reflections	16073 [R(int) = 0.0705]	
Completeness to theta = 27.48°	99.4 %	
Absorption correction	Semi-empirical from equivalents	
Max. and min. transmission	1.00 and 0.819	
Refinement method	Full-matrix least-squares on F^2	
Data / restraints / parameters	16073 / 51 / 885	
Goodness-of-fit on F^2	1.258	
Final R indices [I>2sigma(I)]	R1 = 0.0620, wR2 = 0.1307	
R indices (all data)	R1 = 0.0849, wR2 = 0.1394	
Largest diff. peak and hole	1.019 and -0.745 e.$Å^{-3}$	

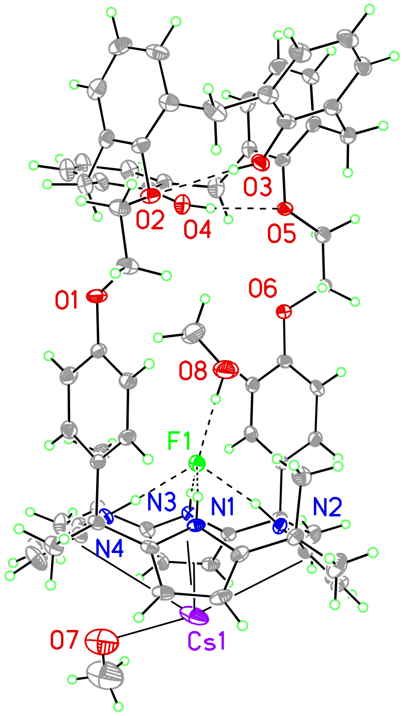

Figure S25. View of **1**•CsF showing a partial atom labeling scheme. Displacement ellipsoids are scaled to the 50% probability level. The lower occupancy atoms of the disorder are omitted.

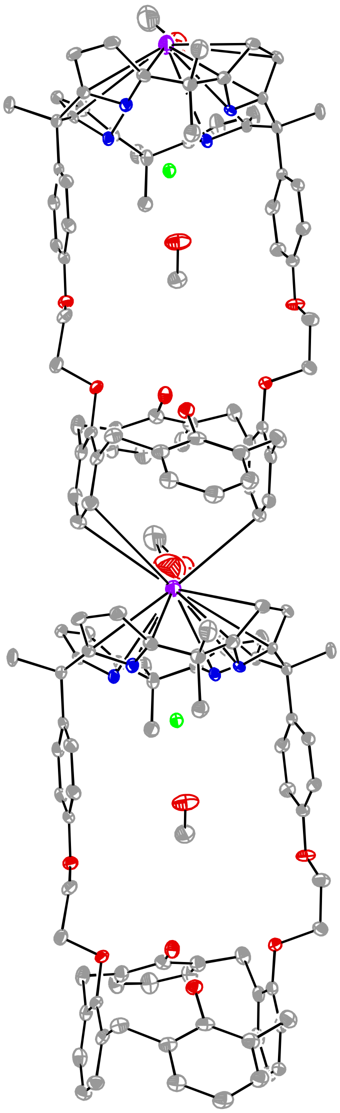

Figure S26. View of the stacking in **1**•CsF along an ab face diagonal. Displacement ellipsoids are scaled to the 50% probability level.

X-ray Experimental for 1_2•$(CsCl)_3$•$(C_2H_5OH)_2$•$CHCl_3$

Crystals grew as large, colorless prisms by slow evaporation from a mixture of chloroform and ethanol. The data crystal was cut from a larger crystal and had approximate dimensions; 0.58 × 0.24 × 0.17 mm. The data were collected on a Rigaku SCX-Mini diffractometer with a Mercury CCD using a graphite monochromator with MoKα radiation (λ = 0.71075 Å). A total of 1080 frames of data were collected using ω-scans with a scan range of 0.5° and a counting time of 22 seconds per frame. The data were collected at 223 K using a Rigaku XStream low temperature device. Details of crystal data, data collection and structure refinement are listed in Table S5. Data reduction were performed using the Rigaku Americas Corporation's Crystal Clear version 1.40.[2] The structure was solved by direct methods using SIR97[3] and refined by full-matrix least-squares on F^2 with anisotropic displacement parameters for the non-H atoms using SHELXL-97.[10] Structure analysis was aided by use of the programs PLATON98[7] and WinGX.[6] The hydrogen atoms on carbon were calculated in ideal positions with isotropic displacement parameters set to 1.2 × Ueq of the attached atom (1.5 × Ueq for methyl hydrogen atoms).

There are two coordination sites for Cs^+ in the calixarene. One Cs ion, Cs1, binds to the hydroxyl group oxygen atoms, O5 and O6, and a chloride ion, Cl1. The second Cs ion, Cs2, is pi-bound to the pyrrole groups. This latter Cs ion is disordered over two sites and lies near a crystallographic inversion center at 1, 0, 0. A second chloride ion resides on a crystallographic inversion center at 1, 1/2, 0. This ion is within H-bonding distance of an ethanol solvate molecule that is coordinated to Cs1.

A molecule of chloroform was disordered. The electron density at the Cl atom sites was not sufficiently high to account for a full occupancy chloroform molecule. The site occupancy appeared closer to 1/2. The disorder was modeled by assigning the variable x to the site occupancy factors for one component of the disordered molecule consisting of atoms Cl1b, Cl2b, Cl3b and C1b. The factor (1/2-x) was assigned to the site occupancy of the alternate atoms, Cl1c, Cl2c, Cl3c and C1c. The geometry of these molecules was restrained to be equivalent throughout the refinement process. A common site occupancy factor was refined while refining x. The site occupancy for the major component, atoms Cl1b, Cl2b, Cl3b and C1b, refined to 27(2)%. The disorder for the Cs ions, Cs2 and Cs2a, was refined in a similar fashion. The disordered atoms

were refined anisotropically while restraining their displacement parameters to be approximately isotropic.

The hydroxyl group H atoms on O5, O6 and O1a, could not be located in a difference electron density map and were not included in the final refinement model.

The function, $\Sigma w(|F_o|^2 - |F_c|^2)^2$, was minimized, where $w = 1/[(\sigma(F_o))^2 + (0.0703*P)^2 + (3.6303*P)]$ and $P = (|F_o|^2 + 2|F_c|^2)/3$. $R_w(F^2)$ refined to 0.272, with R(F) equal to 0.103 and a goodness of fit, S, = 1.54. Definitions used for calculating R(F), $R_w(F^2)$ and the goodness of fit, S, are given below.[8] The data were checked for secondary extinction effects but no correction was necessary. Neutral atom scattering factors and values used to calculate the linear absorption coefficient are from the International Tables for X-ray Crystallography (1992).[9] All figures were generated using SHELXTL/PC.[4] Tables of positional and thermal parameters, bond lengths and angles, torsion angles and figures may be found from the Cambridge Crystallographic Centre by referencing CCDC number 1025273.

Table S5. Crystal data and structure refinement for $\mathbf{1}_2$•$(CsCl)_3$•$(C_2H_5OH)_2$•$CHCl_3$

Identification code	shelxl	
Empirical formula	C145 H149 Cl6 Cs3 N8 O14	
Formula weight	2839.15	
Temperature	153(2) K	
Wavelength	0.71075 Å	
Crystal system	Triclinic	
Space group	P-1	
Unit cell dimensions	a = 11.649(3) Å	α = 60.833(5)°.
	b = 18.299(5) Å	β = 80.132(6)°.
	c = 18.362(5) Å	γ = 86.221(6)°.
Volume	3366.6(16) $Å^3$	
Z	1	
Density (calculated)	1.400 Mg/m^3	
Absorption coefficient	0.992 mm^{-1}	
F(000)	1454	
Crystal size	0.58 × 0.24 × 0.17 mm	
Theta range for data collection	3.08 to 25.00°.	
Index ranges	-13<=h<=13, -21<=k<=21, -21<=l<=21	
Reflections collected	29697	
Independent reflections	11824 [R(int) = 0.1060]	
Completeness to theta = 25.00°	99.8 %	
Absorption correction	Semi-empirical from equivalents	
Max. and min. transmission	1.00 and 0.690	
Refinement method	Full-matrix least-squares on F^2	
Data / restraints / parameters	11824 / 97 / 860	
Goodness-of-fit on F^2	1.534	
Final R indices [I>2sigma(I)]	R1 = 0.1032, wR2 = 0.2453	
R indices (all data)	R1 = 0.1782, wR2 = 0.2723	
Largest diff. peak and hole	1.495 and -1.950 e.$Å^{-3}$	

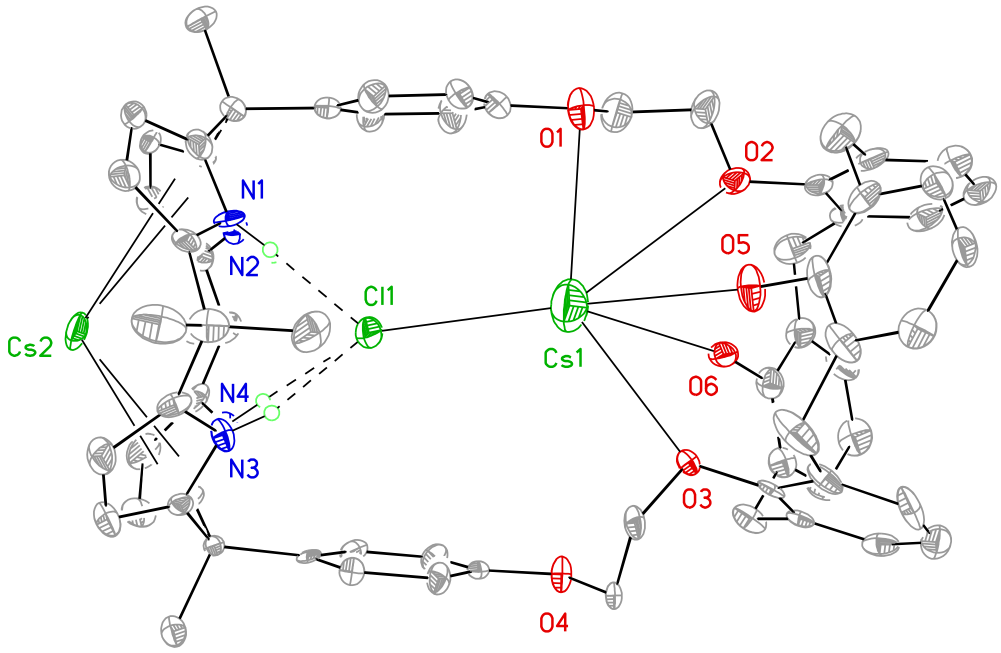

Figure S27. View of **1**•CsCl showing a partial atom labeling scheme. Displacement ellipsoids are scaled to the 50% probability level. The hydrogen atoms have been omitted for clarity.

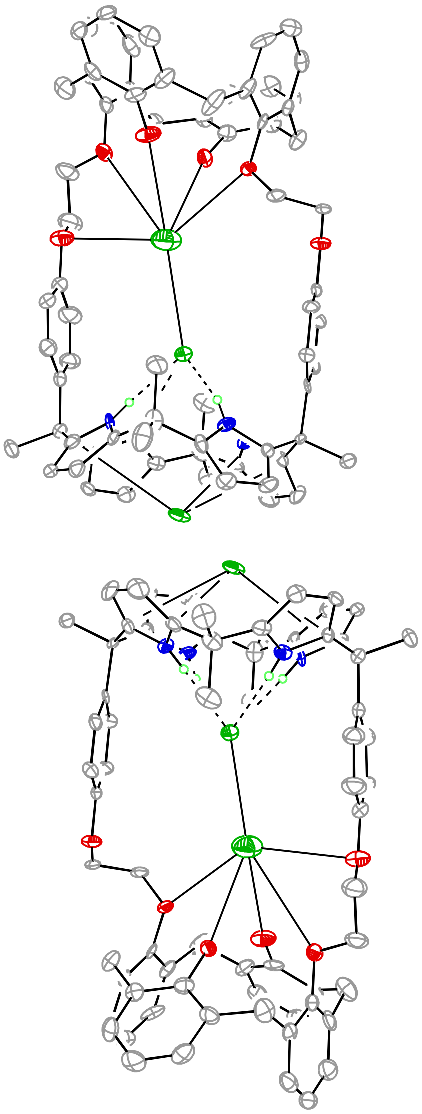

Figure S28. View of the dimer formed in **1**•CsCl. The Cs ion attached to the pyrrole groups is disordered. Displacement ellipsoids are scaled to the 50% probability level. The hydrogen atoms have been omitted for clarity.

X-ray Experimental for 2•$(CH_3CN)_3$

Crystals grew as colorless needles by slow evaporation from a solution of DMSO, DCM and acetonitrile. The data crystal had approximate dimensions; 0.22 × 0.06 × 0.06 mm. The data were collected on an Agilent Technologies SuperNova Dual Source diffractometer using a μ-focus Cu Kα radiation source (λ = 1.54184 Å) with collimating mirror monochromators. A total of 987 frames of data were collected using ω-scans with a scan range of 1° and a counting time of 15 seconds per frame for frames collected at a θ angle of +/- 40.84° and a counting time of 45 seconds for frames collected at a θ angle of 108.3°. The data were collected at 150 K using an Oxford Cryostream low temperature device. Details of crystal data, data collection and structure refinement are listed in Table S6. Data collection, unit cell refinement and data reduction were performed using Agilent Technologies CrysAlisPro V 1.171.37.31.[12] The structure was solved by direct methods using SIR97[3] and refined by full-matrix least-squares on F^2 with anisotropic displacement parameters for the non-H atoms using SHELXL-2013.[10] Structure analysis was aided by use of the programs PLATON98[7] and WinGX.[6] The hydrogen atoms on carbon were calculated in ideal positions with isotropic displacement parameters set to 1.2 × Ueq of the attached atom (1.5 × Ueq for methyl hydrogen atoms).

One of the two methoxyphenyl groups was disordered resulting in a mix of the cone conformation and the partial cone conformation. The disorder was modeled by assigning the variable x to the site occupancy factors for one component of the disorder and (1-x) to the site occupancy factors for the alternate conformer. The geometry of the two components was restrained to be equivalent throughout the refinement process. A common isotropic displacement parameter was refined for all the atoms involved while refining x. In this way, the major component of the disorder consisting of atoms, O6, C56, C57, C58, C59, C60 and C61, had a site occupancy of 83(2)%. These atoms were refined anisotropically in the final refinement model. Two of the three acetonitrile molecules were also disordered. The disorder of these solvent molecules was modeled in the same fashion as that described above.

The function, $\Sigma w(|F_o|^2 - |F_c|^2)^2$, was minimized, where $w = 1/[(\sigma(F_o))^2 + (0.1098*P)^2 + (5.0552*P)]$ and $P = (|F_o|^2 + 2|F_c|^2)/3$. $R_w(F^2)$ refined to 0.230, with R(F) equal to 0.0773 and a goodness of fit, S, = 1.05. Definitions used for calculating R(F), $R_w(F^2)$ and the goodness of fit, S,

are given below.[8] The data were checked for secondary extinction effects but no correction was necessary. Neutral atom scattering factors and values used to calculate the linear absorption coefficient are from the International Tables for X-ray Crystallography (1992).[9] All figures were generated using SHELXTL/PC.[4] Tables of positional and thermal parameters, bond lengths and angles, torsion angles and figures may be found from the Cambridge Crystallographic Centre by referencing CCDC number 1025278.

Table S6. Crystal data and structure refinement for **2**•$(CH_3CN)_3$

Empirical formula	C78 H81 N7 O6	
Formula weight	1212.49	
Temperature	150(2) K	
Wavelength	1.54184 Å	
Crystal system	monoclinic	
Space group	P 21/n	
Unit cell dimensions	a = 19.8344(3) Å	α = 90°.
	b = 17.7442(3) Å	β = 109.128(2)°.
	c = 20.5845(4) Å	γ = 90°.
Volume	6844.6(2) $Å^3$	
Z	4	
Density (calculated)	1.177 Mg/m^3	
Absorption coefficient	0.590 mm^{-1}	
F(000)	2584	
Crystal size	0.220 × 0.060 × 0.060 mm	
Theta range for data collection	3.372 to 74.295°.	
Index ranges	-24<=h<=24, -12<=k<=21, -25<=l<=25	
Reflections collected	38524	
Independent reflections	13648 [R(int) = 0.0280]	
Completeness to theta = 67.684°	100.0 %	
Absorption correction	Semi-empirical from equivalents	
Max. and min. transmission	1.00 and 0.894	
Refinement method	Full-matrix least-squares on F^2	
Data / restraints / parameters	13648 / 141 / 892	
Goodness-of-fit on F^2	1.037	
Final R indices [I>2sigma(I)]	R1 = 0.0773, wR2 = 0.2082	
R indices (all data)	R1 = 0.1020, wR2 = 0.2297	
Extinction coefficient	n/a	
Largest diff. peak and hole	0.943 and -0.337 e.$Å^{-3}$	

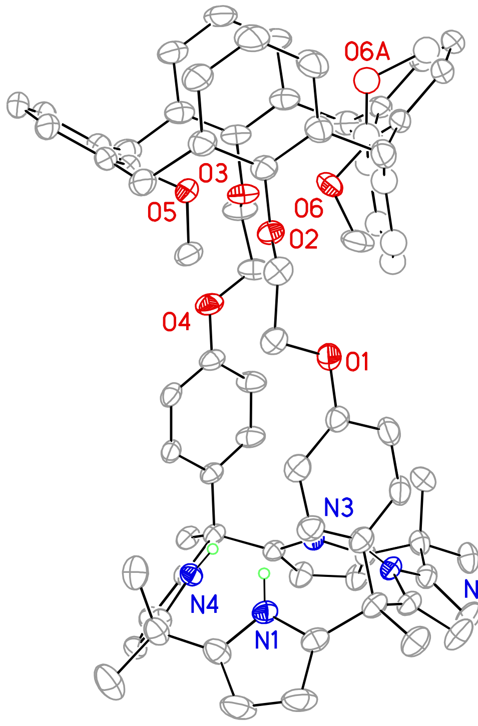

Figure S29. View of **2** showing the disordered methoxyphenyl group. Also shown is a partial atom labeling scheme. Displacement ellipsoids are scaled to the 30% probability level.

X-ray Experimental for 2_2•(TEAF)$_2$•$(CH_2Cl_2)_3$•$(H_2O)_3$

Crystals grew as clusters of colorless prisms by slow diffusion of pentane into methylene chloride. The data crystal had approximate dimensions; 0.26 × 0.21 × 0.12 mm. The data were collected on an Agilent Technologies SuperNova Dual Source diffractometer using a μ-focus Cu Kα radiation source (λ = 1.5418 Å) with collimating mirror monochromators. A total of 1950 frames of data were collected using ω-scans with a scan range of 1° and a counting time of 6 seconds per frame with a detector offset of +/- 41° and a counting time of 21 seconds per frame with a theta offset of +/- 111°. The data were collected at 153 K using an Oxford Cryostream low temperature device. Details of crystal data, data collection and structure refinement are listed in Table S7. Data collection, unit cell refinement and data reduction were performed using Agilent Technologies CrysAlisPro V 1.171.37.31.[12] The structure was solved by direct methods using SIR97[3] and refined by full-matrix least-squares on F^2 with anisotropic displacement parameters for the non-H atoms using SHELXL-2013.[10] Structure analysis was aided by use of the programs PLATON98[7] and WinGX.[6] The hydrogen atoms were calculated in ideal positions with isotropic displacement parameters set to 1.2 × Ueq of the attached atom (1.5 × Ueq for methyl hydrogen atoms).

A large region in the unit cell near an inversion center at 0,0,0 contained several molecules of disordered solvent. The solvent appeared to be composed of DCM and water molecules. Attempts to model the disorder were unsatisfactory. The contributions to the scattering factors due to these solvent molecules were removed by use of the utility SQUEEZE[5] in PLATON98. PLATON98 was used as incorporated in WinGX.

Three of the four methoxyphenyl groups were disordered. The disorder meant that there was a mixture of calix[4]arene molecules with the cone and partial cone conformations in the crystal. The disorder for these groups and for a disordered DCM molecule was modeled in essentially the same fashion. The variable x was assigned to the site occupancy factors for the atoms of one conformer, while (1-x) was assigned to the site occupancy factors of the atoms of the alternate conformer. The geometry of the two conformers was restrained to be equivalent throughout the refinement process. A common isotropic displacement parameter was refined while refining the variable x. H atoms were added in idealized positions for the methyl and aromatic C atoms. No

hydrogen atoms were calculated for the methylene carbon atoms that were attached to these disordered groups.

The non-H atoms were refined with anisotropic displacement parameters that were restrained to be approximately isotropic in the final refinement model.

The function, $\Sigma w(|F_o|^2 - |F_c|^2)^2$, was minimized, where $w = 1/[(\sigma(Fo))2 + (0.1884*P)^2 + (4.1202*P)]$ and $P = (|F_o|^2 + 2|F_c|^2)/3$. $R_w(F^2)$ refined to 0.320, with R(F) equal to 0.0914 and a goodness of fit, S, = 1.08. Definitions used for calculating R(F), $R_w(F^2)$ and the goodness of fit, S, are given below.[8] The data were checked for secondary extinction effects but no correction was necessary. Neutral atom scattering factors and values used to calculate the linear absorption coefficient are from the International Tables for X-ray Crystallography (1992).[9] All figures were generated using SHELXTL/PC.[4] Tables of positional and thermal parameters, bond lengths and angles, torsion angles and figures may be found from the Cambridge Crystallographic Centre by referencing CCDC number 1025281.

Table S7. Crystal data and structure refinement for $\mathbf{2}_2$•$(TEAF)_2$•$(CH_2Cl_2)_3$•$(H_2O)_3$

Empirical formula	C163 H193 Cl6 F2 N10 O15	
Formula weight	2782.96	
Temperature	150(2) K	
Wavelength	1.54184 Å	
Crystal system	triclinic	
Space group	P -1	
Unit cell dimensions	a = 17.7069(4) Å	α = 71.903(2)°.
	b = 19.2391(3) Å	β = 75.381(2)°.
	c = 27.8205(6) Å	γ = 69.249(2)°.
Volume	8315.5(3) $Å^3$	
Z	2	
Density (calculated)	1.111 Mg/m^3	
Absorption coefficient	1.431 mm^{-1}	
F(000)	2962	
Crystal size	0.260 × 0.210 × 0.120 mm	
Theta range for data collection	3.374 to 75.612°.	
Index ranges	-22<=h<=22, -24<=k<=23, -34<=l<=34	
Reflections collected	94769	
Independent reflections	33797 [R(int) = 0.0354]	
Completeness to theta = 67.684°	100.0 %	
Absorption correction	Semi-empirical from equivalents	
Max. and min. transmission	1.00 and 0.879	
Refinement method	Full-matrix-block least-squares on F^2	
Data / restraints / parameters	33797 / 1587 / 1964	
Goodness-of-fit on F^2	1.017	
Final R indices [I>2sigma(I)]	R1 = 0.0914, wR2 = 0.2854	
R indices (all data)	R1 = 0.1159, wR2 = 0.3199	
Extinction coefficient	n/a	
Largest diff. peak and hole	1.492 and -0.803 e.$Å^{-3}$	

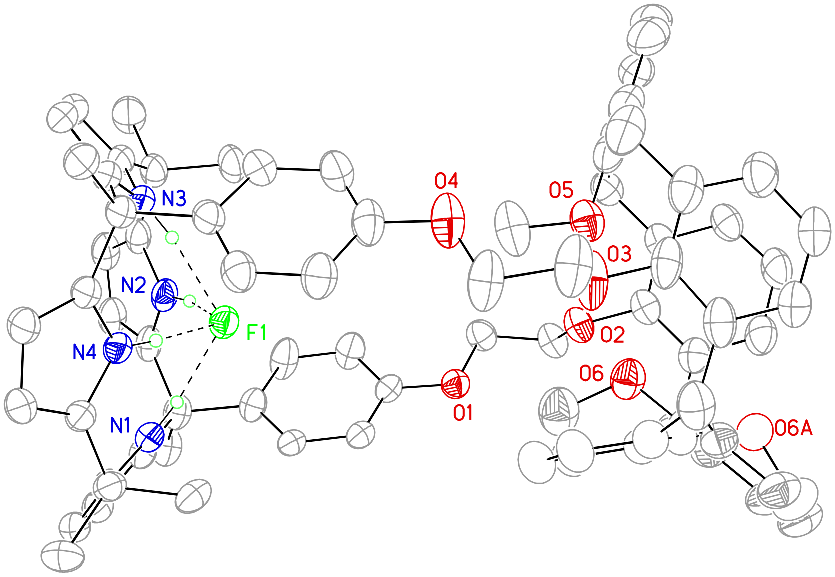

Figure S30. View of complex 1 of **2**•F⁻ showing a partial atom labeling scheme. Displacement ellipsoids are scaled to the 30% probability level. The lower occupancy atoms of the disordered methoxyphenyl group are shown as open ellipsoids.

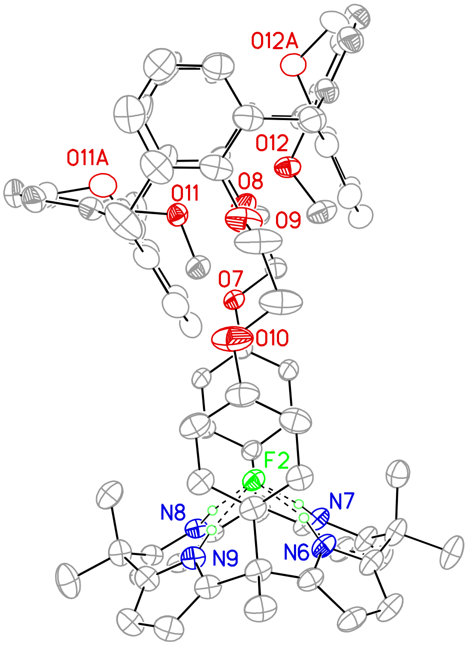

Figure S31. View of complex 2 of **2**•F$^-$ showing a partial atom labeling scheme. Displacement ellipsoids are scaled to the 30% probability level. The lower occupancy atoms of the disordered methoxyphenyl group are shown as open ellipsoids.

X-ray Experimental for 2•CsF•CH_3OH•$CHCl_3$

Crystals grew as small, colorless prisms by slow evaporation from chloroform and methanol. The data crystal had approximate dimensions; 0.18 × 0.16 × 0.10 mm. The data were collected on a Rigaku ACF-12 with a Saturn 724+ CCD using a graphite monochromator with MoKα radiation (λ = 0.71075 Å). A total of 1104 frames of data were collected using ω-scans with a scan range of 0.5° and a counting time of 45 seconds per frame. The data were collected at 100 K using a Rigaku XStream low temperature device. Details of crystal data, data collection and structure refinement are listed in Table S8. Data reduction were performed using the Rigaku Americas Corporation's Crystal Clear version 1.40.[11] The structure was solved by direct methods using SIR97[3] and refined by full-matrix least-squares on F^2 with anisotropic displacement parameters for the non-H atoms using SHELXL-2013.[10] The hydrogen atoms on carbon were calculated in ideal positions with isotropic displacement parameters set to 1.2 × Ueq of the attached atom (1.5 × Ueq for methyl hydrogen atoms).

A molecule of chloroform was disordered. Attempts to model the disorder were unsatisfactory. The contributions to the scattering factors due to these solvent molecules were removed by use of the utility SQUEEZE[5] in PLATON98.[6] Structure analysis was aided by use of the programs PLATON98 as incorporated into WinGX.[7]

The fluoride ion was disordered over two sites. At one site, the fluoride ion, F1, was H-bound to two pyrrole groups and a methanol molecule. At the alternate site, the fluoride ion, F1a, was H-bound to all four pyrrole groups. This alternate fluoride ion was H-bound to another methanol molecule. The disorder was modeled by assigning the variable x to the site occupancy factor for F1, while (1-x) was assigned to the site occupancy factor for F1a. A common isotropic displacement parameter was refined for F1 and F1a while refining x. In this way, the site occupancy for F1 refined to 78(2)%. The site occupancy for the methanol molecule H-bound to F1 was set to 0.78 on the basis of the refinement of the fluoride ions. The occupancy factors for the methanol molecule H-bound to F1a was set to 0.22. F1a, O1b and C2b were refined isotropically in the final refinement model. No H atoms were calculated in the final model for O1b and C2b.

The function, $\Sigma w(|F_o|^2 - |F_c|^2)^2$, was minimized, where $w = 1/[(\sigma(F_o))^2 + (0.1069*P)^2 + (16.7082*P)]$ and $P = (|F_o|^2 + 2|F_c|^2)/3$. $R_w(F^2)$ refined to 0.205, with R(F) equal to 0.0740 and a goodness of fit, S, = 1.04. Definitions used for calculating R(F), $R_w(F^2)$ and the goodness of fit, S, are given below.[8] The data were checked for secondary extinction effects but no correction was necessary. Neutral atom scattering factors and values used to calculate the linear absorption coefficient are from the International Tables for X-ray Crystallography (1992).[9] All figures were generated using SHELXTL/PC.[4] Tables of positional and thermal parameters, bond lengths and angles, torsion angles and figures may be found from the Cambridge Crystallographic Centre by referencing CCDC number 1025280.

Table S8. Crystal data and structure refinement for **2**•CsF•CH_3OH•$CHCl_3$

Empirical formula	C74 H77 Cl3 Cs F N4 O7	
Formula weight	1392.65	
Temperature	100(2) K	
Wavelength	0.71073 Å	
Crystal system	monoclinic	
Space group	P 21/n	
Unit cell dimensions	a = 17.1643(12) Å	α = 90°.
	b = 20.2720(15) Å	β = 99.129(2)°.
	c = 20.1109(15) Å	γ = 90°.
Volume	6909.0(9) $Å^3$	
Z	4	
Density (calculated)	1.339 Mg/m^3	
Absorption coefficient	0.710 mm^{-1}	
F(000)	2880	
Crystal size	0.180 × 0.160 × 0.100 mm	
Theta range for data collection	3.071 to 24.999°.	
Index ranges	-19<=h<=20, -23<=k<=24, -23<=l<=23	
Reflections collected	70835	
Independent reflections	12132 [R(int) = 0.0941]	
Completeness to theta = 25.242°	97.1 %	
Absorption correction	Semi-empirical from equivalents	
Max. and min. transmission	1.00 and 0.498	
Refinement method	Full-matrix least-squares on F^2	
Data / restraints / parameters	12132 / 0 / 801	
Goodness-of-fit on F^2	1.038	
Final R indices [I>2sigma(I)]	R1 = 0.0740, wR2 = 0.1865	
R indices (all data)	R1 = 0.1028, wR2 = 0.2053	
Extinction coefficient	n/a	
Largest diff. peak and hole	1.676 and -1.723 e.$Å^{-3}$	

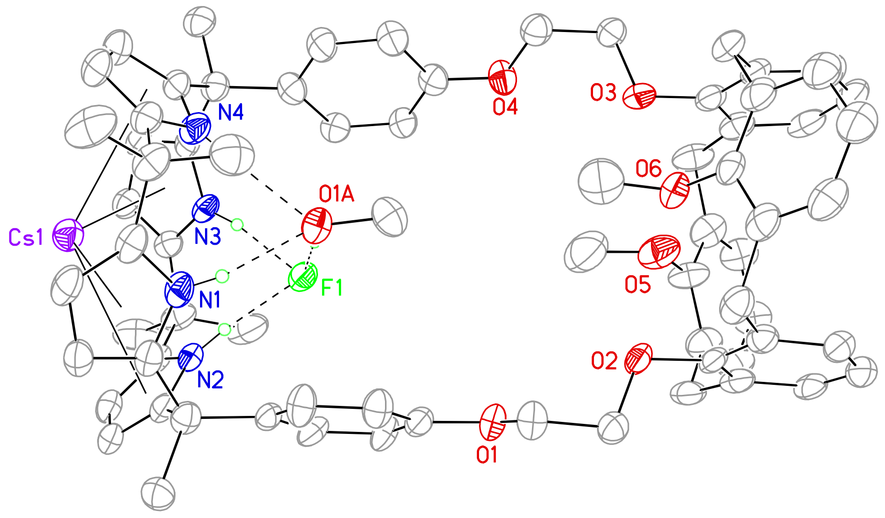

Figure S32. View of the CsF complex in **2** showing the heteroatom labeling scheme. Displacement ellipsoids are scaled to the 50% probability level. The lower occupancy fluoride ion and methanol molecule were omitted for clarity. Most H atoms have also been omitted.

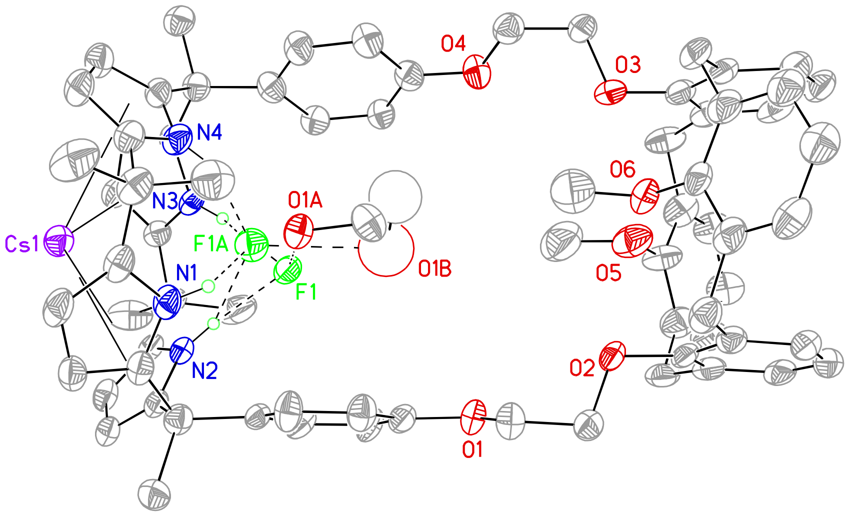

Figure S33. View of the CsF complex in **2** showing the heteroatom labeling scheme. Displacement ellipsoids are scaled to the 50% probability level. The lower occupancy fluoride ion and methanol molecule are shown as open circles. Most H atoms have also been omitted.

X-ray Experimental for 2•CsCl•CH_3OH•$CHCl_3$

Crystals grew as colorless plates by slow evaporation from chloroform and methanol. The data crystal was cut from a larger crystal and had approximate dimensions; 0.22 × 0.16 × 0.07 mm. The data were collected at -120 °C on a Nonius Kappa CCD diffractometer using a Bruker AXS Apex II detector and a graphite monochromator with MoKα radiation (λ = 0.71075 Å). Reduced temperatures were maintained by use of an Oxford Cryosystems 600 low-temperature device. A total of 535 frames of data were collected using ω-scans with a scan range of 1.0° and a counting time of 126 seconds per frame. Details of crystal data, data collection and structure refinement are listed in Table S9. Data reduction were performed using SAINT V8.27B.[13] The structure was solved by direct methods using SIR97[3] and refined by full-matrix least-squares on F^2 with anisotropic displacement parameters for the non-H atoms using SHELXL-97.[10] Structure analysis was aided by use of the programs PLATON98[7] and WinGX.[6]

A larger region of the unit cell contained some highly disordered solvent molecules. The solvent appeared to be composed of chloroform and methanol. Attempts to model the disorder were unsatisfactory. The contributions to the scattering factors due to these solvent molecules were removed by use of the utility SQUEEZE[5] in PLATON98.

The function, $\Sigma w(|F_o|^2 - |F_c|^2)^2$, was minimized, where $w = 1/[(\sigma(F_o))^2 + (0.1*P)^2]$ and $P = (|F_o|^2 + 2|F_c|^2)/3$. $R_w(F^2)$ refined to 0.445, with R(F) equal to 0.179 and a goodness of fit, S, = 1.83. Definitions used for calculating R(F),$R_w(F^2)$ and the goodness of fit, S, are given below.[8] The data were checked for secondary extinction but no correction was necessary. Neutral atom scattering factors and values used to calculate the linear absorption coefficient are from the International Tables for X-ray Crystallography (1992).[9] All figures were generated using SHELXTL/PC.[4] Tables of positional and thermal parameters, bond lengths and angles, torsion angles and figures may be found from the Cambridge Crystallographic Centre by referencing CCDC number 1025279.

Table S9. Crystal data and structure refinement for **2**•CsCl•CH_3OH•$CHCl_3$

Empirical formula	C74 H77 Cl4 Cs N4 O7	
Formula weight	1409.10	
Temperature	153(2) K	
Wavelength	0.71073 Å	
Crystal system	monoclinic	
Space group	P 2/n	
Unit cell dimensions	a = 28.4416(13) Å	α = 90°.
	b = 17.3237(8) Å	β = 90.135(3)°.
	c = 28.5922(14) Å	γ = 90°.
Volume	14087.7(11) $Å^3$	
Z	8	
Density (calculated)	1.329 Mg/m^3	
Absorption coefficient	0.732 mm^{-1}	
F(000)	5824	
Crystal size	0.22 × 0.16 × 0.07 mm	
Theta range for data collection	1.009 to 25.000°.	
Index ranges	-30<=h<=33, -20<=k<=19, -33<=l<=33	
Reflections collected	90097	
Independent reflections	24546 [R(int) = 0.2210]	
Completeness to theta = 25.242°	96.3 %	
Absorption correction	Semi-empirical from equivalents	
Max. and min. transmission	1.00 and 0.792	
Refinement method	Full-matrix least-squares on F^2	
Data / restraints / parameters	24546 / 1026 / 1557	
Goodness-of-fit on F^2	1.839	
Final R indices [I>2sigma(I)]	R1 = 0.1786, wR2 = 0.3976	
R indices (all data)	R1 = 0.3235, wR2 = 0.4448	
Extinction coefficient	n/a	
Largest diff. peak and hole	2.636 and -1.330 e.$Å^{-3}$	

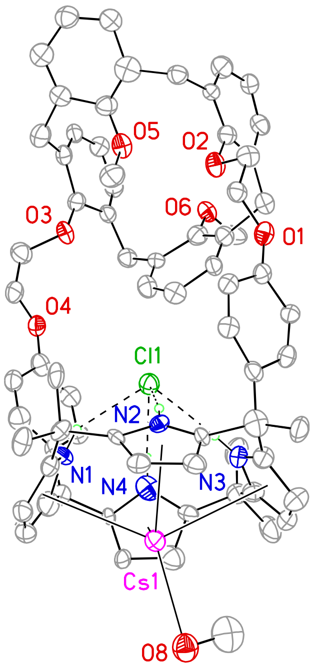

Figure S34. View of CsCl complex 1 of **2** showing the heteroatom labeling scheme. Displacement ellipsoids are scaled to the 30% probability level.

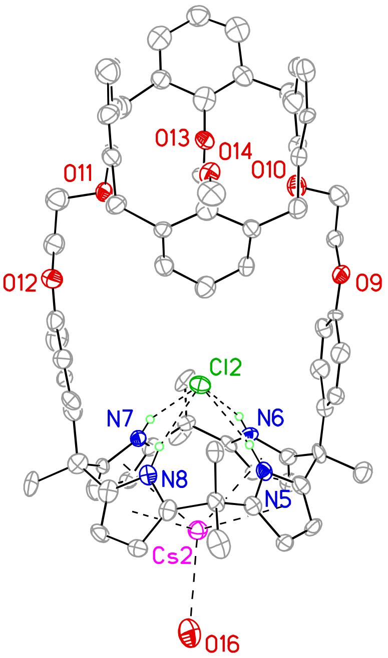

Figure S35. View of CsCl complex 2 of **2** showing the heteroatom labeling scheme. Displacement ellipsoids are scaled to the 30% probability level.

Calculation data for modeling

RI-MP2/aug-cc-pVDZ calculations were performed using Spartan ’10 available from Wavefunction, Inc., 18401 Von Karman Ave., Irvine, CA 92612.

Optimized geometries and absolute energies from the RI-MP2 calculations:

```
chloride anion,  E= -459.7227530 hartree



22
1,3-methyl-2-methoxybenzene,  E= -424.1619636 hartree

C       -0.334351     -0.208377      0.000000
C        2.418690      0.267240     -0.000000
C        0.337323     -0.101855      1.237847
C        0.337323     -0.101855     -1.237847
C        1.726265      0.139119     -1.217494
C        1.726265      0.139119      1.217494
H        2.268396      0.217227     -2.165990
H        2.268396      0.217227      2.165990
H        3.497358      0.450204     -0.000000
C       -0.419670     -0.276810      2.535033
H       -1.065630     -1.166825      2.490115
H       -1.067040      0.590143      2.747700
H        0.281957     -0.386427      3.375555
C       -0.419670     -0.276810     -2.535033
H       -1.065630     -1.166825     -2.490115
H        0.281957     -0.386427     -3.375555
H       -1.067040      0.590143     -2.747700
O       -1.701469     -0.483982      0.000000
C       -2.480558      0.726129      0.000000
H       -2.268107      1.330252      0.897884
H       -3.534470      0.417511      0.000000
H       -2.268107      1.330252     -0.897884

23
1-3-methyl-2-methoxybenzene chloride complex, E= -883.8979570 hartree

C        1.469600     -0.001209     -0.218411
C        0.786737      1.232994     -0.190743
C       -0.622365      1.205608     -0.116047
C       -1.336282     -0.006584     -0.069518
C       -0.617882     -1.216150     -0.117481
C        0.791228     -1.237918     -0.192706
H       -1.168673     -2.163518     -0.103130
H       -2.437173     -0.007506     -0.008898
H       -1.177209      2.150587     -0.100782
Cl      -4.818750      0.011700      0.160249
C        1.563416     -2.537070     -0.275557
H        2.292143     -2.510154     -1.101537
H        0.870769     -3.376983     -0.439486
H        2.126367     -2.742321      0.651615
C        1.553190      2.535787     -0.269827
H        2.302570      2.503461     -1.076650
H        2.090498      2.755794      0.669074
H        0.860080      3.369477     -0.460847
O        2.872168      0.000249     -0.313615
C        3.467365      0.007731      0.990563
```

```
H        3.171156      0.908505      1.555474
H        4.557335      0.005195      0.842289
H        3.168267     -0.884909      1.566959
```

MMFF94 calculations were performed using PCModel, version 9.3, from Serena Software, Bloomington, IN 47402.

Parameters added to treat the cesium cation and torsion angles in the calixpyrrole.

```
vdwmmff      236   2.2000   6.0000    4.0000    1.3000
torsion       63    1   63   39      0.0000 +1   -1.5000 -2   0.0000  +3
torsion       63    1   63   64      0.0000 +1   -1.5000 -2   0.0000  +3
torsion        1    1   63   39      0.0000 +1    0.0000 -2   0.3000  +3
torsion        1    1   63   64      0.0000 +1    0.0000 -2   0.3000  +3
```

```
156
CsF complex of 2 in the x-ray conformation from CsCl
F       -0.273800     -3.987300     -0.346900
Cs      -0.103500     -7.832400     -0.709700
O        0.612500      0.653400     -4.263800
O       -0.079700      3.105700     -2.630300
O       -1.266800      2.505100      3.178000
O       -1.165600     -0.246200      4.435500
O       -1.676700      4.006300      0.192900
O        2.998500      3.803700      1.127500
N        1.797400     -5.144500     -1.819400
N       -1.524800     -5.219200     -2.504000
N       -2.213900     -5.567800      0.867100
N        1.107500     -5.493500      1.555800
C        2.839600     -5.794100     -1.182300
C        3.292300     -6.761400     -2.062900
C        2.528200     -6.672800     -3.249600
C        1.604800     -5.653300     -3.093500
C        0.521300     -5.092900     -4.032600
C       -0.826000     -5.700000     -3.599400
C       -1.572900     -6.749400     -4.106900
C       -2.738600     -6.884300     -3.317800
C       -2.708700     -5.914500     -2.330600
C       -3.691700     -5.575300     -1.209700
C       -3.208500     -6.167400      0.114500
C       -3.580400     -7.310200      0.801100
C       -2.819200     -7.379900      1.990800
C       -1.976600     -6.282100      2.030200
C       -0.928900     -5.826600      3.062800
C        0.455800     -6.234600      2.528100
C        1.286200     -7.301200      2.826900
C        2.454200     -7.185400      2.037900
C        2.340600     -6.046600      1.259400
C        3.283200     -5.419300      0.232000
C        0.832800     -5.525900     -5.494500
C       -5.092900     -6.148500     -1.555400
C       -3.901600     -4.045100     -1.094400
C       -1.192600     -6.550600      4.414900
C        4.730600     -5.925300      0.475500
C        3.359900     -3.881900      0.406700
C        0.512100     -3.537900     -4.101100
C        1.700400     -2.796000     -3.958700
C        1.695300     -1.400500     -4.028500
C        0.509300     -0.709500     -4.258700
```

C	-0.668300	-1.420300	-4.460800
C	-0.663400	-2.819200	-4.394200
C	-0.554400	1.478100	-4.340700
C	-0.156500	2.922400	-4.052000
C	0.465200	4.298900	-2.223600
C	1.813700	4.321100	-1.828100
C	2.396500	5.557400	-1.510800
C	1.643100	6.729600	-1.541500
C	0.284000	6.676400	-1.844800
C	-0.333000	5.454900	-2.159600
C	-1.841300	5.403900	-2.318400
C	-2.619100	5.804500	-1.074200
C	-2.494600	5.102200	0.139100
C	-3.108600	5.556800	1.321000
C	-3.947600	6.678300	1.246600
C	-4.133500	7.347700	0.036300
C	-3.465400	6.922200	-1.112700
C	-2.859800	4.888600	2.664600
C	-1.411600	4.909200	3.117400
C	-0.655200	3.732100	3.257900
C	0.739000	3.777400	3.430000
C	1.349200	5.027500	3.613600
C	0.596500	6.200100	3.578900
C	-0.770400	6.143000	3.313700
C	1.600200	2.530600	3.330000
C	1.695800	1.954200	1.928300
C	2.303800	2.647900	0.866000
C	2.184500	2.201400	-0.462800
C	1.577100	0.957600	-0.693200
C	1.059500	0.206200	0.359200
C	1.097000	0.714800	1.655400
C	2.617800	3.045400	-1.648600
C	-2.276800	2.742100	-0.060500
C	4.386000	3.623100	1.392500
C	-1.882400	2.049200	4.392000
C	-2.302800	0.593600	4.213600
C	-1.203900	-1.576200	4.123100
C	0.005100	-2.255600	4.240700
C	0.093000	-3.609200	3.905600
C	-1.026500	-4.316300	3.426500
C	-2.251400	-3.622600	3.375000
C	-2.337900	-2.265000	3.707900
H	1.203900	-4.424300	-1.388400
H	-1.176800	-4.486200	-1.872600
H	-1.677600	-4.739500	0.577800
H	0.699700	-4.679400	1.078800
H	4.109000	-7.448900	-1.882100
H	2.657800	-7.280400	-4.136300
H	-1.317900	-7.348800	-4.971600
H	-3.532100	-7.604100	-3.474100
H	-4.342800	-8.014300	0.492200
H	-2.896200	-8.147300	2.750600
H	1.085500	-8.075900	3.556100
H	3.303900	-7.856200	2.057200
H	0.873900	-6.616200	-5.604900
H	0.069200	-5.162900	-6.194600
H	1.800200	-5.135100	-5.835600
H	-5.080000	-7.239300	-1.665600
H	-5.824200	-5.912600	-0.772400
H	-5.471500	-5.734200	-2.498100
H	-2.984800	-3.501700	-0.850800
H	-4.275400	-3.626200	-2.036800
H	-4.628600	-3.804900	-0.308900

H	-1.153800	-7.642000	4.315900
H	-0.450200	-6.273600	5.174400
H	-2.182100	-6.301800	4.819900
H	4.812000	-7.014500	0.379500
H	5.432600	-5.485500	-0.243700
H	5.079800	-5.664000	1.482100
H	2.398200	-3.382600	0.261700
H	3.704100	-3.616200	1.413800
H	4.057300	-3.437600	-0.314100
H	2.650900	-3.294500	-3.781000
H	2.621800	-0.851300	-3.884300
H	-1.607300	-0.918500	-4.665900
H	-1.602400	-3.340400	-4.569000
H	-1.301500	1.146200	-3.610400
H	-0.957700	1.405400	-5.356500
H	-0.921900	3.595200	-4.452800
H	0.811300	3.155000	-4.512200
H	3.434900	5.605600	-1.193100
H	2.101700	7.675800	-1.269100
H	-0.306100	7.587700	-1.786100
H	-2.105200	6.063100	-3.156200
H	-2.187300	4.410200	-2.620800
H	-4.448700	7.049500	2.137300
H	-4.780600	8.220000	-0.005800
H	-3.595600	7.481100	-2.036300
H	-3.454600	5.380400	3.445800
H	-3.255800	3.869600	2.607200
H	2.426500	5.095500	3.740400
H	1.086500	7.162200	3.697800
H	-1.327400	7.071000	3.210700
H	2.613900	2.737500	3.695100
H	1.206400	1.779700	4.027400
H	1.469700	0.578500	-1.704800
H	0.578900	-0.751100	0.162000
H	0.620500	0.148900	2.450000
H	2.556300	2.462600	-2.576900
H	3.684500	3.279200	-1.543000
H	-1.504600	1.973500	0.017900
H	-3.057200	2.524100	0.674500
H	-2.701000	2.704300	-1.068100
H	4.825900	4.605900	1.584000
H	4.538700	2.998000	2.277600
H	4.895500	3.178500	0.532000
H	-2.769700	2.660300	4.587800
H	-1.179600	2.147800	5.228000
H	-2.693700	0.441800	3.200700
H	-3.065700	0.338900	4.957100
H	0.892100	-1.726400	4.578600
H	1.057100	-4.100800	4.016800
H	-3.162800	-4.129800	3.065300
H	-3.302200	-1.775900	3.626500

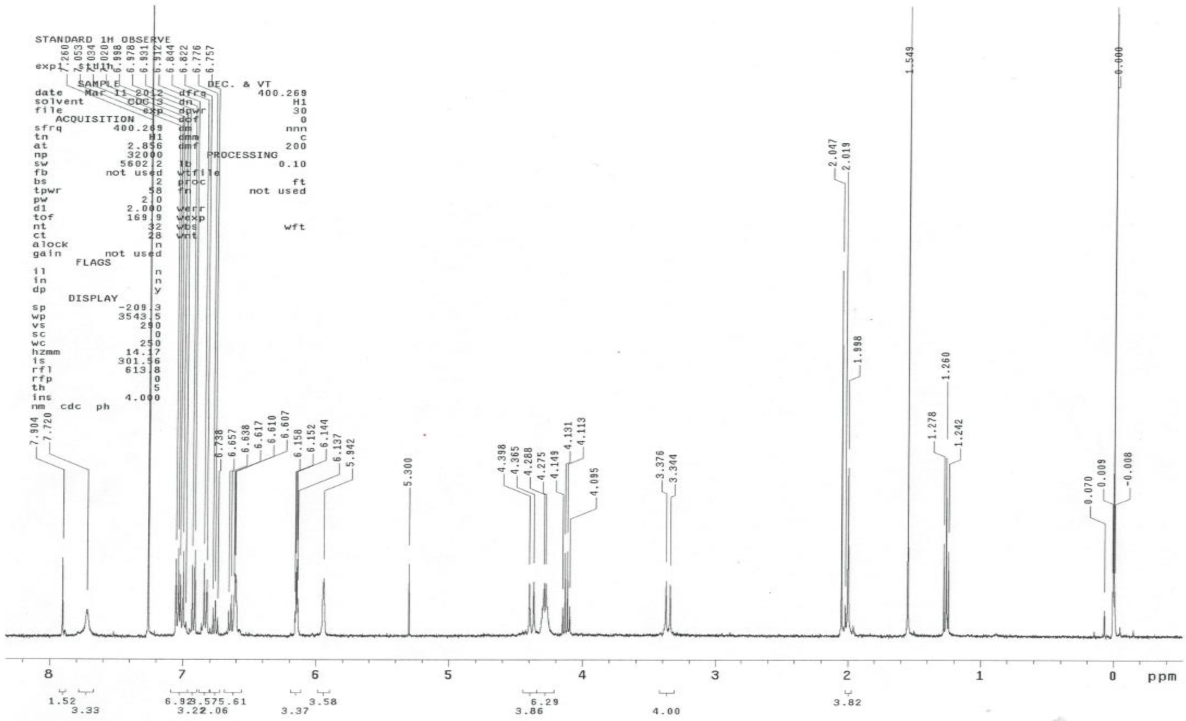

Figure S36. ^{1}H NMR spectrum of **6** recorded in $CDCl_3$.

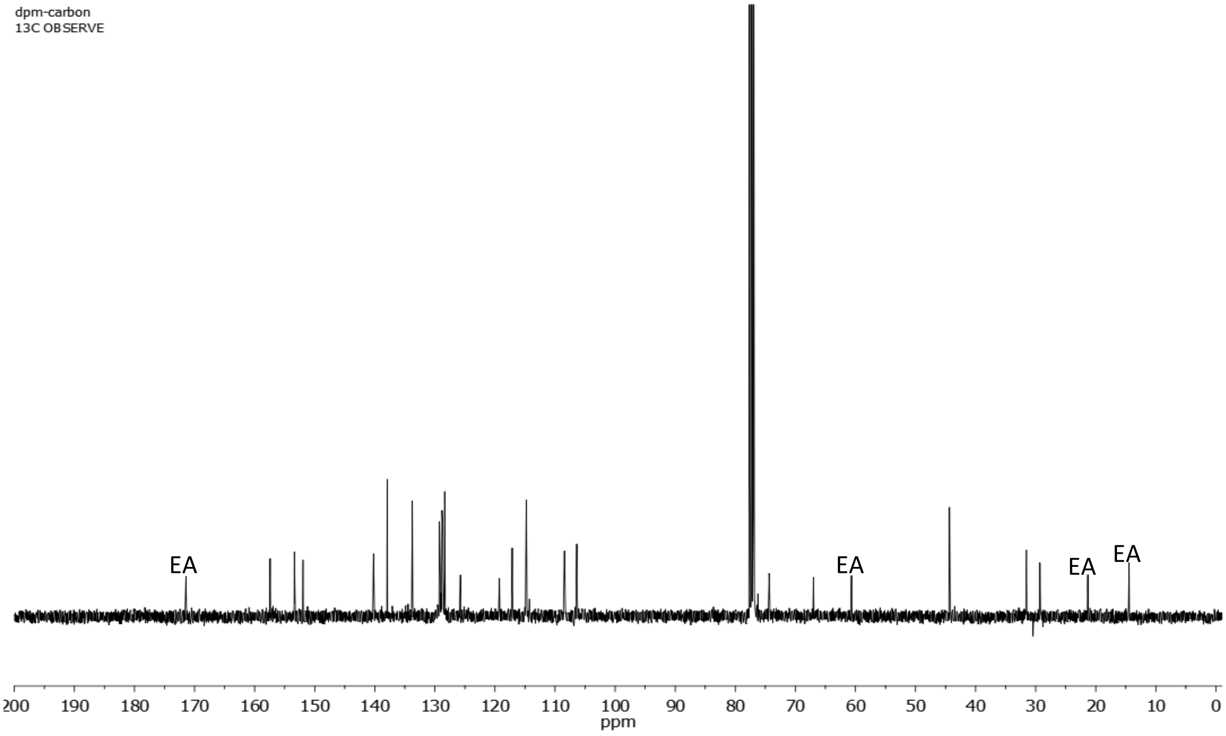

Figure S37. ^{13}C NMR spectrum of **6** recorded in $CDCl_3$.

Target Compound Screening Report

**Data File**	MSF1012-0522-hrESIpos1.d	**Sample Name**	MSF1012-0522	**Comment**	ksk1700
**Position**	P1-A8	**Instrument Name**	US10252005	**User Name**	
**Acq Method**	DualESIposMeOH_gt250_vcap3000.m	**Acquired Time**	11/9/2012 10:47:53 AM	**DA Method**	FindByFormula_22Nov2011.m

MS Zoomed Spectrum

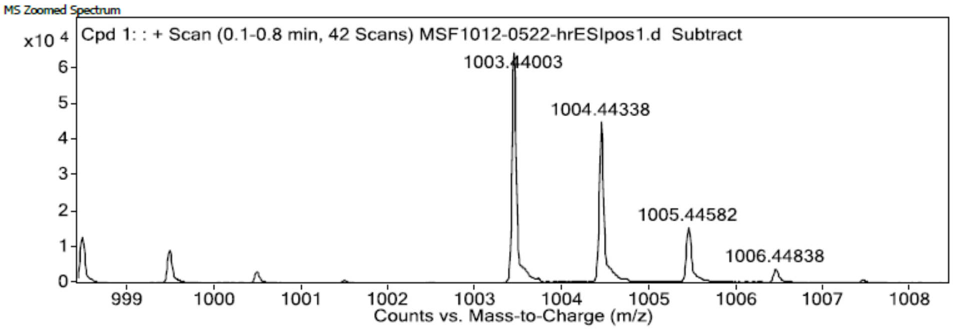

MS Spectrum Peak List

Obs. m/z	Calc. m/z	Charge	Abund	Formula	Ion/Isotope	Tgt Mass Error (ppm)
1003.44003	1003.44051	1	64161	C64H60N4NaO6	(M+Na)+	-0.47
1004.44338	1004.44376	1	45083.1	C64H60N4NaO6	(M+Na)+	-0.38
1005.44582	1005.44691	1	15803.8	C64H60N4NaO6	(M+Na)+	-1.08
1006.44838	1006.44998	1	4047.3	C64H60N4NaO6	(M+Na)+	-1.59
1007.45089	1007.45297	1	845.2	C64H60N4NaO6	(M+Na)+	-2.06
1008.45349	1008.45591	1	139.6	C64H60N4NaO6	(M+Na)+	-2.4

--- End Of Report ---

Figure S38. HRMS (ESI) of **6**.

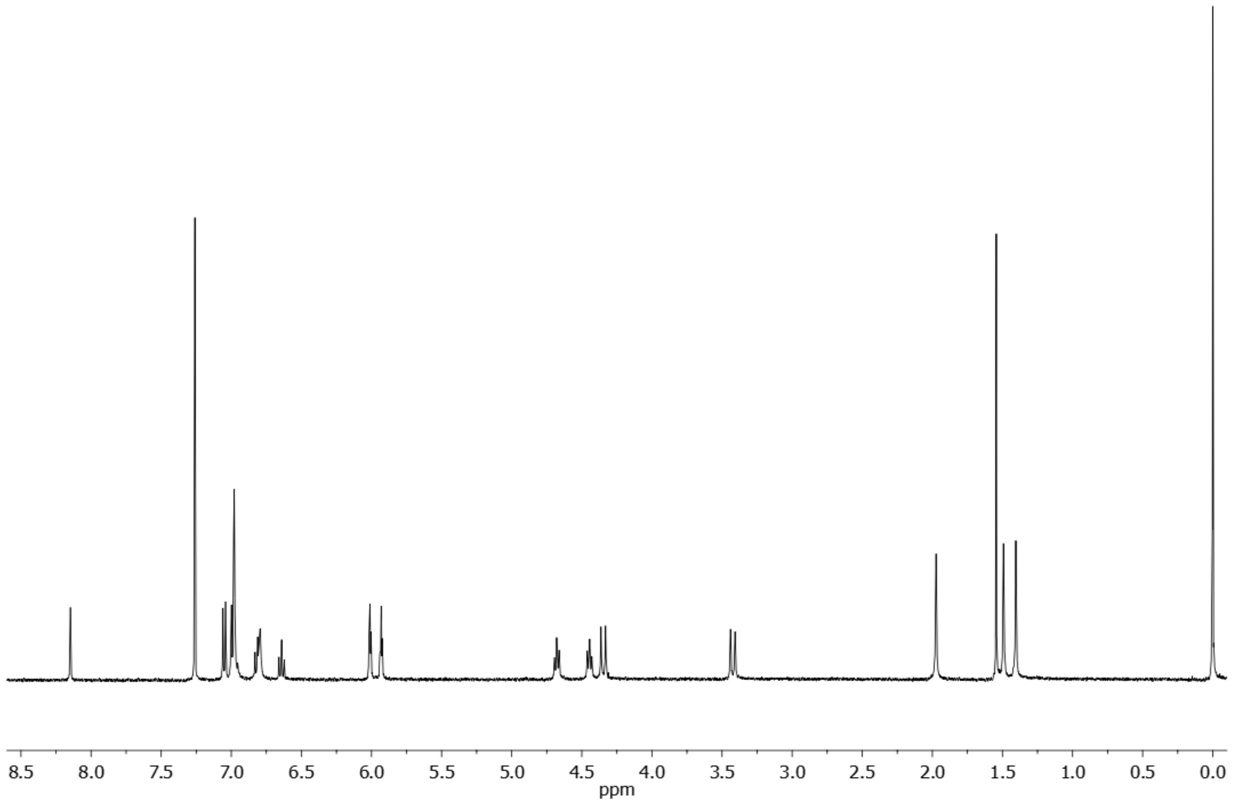

Figure S39. ^{1}H NMR spectrum of **1** recorded in $CDCl_3$.

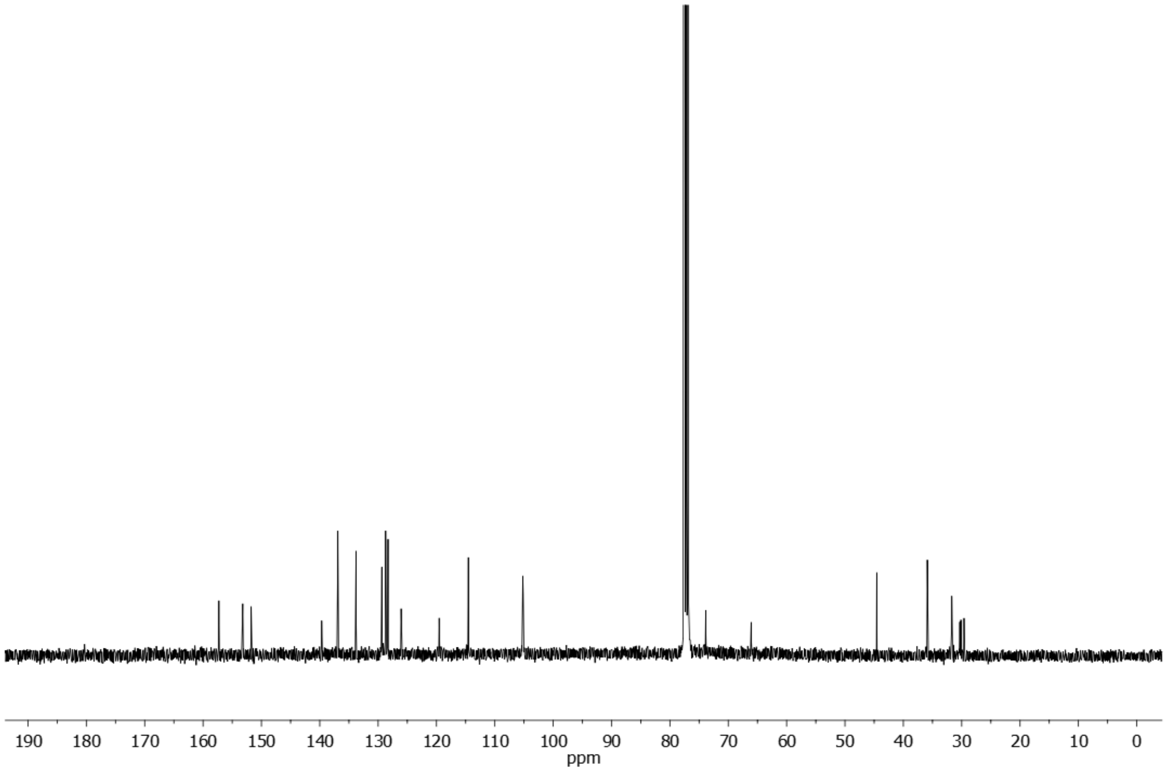

Figure S40. ^{13}C NMR spectrum of **1** recorded in $CDCl_3$.

Target Compound Screening Report

**Data File**	MSF14-2436(dihydroxy)_hrESIpos1.d	**Sample Name**	2436(dihydroxy)	**Comment**	2436(dihydroxy)
**Position**	P1-A6	**Instrument Name**	Instrument 1	**User Name**	
**Acq Method**	pos.m	**Acquired Time**	8/11/2014 10:30:37 AM	**DA Method**	Ian.m

MS Zoomed Spectrum

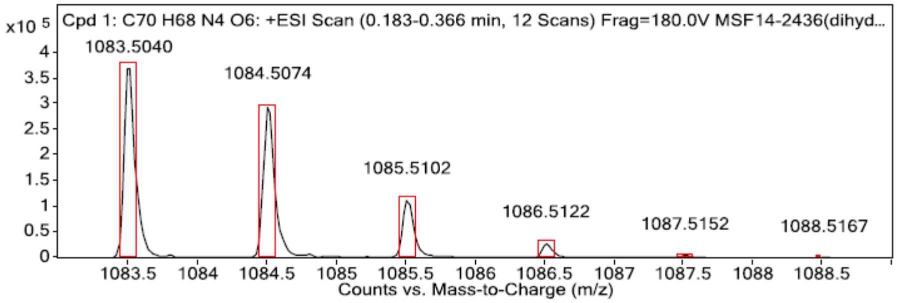

MS Spectrum Peak List

Obs. m/z	Calc. m/z	Charge	Abund	Formula	Ion/Isotope	Tgt Mass Error (ppm)
1083.50400	1083.50310	1	380054.39	C70H68N4O6	(M+Na)+	-0.86
1084.50740	1084.50640	1	299462.68	C70H68N4O6	(M+Na)+	-0.95
1085.51020	1085.50960	1	114375.12	C70H68N4O6	(M+Na)+	-0.55
1086.51220	1086.51270	1	29301.68	C70H68N4O6	(M+Na)+	0.45
1087.51520	1087.51570	1	5416.7	C70H68N4O6	(M+Na)+	0.42
1088.51670	1088.51870	1	882.26	C70H68N4O6	(M+Na)+	1.78
1089.50130	1089.52160	1	191.21	C70H68N4O6	(M+Na)+	18.65

--- End Of Report ---

Figure S41. HRMS (ESI) of **1**.

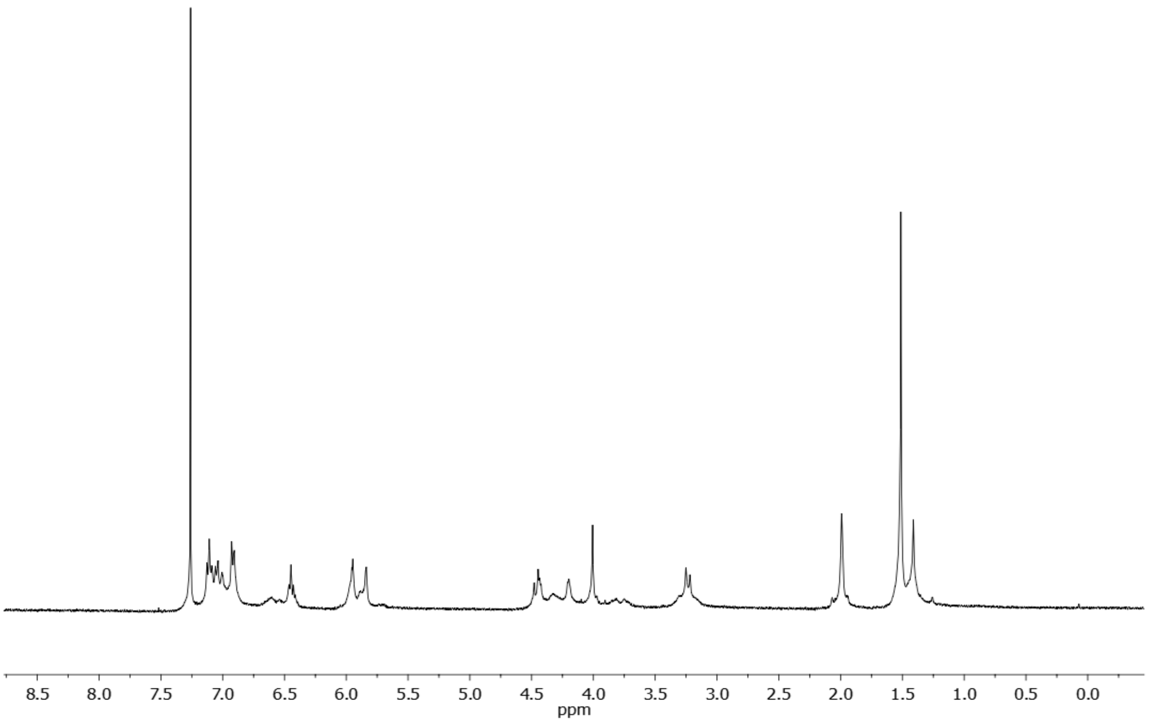

Figure S42. ^{1}H NMR spectrum of **2** recorded at room temperature in $CDCl_3$.

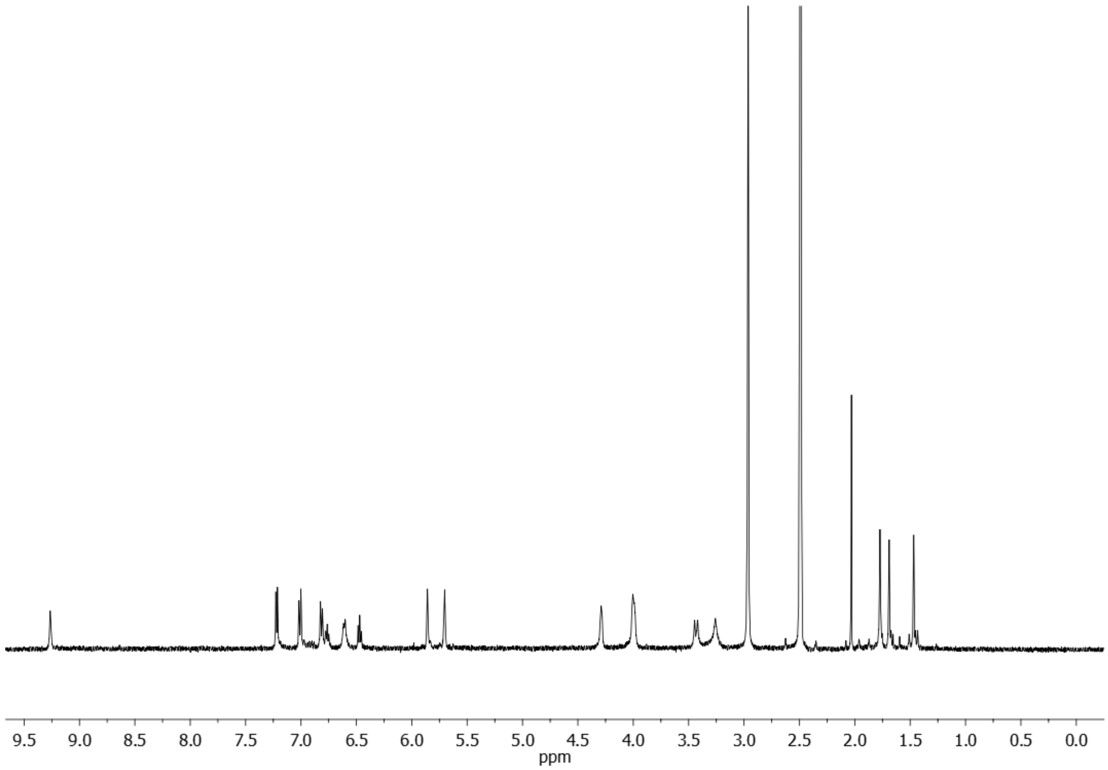

Figure S43. ^{1}H NMR spectrum of **2** recorded at 100 °C in DMSO-d_6.

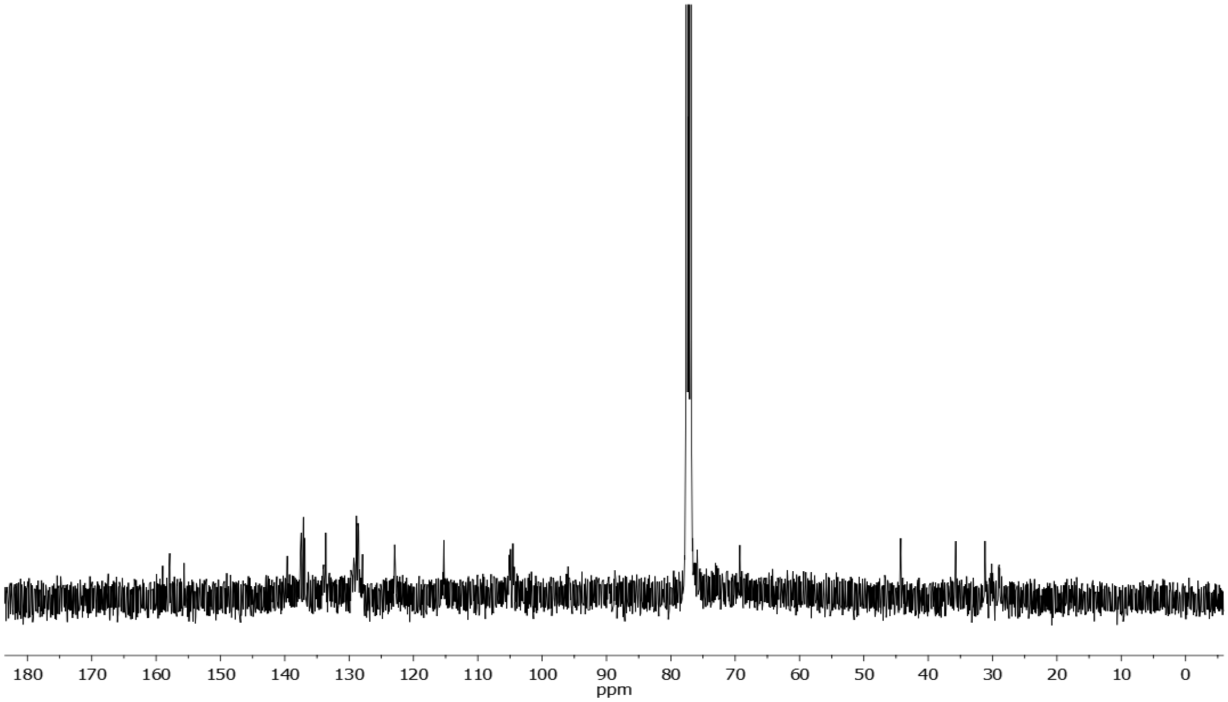

Figure S44. ^{13}C NMR spectrum of **2** recorded in $CDCl_3$.

Target Compound Screening Report

**Data File**	MSF14-1716(ksk1903)_hrESIpos1.d	**Sample Name**	1716	**Comment**	ksk1903
**Position**	P1-C9	**Instrument Name**	Instrument 1	**User Name**	
**Acq Method**	pos.m	**Acquired Time**	6/5/2014 11:27:17 AM	**DA Method**	Ian.m

MS Zoomed Spectrum

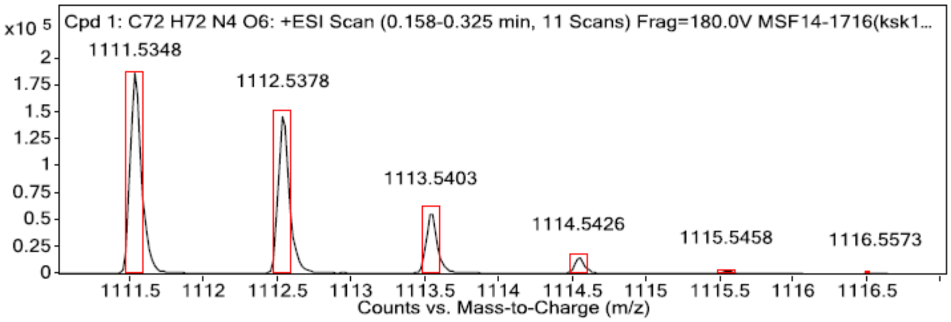

MS Spectrum Peak List

Obs. m/z	Calc. m/z	Charge	Abund	Formula	Ion/Isotope	Tgt Mass Error (ppm)
1106.57940			356479.74			
1111.53480	1111.53440	1	187524.01	C72H72N4O6	(M+Na)+	-0.34
1112.53780	1112.53770	1	148578.86	C72H72N4O6	(M+Na)+	-0.07
1113.54030	1113.54090	1	58105.96	C72H72N4O6	(M+Na)+	0.48
1114.54260	1114.54400	1	15614.57	C72H72N4O6	(M+Na)+	1.21
1115.54580	1115.54700	1	3114.73	C72H72N4O6	(M+Na)+	1.12
1116.55730	1116.55000	1	1046.24	C72H72N4O6	(M+Na)+	-6.49
1117.57530	1117.55300	1	1507.55	C72H72N4O6	(M+Na)+	-19.96

--- End Of Report ---

Figure S45. HRMS (ESI) of **2**.

References

1. S. K. Kim, H. G. Lee, G. I. Vargas-Zúñiga, V. M. Lynch, C. Kim, J. L. Sessler, *Chem. Eur. J.* 2014, **20**, 11750.
2. DENZO-SMN (1997). Z. Otwinowski and W. Minor Methods in Enzymology, 276: Macromolecular Crystallography, part A, 307 – 326, C. W. Carter, Jr. and R. M. Sweets, Editors, Academic Press.
3. SIR97. A program for crystal structure solution. A. Altomare, M. C. Burla, M. Camalli, G. L. Cascarano, C. Giacovazzo, A. Guagliardi, A. G. G. Moliterni, G. Polidori, R. Spagna, *J. Appl. Cryst.* 1999, **32**, 115.
4. G. M. Sheldrick (1994). SHELXTL/PC (Version 5.03). Siemens Analytical X-ray Instruments, Inc., Madison, Wisconsin, USA.
5. P. v. d. Sluis, A. L. Spek, SQUEEZE. *Acta Cryst.* 1999, **A46**, 194.
6. WinGX 1.64. An Integrated System of Windows Programs for the Solution, Refinement and Analysis of Single Crystal X-ray Diffraction Data. L. J. J. Farrugia, *Appl. Cryst.* 1999, **32**. 837-838.
7. A. L. Spek (1998). PLATON, A Multipurpose Crystallographic Tool. Utrecht University, The Netherlands.
8. $Rw(F^2) = \{\Sigma w(|F_o|^2 - |F_c|^2)^2/\Sigma w(|F_o|)^4\}^{1/2}$ where w is the weight given each reflection. $R(F) = \Sigma(|F_o| - |F_c|)/\Sigma|F_o|\}$ for reflections with $F_o > 4(\sigma(F_o))$. $S = [\Sigma w(|F_o|^2 - |F_c|^2)^2/(n - p)]^{1/2}$, where n is the number of reflections and p is the number of refined parameters.
9. International Tables for X-ray Crystallography (1992). Vol. C, Tables 4.2.6.8 and 6.1.1.4, A. J. C. Wilson, editor, Boston: Kluwer Academic Press.
10. G. M. Sheldrick, SHELXL97. Program for the Refinement of Crystal Structures. *Acta Cryst.* 2008, **A64**, 112-122.
11. CrystalClear 1.40 (2008). Rigaku Americas Corportion, The Woodlands, TX.
12. CrysAlisPro. Agilent Technologies (2013). Agilent Technologies UK Ltd., Oxford, UK, SuperNova CCD System, CrysAlicPro Software System, 1.171.37.31.
13. SAINT V8.27B Bruker AXS Inc, (2012), Madison, WI.
